# Identification and validation of loss of function variants in clinical contexts

**DOI:** 10.1002/mgg3.42

**Published:** 2013-10-11

**Authors:** Francesco Lescai, Elena Marasco, Chiara Bacchelli, Philip Stanier, Vilma Mantovani, Philip Beales

**Affiliations:** 1 University College London, Institute of Child Health, GOSgene teamLondon, U.K; 2 Department of Biomedicine, Human Genetics, Aarhus UniversityAarhus, Denmark; 3 CRBA Centro Ricerca Biomedica Applicata, Azienda Ospedaliero-Universitaria Policlinico S. Orsola – MalpighiBologna, Italy

**Keywords:** GATK, pipelines, sequencing, variant calling

## Abstract

The choice of an appropriate variant calling pipeline for exome sequencing data is becoming increasingly more important in translational medicine projects and clinical contexts. Within GOSgene, which facilitates genetic analysis as part of a joint effort of the University College London and the Great Ormond Street Hospital, we aimed to optimize a variant calling pipeline suitable for our clinical context. We implemented the GATK/Queue framework and evaluated the performance of its two callers: the classical UnifiedGenotyper and the new variant discovery tool HaplotypeCaller. We performed an experimental validation of the loss-of-function (LoF) variants called by the two methods using Sequenom technology. UnifiedGenotyper showed a total validation rate of 97.6% for LoF single-nucleotide polymorphisms (SNPs) and 92.0% for insertions or deletions (INDELs), whereas HaplotypeCaller was 91.7% for SNPs and 55.9% for INDELs. We confirm that GATK/Queue is a reliable pipeline in translational medicine and clinical context. We conclude that in our working environment, UnifiedGenotyper is the caller of choice, being an accurate method, with a high validation rate of error-prone calls like LoF variants. We finally highlight the importance of experimental validation, especially for INDELs, as part of a standard pipeline in clinical environments.

## Introduction

While exome sequencing is becoming a more widely accessible and available tool in the context of translational medicine research and in clinical diagnosis (Need et al. 2012), the choice of an accurate and reliable pipeline is of fundamental importance. The clinical environment has additional pressure to reduce the number of false-positive variant calls, while keeping the sensitivity as high as possible (Ku et al. 2011; Flannick et al. 2012). As new analytical methods are developed, simply comparing the characteristics and quality of the calls alone is not sufficient. Experimental validation is essential in order to choose the solution best suited to the task.

Recent studies have highlighted an increased bias toward false-positive calls among loss-of-function (LoF) variants (Macarthur and Tyler-Smith 2010; Macarthur et al. 2012), that is, those polymorphisms likely to be more interesting from a functional point of view, and particularly relevant when analyzing rare diseases with familial inheritance patterns. While single-nucleotide variants (SNVs) are straightforward to annotate and validate, additional effort is needed for insertion/deletions (INDELs) (Lescai et al. 2012). We therefore particularly welcome improved methods for their accurate identification.

GOSgene is a joint effort of the University College London and the Great Ormond Street Hospital, which aims to offer clinicians the necessary expertise to use next-generation sequencing in their activities. A recent comparison effort showed a higher validation rate for the processing and calling pipeline developed at the Broad Institute (O'Rawe et al. 2013): We therefore decided to implement GATK (version 2.2-2-gf44cc4e) with the workflow manager Queue (version 2.2-2-gf44cc4e) (DePristo et al. 2011) in our cluster environment. The standard analysis uses the classic GATK UnifiedGenotyper as the variant caller. With the advent of a new variant caller, HaplotypeCaller, we also decided to compare its performance with the standard analysis. This report describes the workflow and experimental validation of the LoF variants called, either by both methods or those unique to one of the other.

## Methods

This study was based on exomes generated from 32 independent samples which were captured with Agilent (Santa Clara, CA) SureSelect version 4, and were sequenced with Illumina (San Diego, CA) HiSeq 2000, according to manufacturer's specifications. The samples were aligned to the Human reference version GRGh37/hg19 using BWA version 0.5.9 and then processed with GATK according to the best practices version 4 (Data S2), as suggested by the Broad Institute. The variants have been called using both UnifiedGenotyper and HaplotypeCaller methods. The comparisons have been carried out using the GATK walker “VariantEval” and the R software. The Venn diagrams have been generated with the R package “vennerable” (http://r-forge.r-project.org/projects/vennerable/).

We performed our validation using Sequenom MALDI-ToF technology as previously described in other studies (Chahrour et al. 2012; Wilm et al. 2012; Zhou et al. 2013). This method provides the opportunity to genotype a large number of loci in all our samples at the same time, and because GATK already integrates tools to speedup the design of the amplicons. The design was therefore performed in two steps: first, the GATK “ValidationAmplicons” walker was used to preselect novel loci and mask known variants using dbSNP 135 sites. The output, a Sequenom-friendly format, was used in the second step to design the amplicon and extension primers using the Sequenom Assay Design Suite version 1.0 with high multiplexing iPLEX presets. These two independent design steps ensured filtering of genomic regions with primer design issues and increased the efficiency of the selected assays. The genotype was performed according to manufacturer's standard protocols for iPLEX.

The analysis has been carried out on a high-performance computing cluster developed at the UCL Department of Computer Science, characterized by 785 nodes (4012 CPU cores), with 8 to 96GB memory per node, and 3000 GPU cores, for a total number of 7012 cores; the storage includes 146G SCSI to 500G SATA to 2×128G SSD per node and a centralized 1PB GPFS/Lustre/Titan3200 system. Two full-time bioinformaticians are responsible for code development, data processing, and initial analysis.

## Results

**Figure 1:**
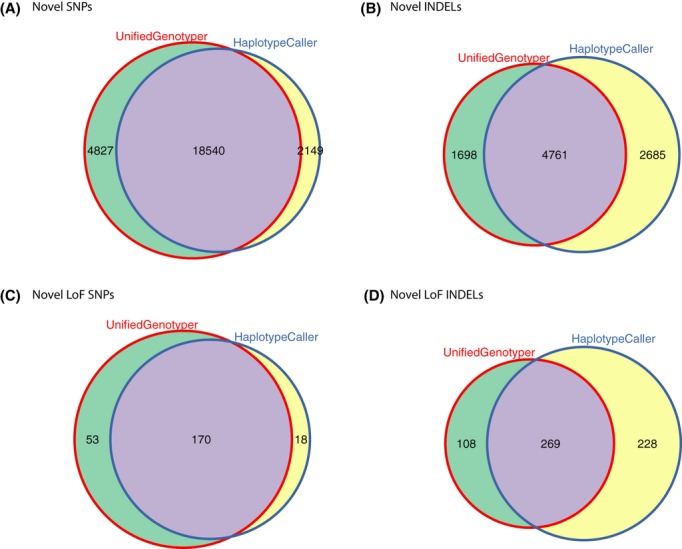
Comparison of the numbers of variants called by the UnifiedGenotyper and HaplotypeCaller. The specific variants identified by UnifiedGenotyper and HaplotypeCaller are shown as numbers within each circle. Variants common to both methods are in purple, whereas those unique to UnifiedGenotyper are in green and unique to HaplotyeCaller are in yellow. These represent (A) the total number of novel SNPs, (B) novel INDELs, (C) novel LoF SNPs, and (D) novel LoF INDELs (D).

In total, combining data obtained with the two approaches, we identified 25,516 novel single-nucleotide polymorphisms (SNPs) and 9144 novel INDELs (Fig. 1 and S1). We annotated our VCF files using Variant Annotation Tool version 2.0.1 (Habegger et al. 2012). For this analysis we focused on LoF variants, considering the importance of their likely biological consequences as well as their increased probability of being false positives. These included 241 novel LoF SNPs and 605 novel LoF INDELs (Fig. 1 and S1). The “CombineVariants” walker of GATK was used in order to identify the overlaps between the results of the variant callers (Intersection) and those variants unique to each method (“UnifiedGenotyper only” or “HaplotypeCaller only”, Fig. 1). In particular, 170 novel LoF SNPs and 269 novel LoF INDELs were identified by both callers, 53 SNPs and 108 INDELs by UnifiedGenotyper only, 18 SNPs and 228 INDELs by HaplotypeCaller only (Fig. 1).

In total we designed 140 assays for novel LoF SNPs and 96 assays for LoF INDELs, with an efficiency rate of 97.1% for the SNPs and 83.3% of the INDELs (Fig. S1). The percentage of assays that work successfully (50–58% for SNPs and 11–14% for INDELs, Table S1) was evenly distributed across the three categories (i.e., those unique to each caller and those that are identified by both methods), excluding the likelihood of a bias in the assay design for specific groups of variants.

The results of the validation show a validation rate of 98% for those SNPs called by both methods, 96.4% for the SNPs called only by UnifiedGenotyper, and 22.2% for those SNPs called by HaplotypeCaller only. When we consider the INDELs, the validation rate was 92.1% for those identified with both callers, 91.7% for those called by UnifiedGenotyper only, and 10% for those called by HaplotypeCaller only (Table 1).

**Table 1:** Validation of variants by caller comparison

Outcome	Intersection	UnifiedGenotyper only	HaplotypeCaller only
SNPs			
Validated	97 (98.0%)	27 (96.4%)	2 (22.2%)
Not validated	2 (2.0%)	1 (3.6%)	7 (77.8%)
Fail	3	0	1
Total number of assays	102	28	10
Total number of working assays	99	28	9
INDELs			
Validated	35 (92.1%)	11 (91.7%)	3 (10.0%)
Not validated	3 (7.9%)	1 (8.3%)	27 (90.0%)
Fail	4	0	12
Total number of assays	42	12	42
Total number of working assays	38	12	30

The validation rates of LoF SNPs and INDEL calls from both methods (intersection) or uniquely called by UnifiedGenotyper or HaplotypeCaller. The failure rate of validation assays (fail) on the genotyping chip is given.

**Figure 2:**
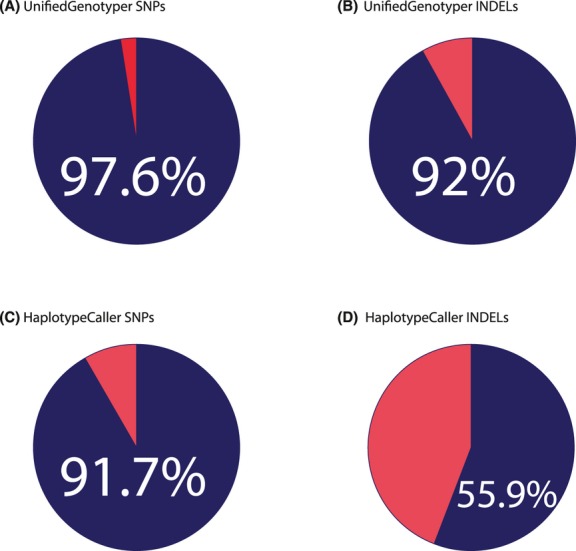
Validation rates by caller comparison. The pies show the overall validation rate for LoF SNPs and INDELs called by UnifiedGenotyper and by HaplotypeCaller. HaplotypeCaller INDELs showed the lowest validation rate of 55.9% of the called variants (D).

If we sum all the variants identified by each method separately, UnifiedGenotyper showed a total validation rate of 97.6% for LoF SNPs and 92.0% for LoF INDELs, and HaplotypeCaller of 91.7% for SNPs and 55.9% for INDELs (Fig. 2, and Table S2).

We notice that LoF SNP calls overlap significantly between the two methods (170, of 241) with UnifiedGenotyper calling more unique variants than did HaplotypeCaller (53 compared to 18, Fig. 1). The proportions differ for LoF INDEL calls though, where 71.3% of UnifiedGenotyper calls overlap but only 54.1% of HaplotypeCaller variants are called by both methods. In total, HaplotypeCaller identified more LoF INDELs than UnifiedGenotyper, that is, 497 compared to 377 of which 228 were unique (Fig. 1).

As expected, the validation rate for both SNP and INDEL LoF variants called by UnifiedGenotyper as well as by HaplotypeCaller was very high (98.0% for the SNPs and 92.1% for the INDELs). Significant differences are observed when we compare the variants that were identified by one of the methods only. For example, UnifiedGenotyper called more unique SNPs, but they were validated at a rate of 96.4% compared to only 22.2% for those unique to HaplotypeCaller (Table 1). The figures are very different also for the LoF INDEL calls unique to one caller. In this case, HaplotypeCaller identified more INDELs, but the validation rate was very poor at 10% compared to 91.7% of those identified only by UnifiedGenotyper (Table 1).

In terms of the validation, almost every SNP assay performed well using the MALDI-ToF. Assays for the HaplotypeCaller INDEL variants performed slightly worse. This might reflect an increase in calls located in genomic regions slightly more difficult to genotype. However, the percentage of working assays compared to the total LoF INDEL calls is quite similar between the HaplotypeCaller unique calls and those for UnifiedGenotyper (Table 1). These proportions indicate that there is no discernible design bias for the three categories (intersect or unique to each caller), which might otherwise influence the validation rate differences we observed.

After the experimental validation has been completed, a new version of HaplotypeCaller (2.4.9) has also been released: we therefore compared the overall performance of the calls with the previous versions and we checked which of the validated variants have been identified by the new one. For both LoF SNPs and INDELs, we notice a higher overlap with UnifiedGenotyper (96.6% and 70.7%, respectively, compared with 90.4% and 54.1% of the previous version, Fig. S2). Of the variants that have been experimentally validated, the higher overlap with UnifiedGenotyper increases the percentage of validated loci: The new caller, however, also overlap with 3 SNPs and 51 INDELs called by the previous version, which display a lower validation rate (60%, Table S3). As this version has been released after our genotyping, no data are available on the variants uniquely called by the new version of HaplotypeCaller compared to all others: On the opposite, 12.5% of the validated SNPs and 5.3% of the validated INDELs have not been called by the new HaplotypeCaller version.

In order to better understand the failing variants, we finally investigated the BAM files, and we could not identify a recurring pattern in the sequence or in the genomic context as a reason for miscalled loci. We inspected therefore in detail the annotations of the variants in order to identify qualitatively any parameters that might best distinguish validated from not validated calls, and the two methods from one another (Data S1). Culprit values identify the value, which the variants most differ for in the variant quality score recalibration step: It is therefore a good starting point to select the covariates that might influence most of the separation between false and positive calls. Three values are the culprit covariates for failing variants: Fisher Strand (FS, phred-scaled strand bias), mapping quality (MQ), and quality over depth (QD). Strand bias is a known characteristic of targeted capture at the boundaries of captured regions: Failing variants have a higher strand bias (lower phred-scaled value) for both callers. MQ also affects almost in the same way all type of variants for all callers, with failing variants being in regions of lower MQ. The most interesting parameter is, however, QD, defined as variant confidence (from the QUAL field in the VCF file) divided by unfiltered depth of nonreference samples. QD is also normalized by event length. When we analyzed QD density plots, we did not notice a great deal of difference in validated and not validated variants called by UnifiedGenotyper, but the distributions are clearly different (for both SNPs and INDELs) in the calls made by HaplotypeCaller (Data S1, page 8 and, for the VQSR plots in detail, Data S3).

## Conclusions

Overall we can confirm that the GATK/Queue pipeline is a reliable choice with a high validation rate, suitable in translational medicine and clinical contexts.

Considering the overall performance of the two callers, for SNP calls we do not see a great deal of difference with a large overlap and similar validation rate. This is not so for the INDEL calls: While UnifiedGenotyper showed a total validation rate of 92.0%, HaplotypeCaller validated 55.9% of them. The two methods identified less insertions/deletions in common, and although for HaplotypeCaller the overall validation rate is higher than the one of its uniquely called loci, its performance is currently not suitable for a clinical context.

The analysis of the characteristics of the failing variants showed that strand bias MQ and QD might influence the calling and contribute to the reasons for false-positive calling. QD in particular affects mostly HaplotypeCaller, indicating that the algorithm is likely to be more sensitive in regions of lower calling confidence combined with lower nonreference depth. These considerations might also have an impact for whole-genome sequencing analysis, where FS might be less important but MQ and QD will play a role if multisample calling is combined with lower sequencing depth to save costs. A more detailed analysis of these sequences and the respective algorithms will be required to shed further light on this problem.

In conclusion, we recognize that experimental validation of LoF variants should be considered as an important step in the identification of causative mutations in our environment, and the adoption of a stable pipeline is a prerequisite for the overall reliability. While we realize that HaplotypeCaller may have some promising features in calling multiple-nucleotide polymorphisms and INDELs, the tool is still in active development as the Broad Institute warns on the GATK website. We were able to compare a newer version after the validation of our data set has been completed, and we appreciated a higher overlap between the two callers and general improvements: the new HaplotypeCaller still does not identify a minor percentage of the validated variants. Newer releases are, however, frequently distributed, which make this tool suitable for research and discovery environments. For use within a clinical and translational research context, such as ours, GATK/Queue with UnifiedGenotyper is our pipeline of choice, which provides an accurate and stable method, with a high validation rate of error-prone calls as LoF variants.

## Conflict of Interest

None declared.

## Supporting Information

Data S1 Analysis of variants.

Data S2 Implementation of the best practices parameters in the Scala code.

Data S3 UnifiedGenotyper SNP VQSR.

Figure S1 Workflow.

Figure S2 Comparison with HaplotypeCaller version 2.4.9.

**Table S1**Design. **Table S2**Validation of variants by caller. **Table S3**Performance of the new HaplotypeCaller version on genotyped variants.
