# Supplementary material for: Identification and validation of loss of function variants in clinical contexts
Source: Mol Genet Genomic Med. 2013 Oct 11;2(1):58–63. doi: 10.1002/mgg3.42 (PMC3907911; doi:10.1002/mgg3.42)
Supplement: Data S1 — Analysis of variants. [file mgg30002-0058-sd1.pdf]

Supplementary Material 1: analysis of variants characteristics by validation

Francesco Lescai

August 26, 2013

Contents

1 Introduction **3**

2 Variants Characteristics **4**

2.1 Variant Quality recalibration LOD score 4
2.2 Culprit values 6
2.3 Strand bias values 7
2.4 Quality over Depth 8
2.5 Mapping Quality values 9
2.6 Haplotype Score values 10
2.7 Depth distribution 11
2.8 GC content distribution 12

1 Introduction

In order to better characterise the variants that didn't validate in the genotyping, a number of annotations are compared in the following pages. The readability of the plots has been improved by presenting the data according to the validation result and the caller of origin: for most of the annotations, the values will depend on the calling process and the variant quality score recalibration, and therefore also the overlapping variants (i.e. those called by both methods) have been presented separately. In GC content instead, which is an independent genome characteristic, more emphasis has been put on the distinction between the overlap and the calls unique to one of the two methods.
The comparison here presented is meant to be qualitative, and should help in identify potential reasons for calling errors.

2 Variants Characteristics

2.1 Variant Quality recalibration LOD score

In terms of LOD score, no striking differences can be observed if we stratify the variants according to caller and overlap. In some density plots, the very small number of variants does not allow to see the both distributions.

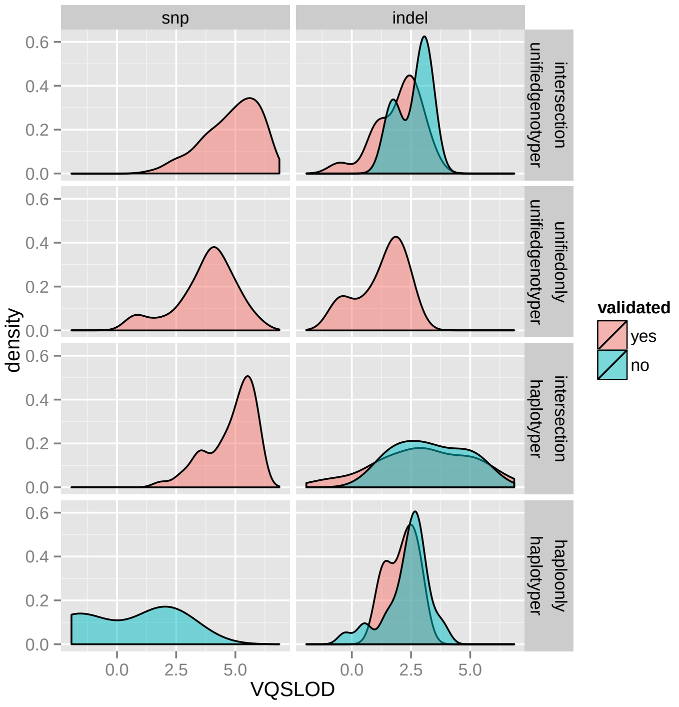

Figure 1: VQSLOD distribution by caller, overlap and validation

A more simplified view allows to notice that in HaplotypeCaller, and for SNPs only, variants that did not validate tend to have a smaller LOD score.

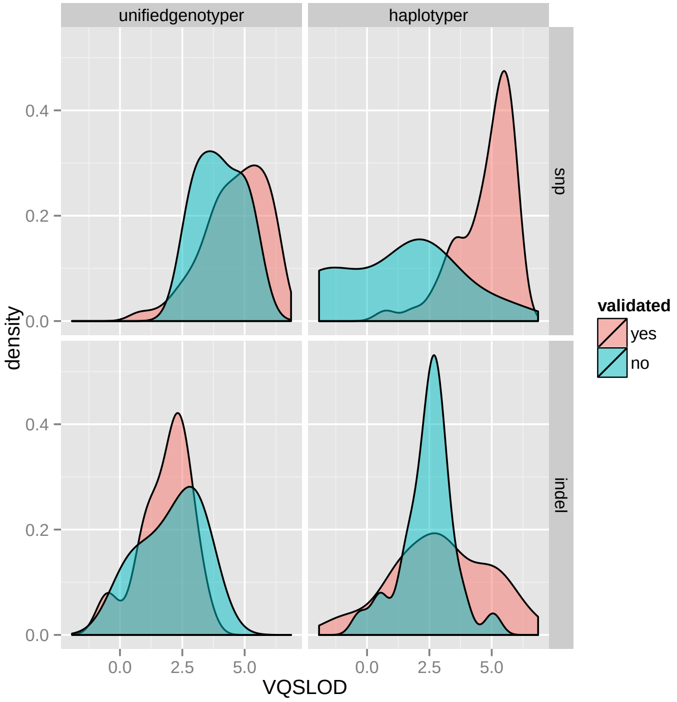

Figure 2: VQSLOD distribution by caller and validation only

2.2 Culprit values

Culprit values are the parameters which the variants most differ for: identifying the culprit values for not validated variants might help clarifying the major issues behind the calling errors.
In this case, the three most represented values among false calls are: FS (phred-scaled strand bias measure), MQ (mapping quality) and QD (quality over depth).

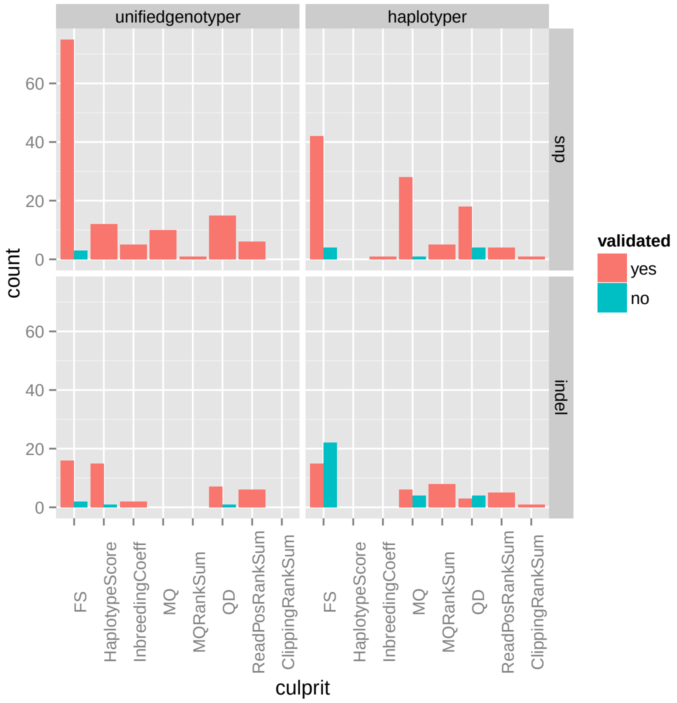

Figure 3: Count of validated vs not-validated variants by Culprit value

We will see each of these parameters in the next pages.

2.3 Strand bias values

In general, but this is more evident for SNPs called by UnifiedGenotyper and INDELs called by HaplotypeCaller, not validated variants have a higher strand bias: this might highlight a commonly known issue in sequencing capture regions where sequencing data are mostly present only in forward or reverse.

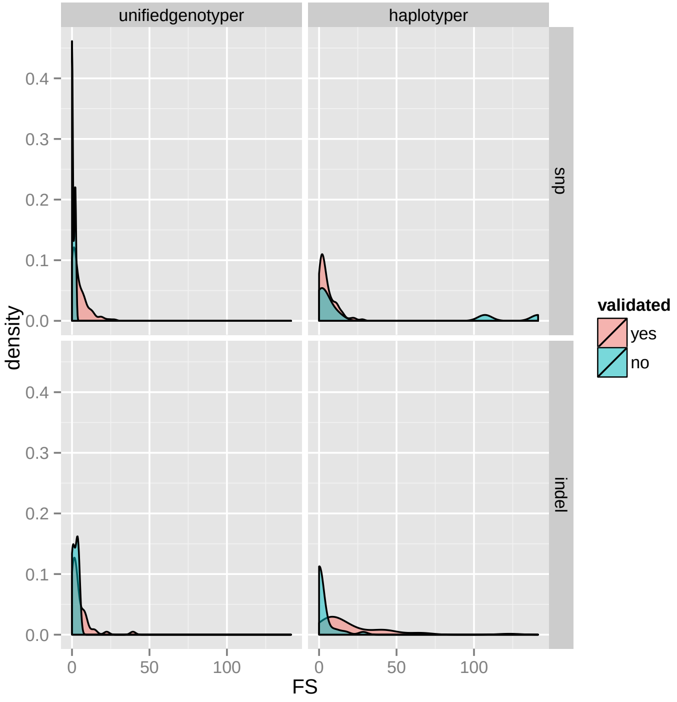

Figure 4: Density distribution of strand-bias FS score by caller and validation

2.4 Quality over Depth

While in general one could expect lower depths to influence false calling, in this case it is interesting to notice that HaplotypeCaller is more affected by the Quality over Depth combination. This is true both for SNPs and for INDELs, and it might be a characteristic of the algorithm.

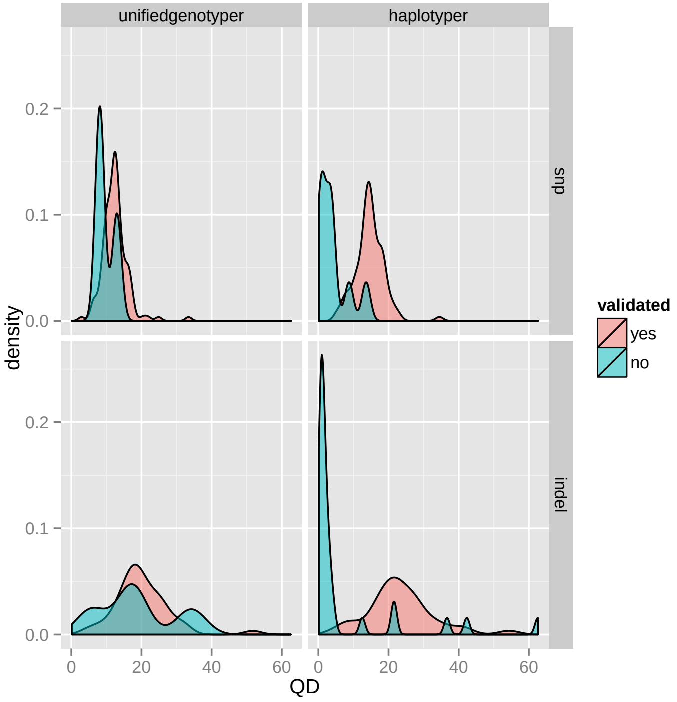

Figure 5: Density distribution of quality-over-depth value by caller and validation

2.5 Mapping Quality values

Mapping Quality clearly affects the calling in all cases: variant type, and calling method. The different density distribution of this parameter between validated and not validated variants is evident.

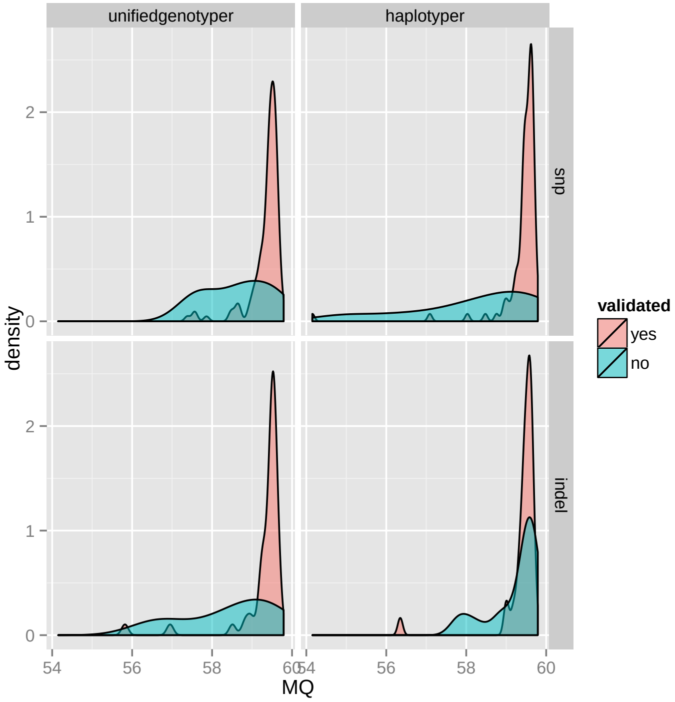

Figure 6: Density distribution of Mapping Quality by caller and validation

2.6 Haplotype Score values

There is a wide range of values for the HaplotypeScore and it is not easy to make the plots readable. In the following figure the counts of the variants called by bins of 0.5 of Score value have been plotted, with a limit on the X axis to 50. The only major difference here is that HaplotypeCaller results in a much flatter distribution of HaplotypeScores than UnifiedGenotyper.

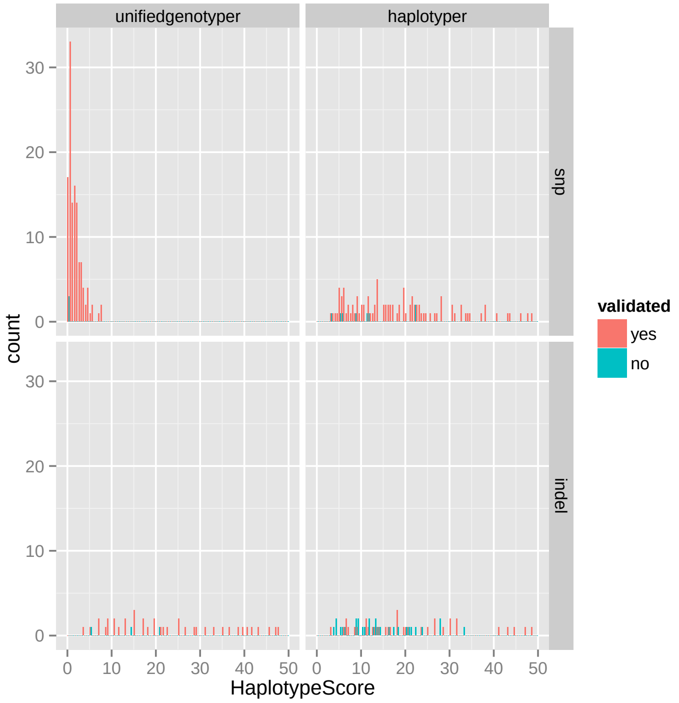

Figure 7: Distribution of variants count per Haplotype Score value by caller and validation, limited to max = 50

2.7 Depth distribution

While it is generally clear that not validated variants are called in lower depth region (and this very evident for SNPs called by UnifiedGenotyper), this measure does not highlight a clear difference of performance between the two callers as it happens for the Quality over Depth.

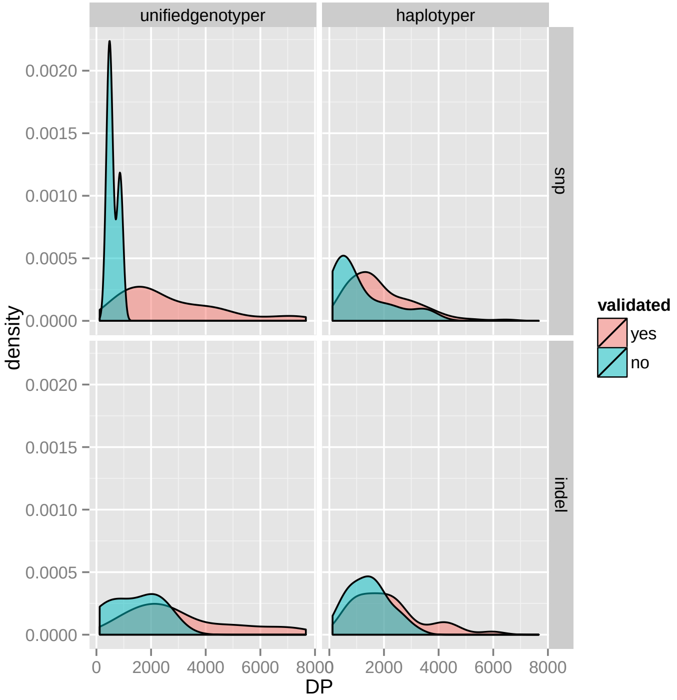

Figure 8: Density distribution of depth value of the variants, by caller and validation

2.8 GC content distribution

In this plot we cannot identify major differences for the GC content value in not validated variants: they are called in a wide range of values and the not validated variants do not seem to differ for particularly high or low values.

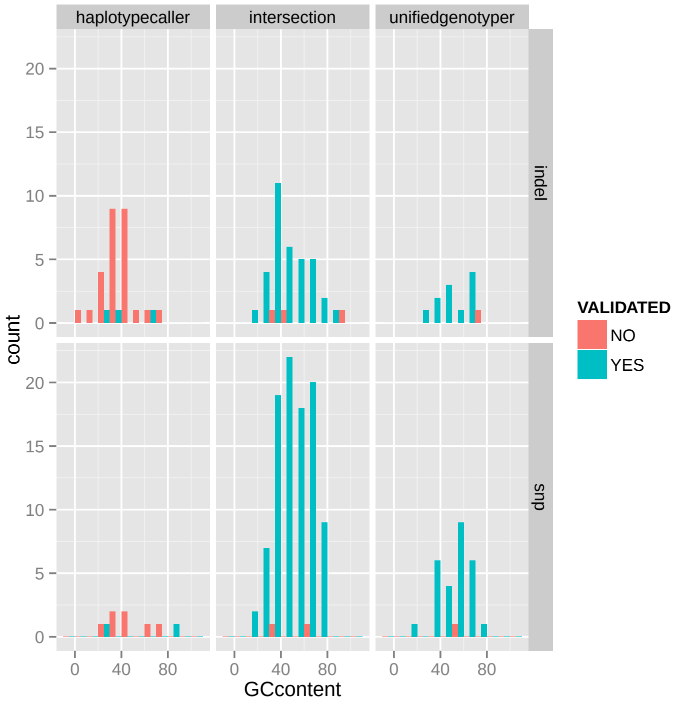

Figure 9: Distribution of variants count by GC content percentage and validation

```
> sessionInfo()

R version 2.15.1 (2012-06-22)
Platform: x86_64-apple-darwin9.8.0/x86_64 (64-bit)

locale:
[1] en_GB.UTF-8/en_GB.UTF-8/en_GB.UTF-8/C/en_GB.UTF-8/en_GB.UTF-8

attached base packages:
[1] stats     graphics  grDevices utils     datasets  methods   base

other attached packages:
[1] ggplot2_0.9.3.1          VariantAnnotation_1.4.12 Rsamtools_1.10.2
[4] Biostrings_2.26.3        GenomicRanges_1.10.7     IRanges_1.16.6
[7] BiocGenerics_0.4.0

loaded via a namespace (and not attached):
 [1] AnnotationDbi_1.20.7   Biobase_2.18.0         biomaRt_2.14.0
 [4] bitops_1.0-4.2         BSgenome_1.26.1        colorspace_1.2-1
 [7] DBI_0.2-5              dichromat_2.0-0        digest_0.6.3
[10] GenomicFeatures_1.10.2 grid_2.15.1            gtable_0.1.2
[13] labeling_0.1           MASS_7.3-23            munsell_0.4
[16] parallel_2.15.1        plyr_1.8               proto_0.3-10
[19] RColorBrewer_1.0-5     RCurl_1.95-4.1         reshape2_1.2.2
[22] RSQLite_0.11.2         rtracklayer_1.18.2     scales_0.2.3
[25] stats4_2.15.1          stringr_0.6.2          tools_2.15.1
[28] XML_3.96-1.1           zlibbioc_1.4.0
```
