# Supplementary material for: Identification and validation of loss of function variants in clinical contexts
Source: Mol Genet Genomic Med. 2013 Oct 11;2(1):58–63. doi: 10.1002/mgg3.42 (PMC3907911; doi:10.1002/mgg3.42)
Supplement: Data S2 — Implementation of the best practices parameters in the Scala code. [file mgg30002-0058-sd2.docx]

**Supplementary Material 2: implementation of the best practices parameters in the Scala code**

**Version: November 2012**

**Training and Reference**

reference = ftp.broadinstitute.org/bundle/1.5/b37/human_g1k_v37.fasta

dbSNP_b37 = ftp.broadinstitute.org/bundle/1.5/b37/dbsnp_135.b37.vcf

hapmap_b37 = ftp.broadinstitute.org/bundle/1.5/b37/hapmap_3.3.b37.sites.vcf

omni_b37 = ftp.broadinstitute.org/bundle/1.5/b37/1000G_omni2.5.b37.sites.vcf

mills_b37 = ftp.broadinstitute.org/bundle/1.5/b37/Mills_and_1000G_gold_standard.indels.b37.sites.vcf

**HaplotypeCaller VQSR**

this.resource :+= new TaggedFile( hapmap_b37, "hapmap,known=false,training=true,truth=true,prior=15.0" )

this.resource :+= new TaggedFile( omni_b37, "omni,known=false,training=true,truth=false,prior=12.0" )

this.resource :+= new TaggedFile( dbSNP_b37, "dbsnp,known=true,training=false,truth=false,prior=6.0" )

this.resource :+= new TaggedFile( mills_b37, "mills,known=true,training=true,truth=true,prior=12.0" )

this.use_annotation ++= List("QD", "MQRankSum", "ReadPosRankSum", "FS", "MQ", "ClippingRankSum")

if(t.nSamples >= 10) {

this.use_annotation ++= List("InbreedingCoeff")

this.maxGaussians = 6

}

else {

this.maxGaussians = 4

this.percentBad = 0.04

}

ts_filter_level = 97.0

**UnifiedGenotyper VQSR SNPs**

this.resource :+= new TaggedFile( hapmap_b37, "hapmap,known=false,training=true,truth=true,prior=15.0" )

this.resource :+= new TaggedFile( omni_b37, "omni,known=false,training=true,truth=false,prior=12.0" )

this.resource :+= new TaggedFile( dbSNP_b37, "dbsnp,known=true,training=false,truth=false,prior=6.0" )

this.use_annotation ++= List("QD", "HaplotypeScore", "MQRankSum", "ReadPosRankSum", "FS", "MQ")

if(t.nSamples >= 10) {

this.use_annotation ++= List("InbreedingCoeff")

this.maxGaussians = 6

}

else {

this.maxGaussians = 4

this.percentBad = 0.04

}

**UnifiedGenotyper VQSR INDELs**

this.maxGaussians = 4

this.std = 10.0

this.resource :+= new TaggedFile( mills_b37, "mills,known=true,training=true,truth=true,prior=12.0" )

this.use_annotation ++= List("QD", "FS", "HaplotypeScore", "ReadPosRankSum")

if(t.nSamples >= 10) {

this.use_annotation ++= List("InbreedingCoeff")

this.percentBad = 0.12

}

else {

this.percentBad = 0.04

}
