# Supplementary material for: Identification and validation of loss of function variants in clinical contexts
Source: Mol Genet Genomic Med. 2013 Oct 11;2(1):58–63. doi: 10.1002/mgg3.42 (PMC3907911; doi:10.1002/mgg3.42)
Supplement: Data S3 — UnifiedGenotyper SNP VQSR. [file mgg30002-0058-sd3.pdf]

UnifiedGenotyper SNP VQSR

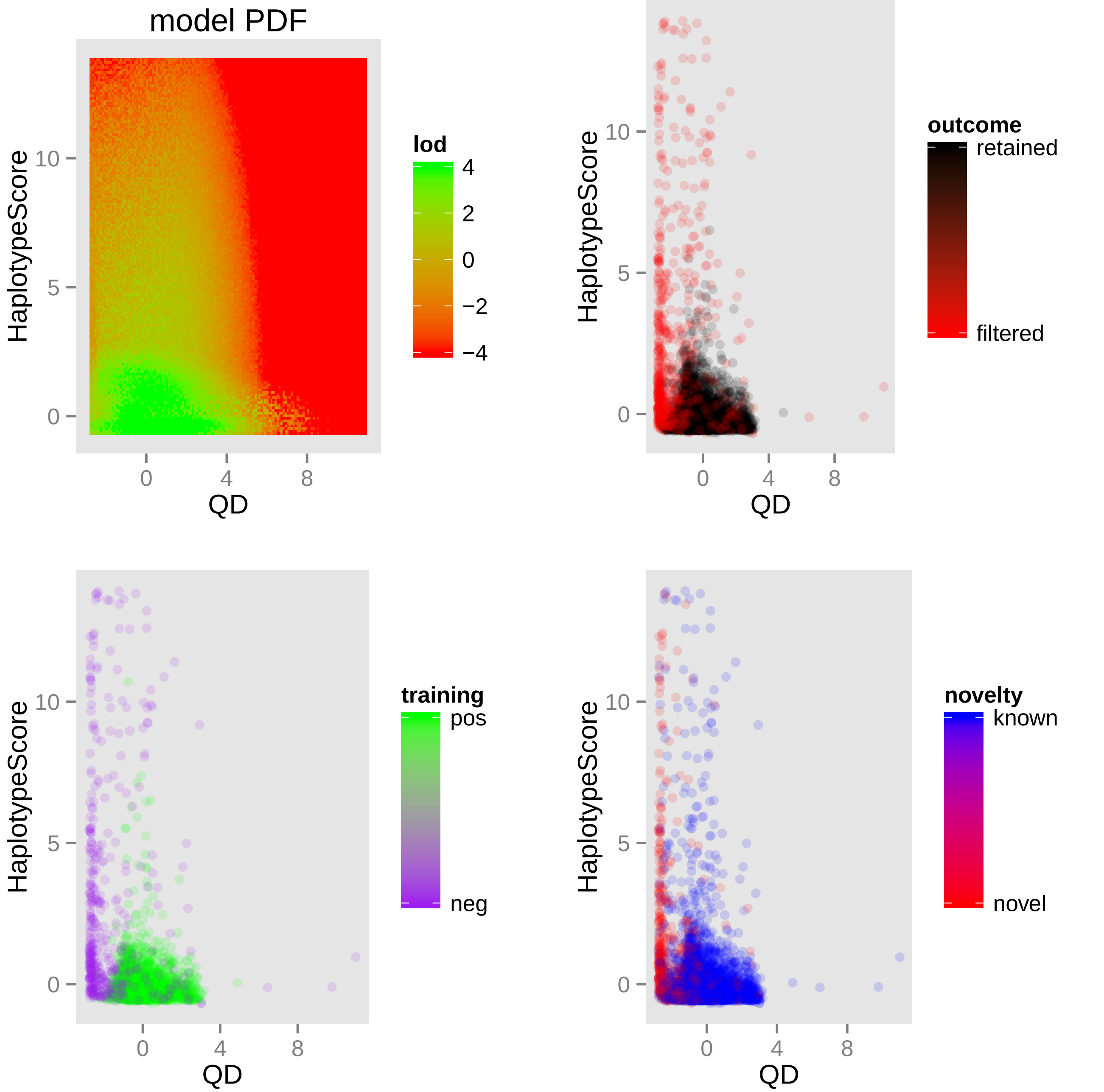
model PDF
HaplotypeScore
QD
lod
4
2
0
−2
−4
outcome
retained
filtered
training
pos
neg
novelty
known
novel
10
5
0
4
8

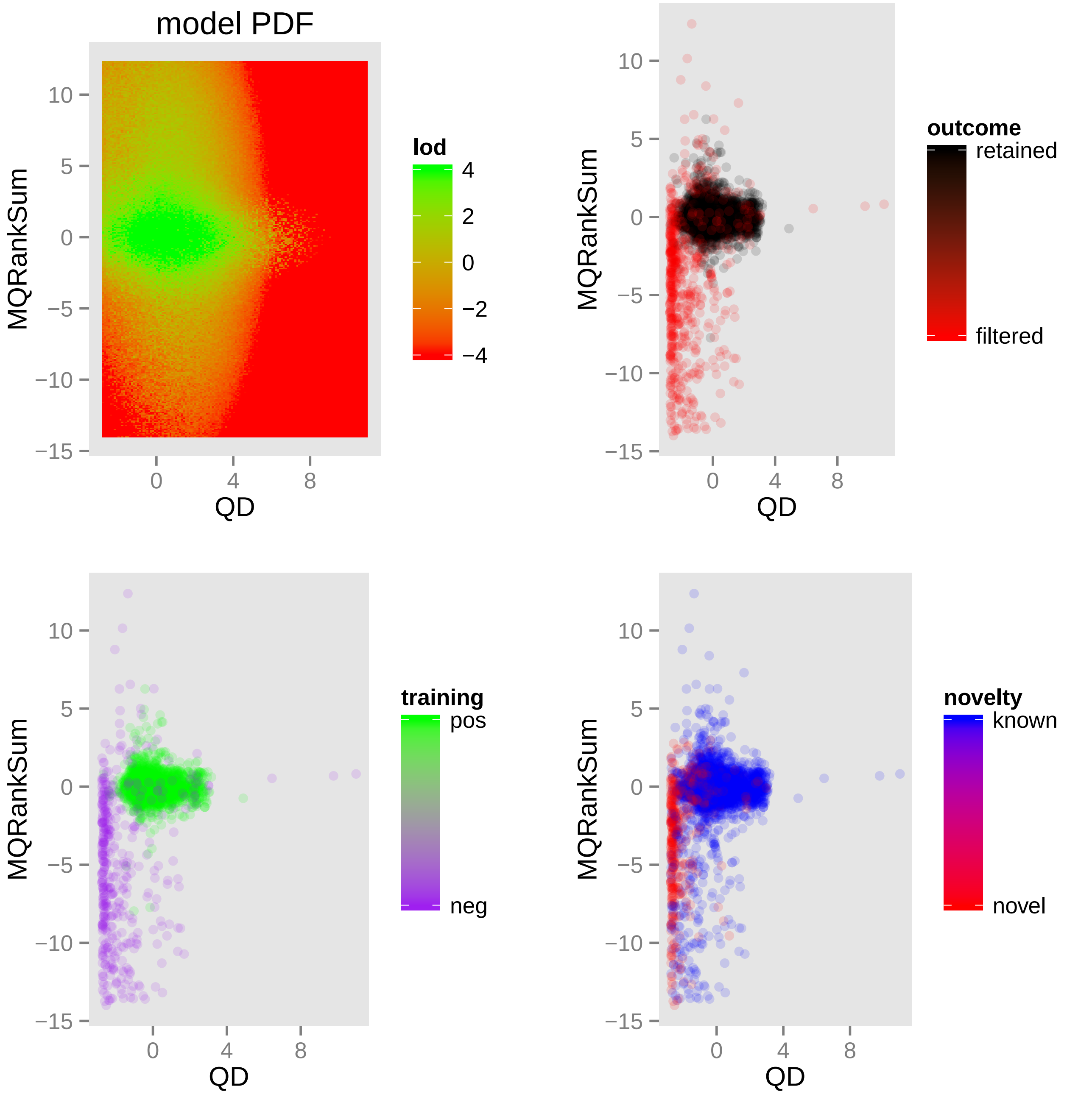
model PDF
MQRankSum
QD
lod
4
2
0
−2
−4
outcome
retained
filtered
training
pos
neg
novelty
known
novel
10
5
0
−5
−10
−15
0
4
8

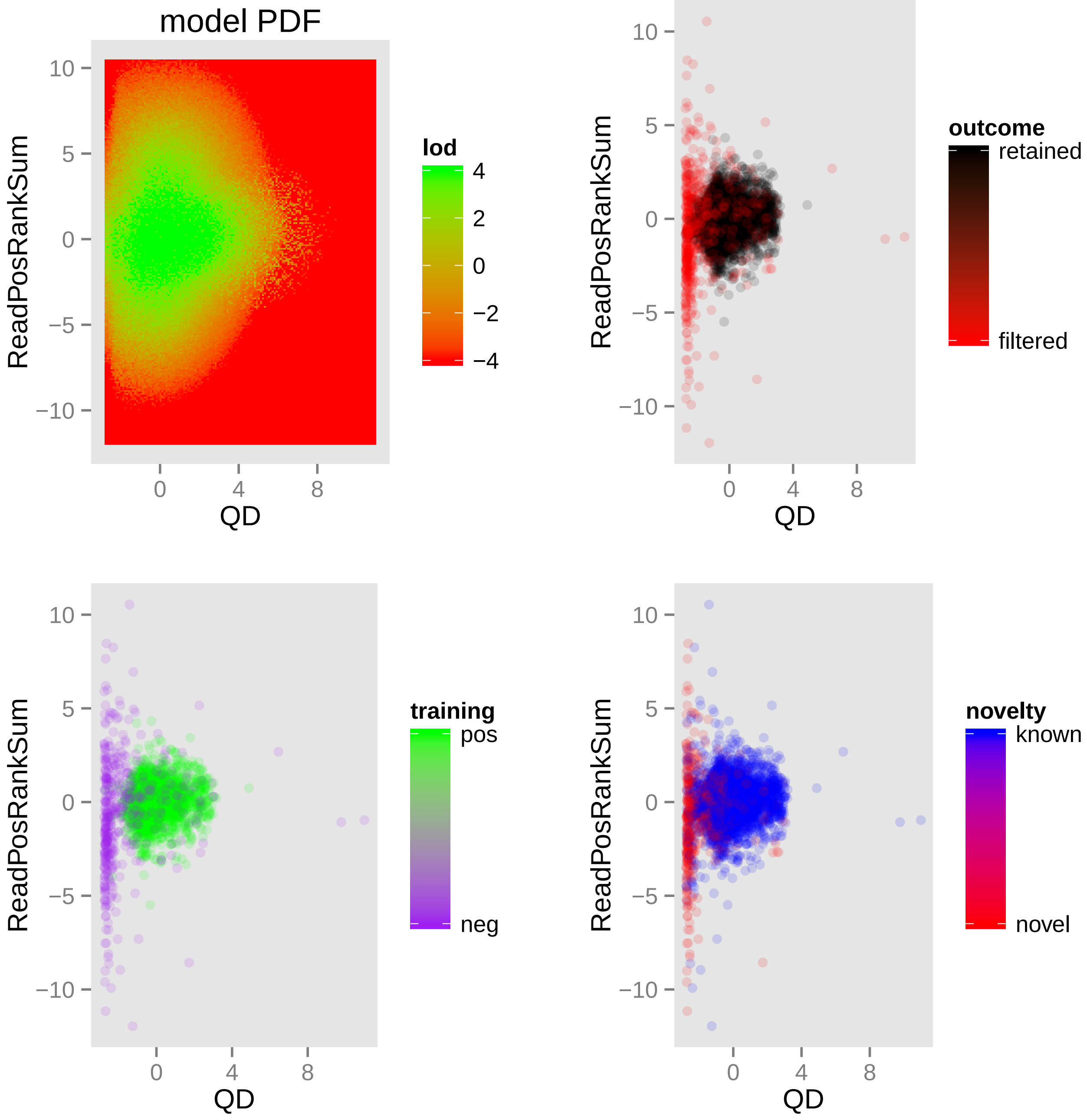
model PDF
lod
4
2
0
−2
−4
ReadPosRankSum
QD
10
5
0
−5
−10
0
4
8
outcome
retained
filtered
training
pos
neg
novelty
known
novel

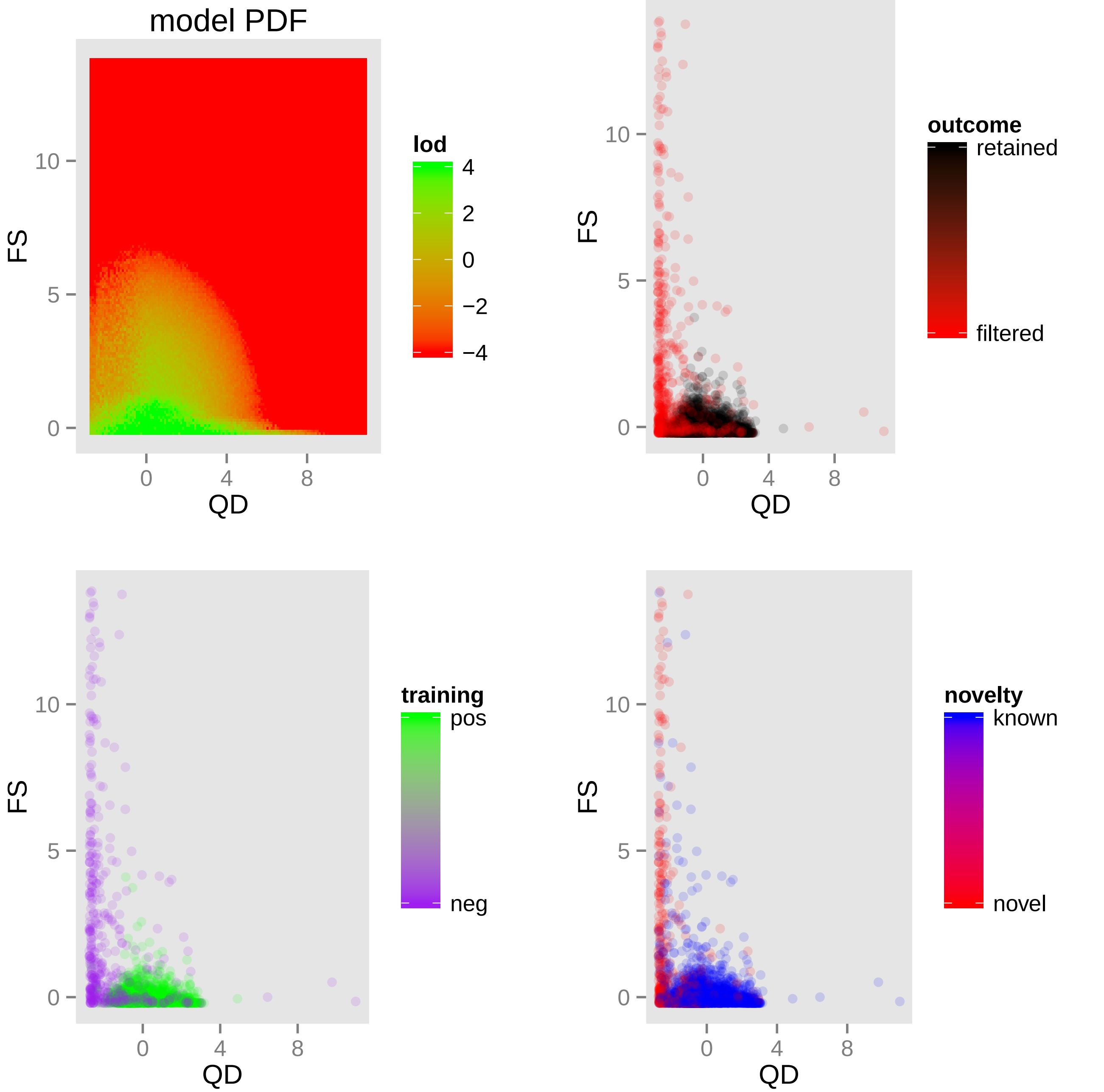

model PDF
lod
4
2
0
−2
−4
10
5
0
FS
0
4
8
QD
outcome
retained
filtered
training
pos
neg
novelty
known
novel

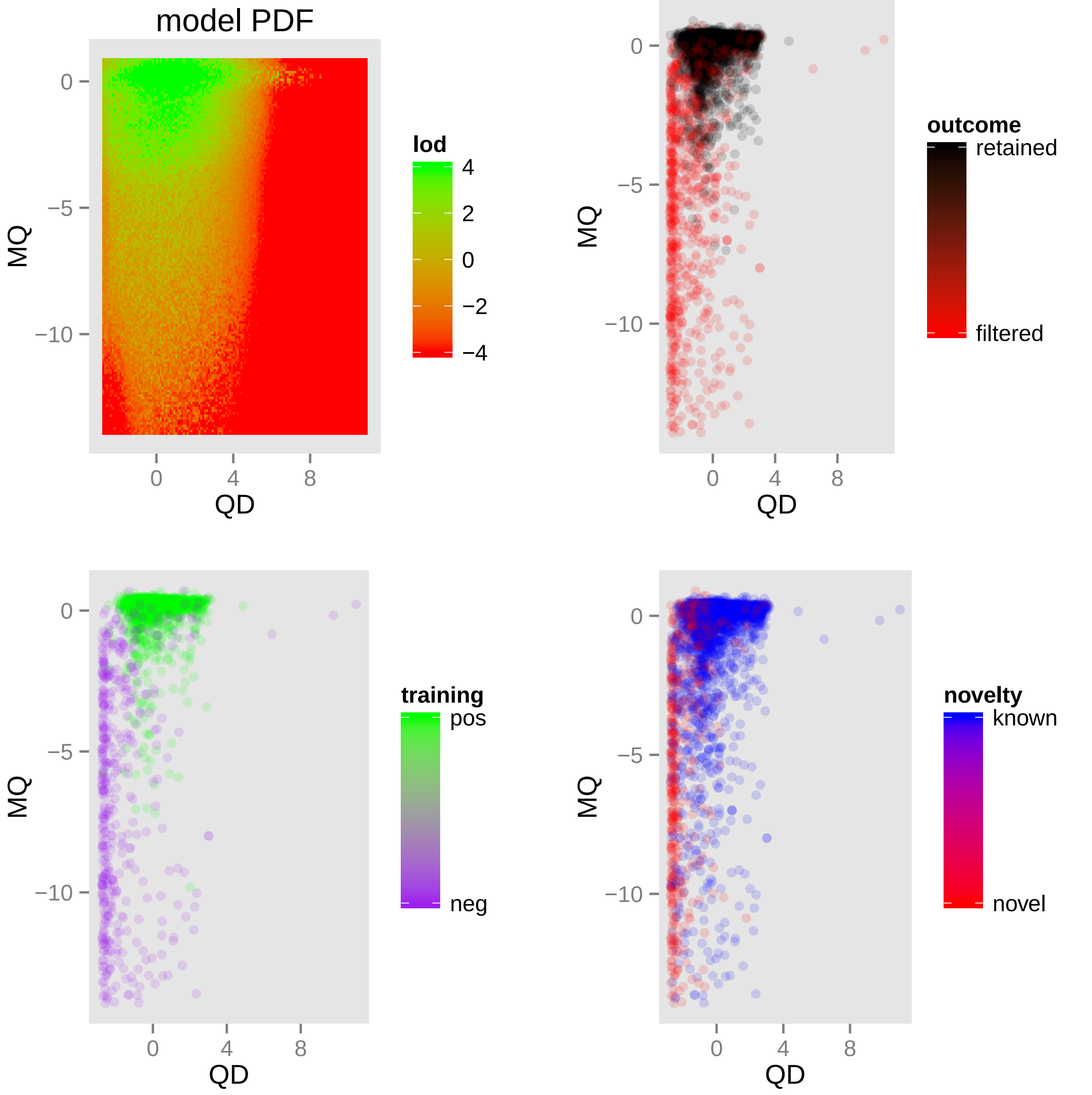

model PDF
MQ
QD
lod
4
2
0
−2
−4
outcome
retained
filtered
training
pos
neg
novelty
known
novel

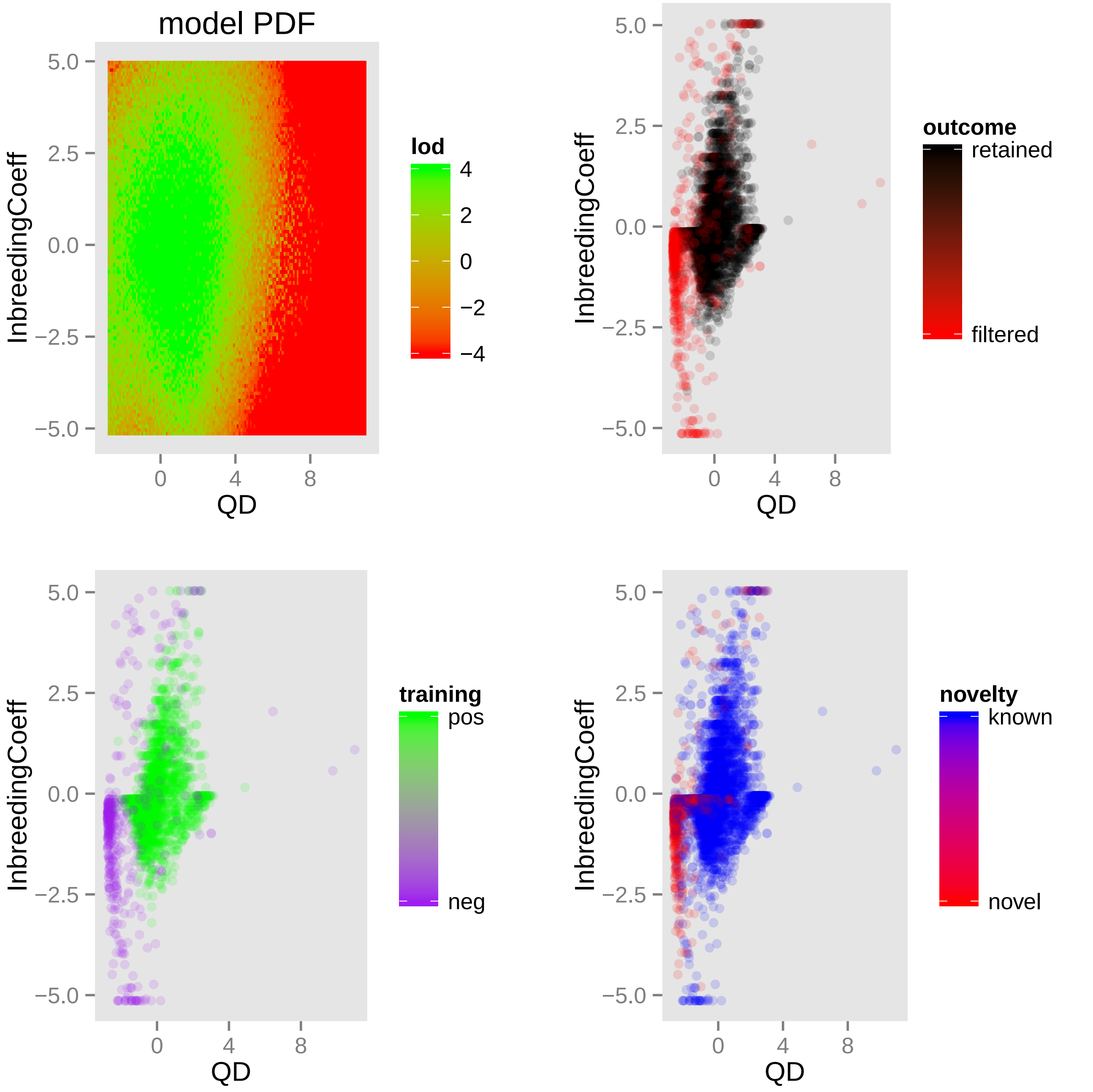
model PDF
InbreedingCoeff
QD
lod
4
2
0
−2
−4
outcome
retained
filtered
training
pos
neg
novelty
known
novel
5.0
2.5
0.0
−2.5
−5.0
0
4
8

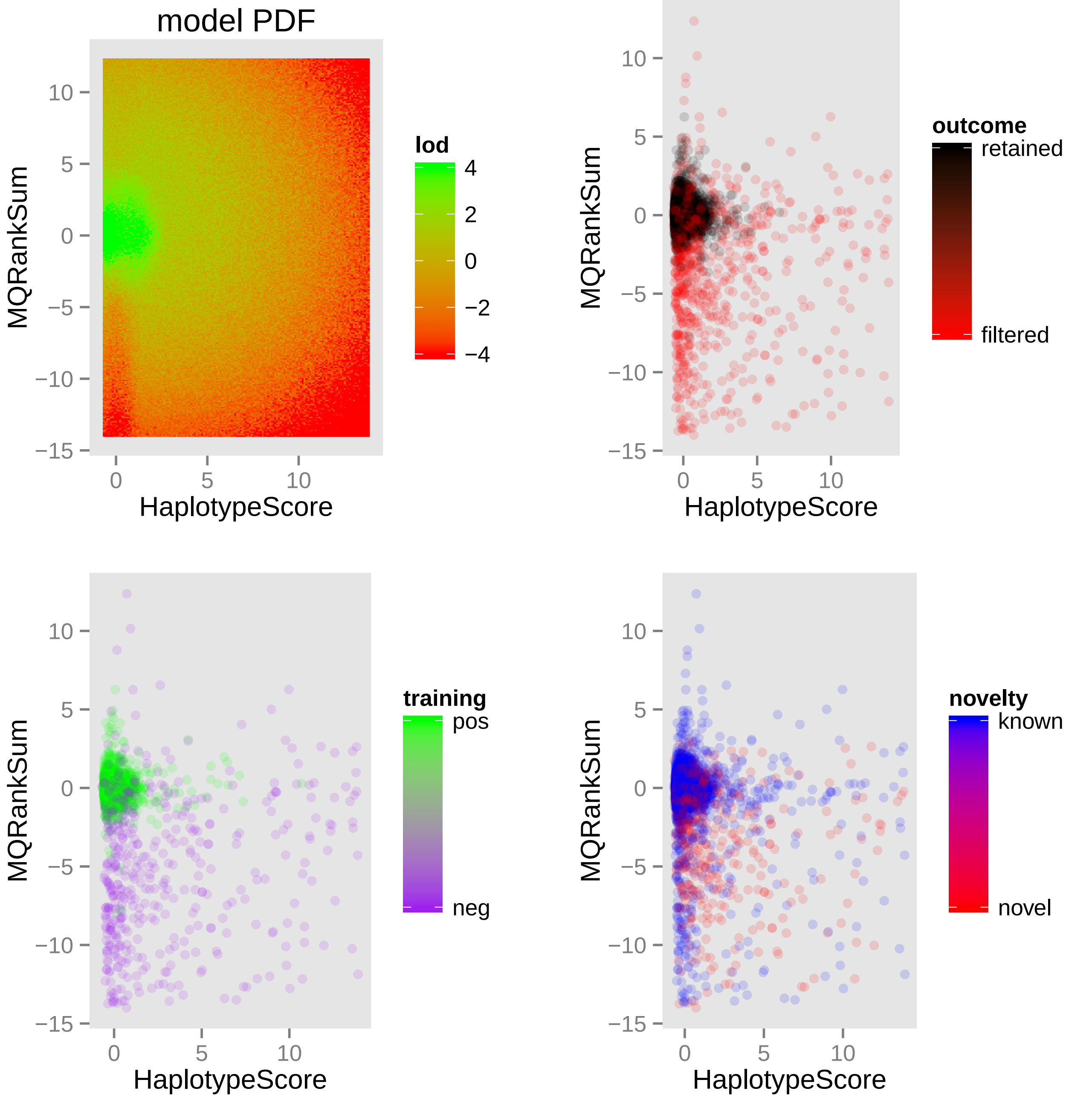
model PDF
MQRankSum
HaplotypeScore
lod
4
2
0
−2
−4
outcome
retained
filtered
training
pos
neg
novelty
known
novel
10
5
0
−5
−10
−15

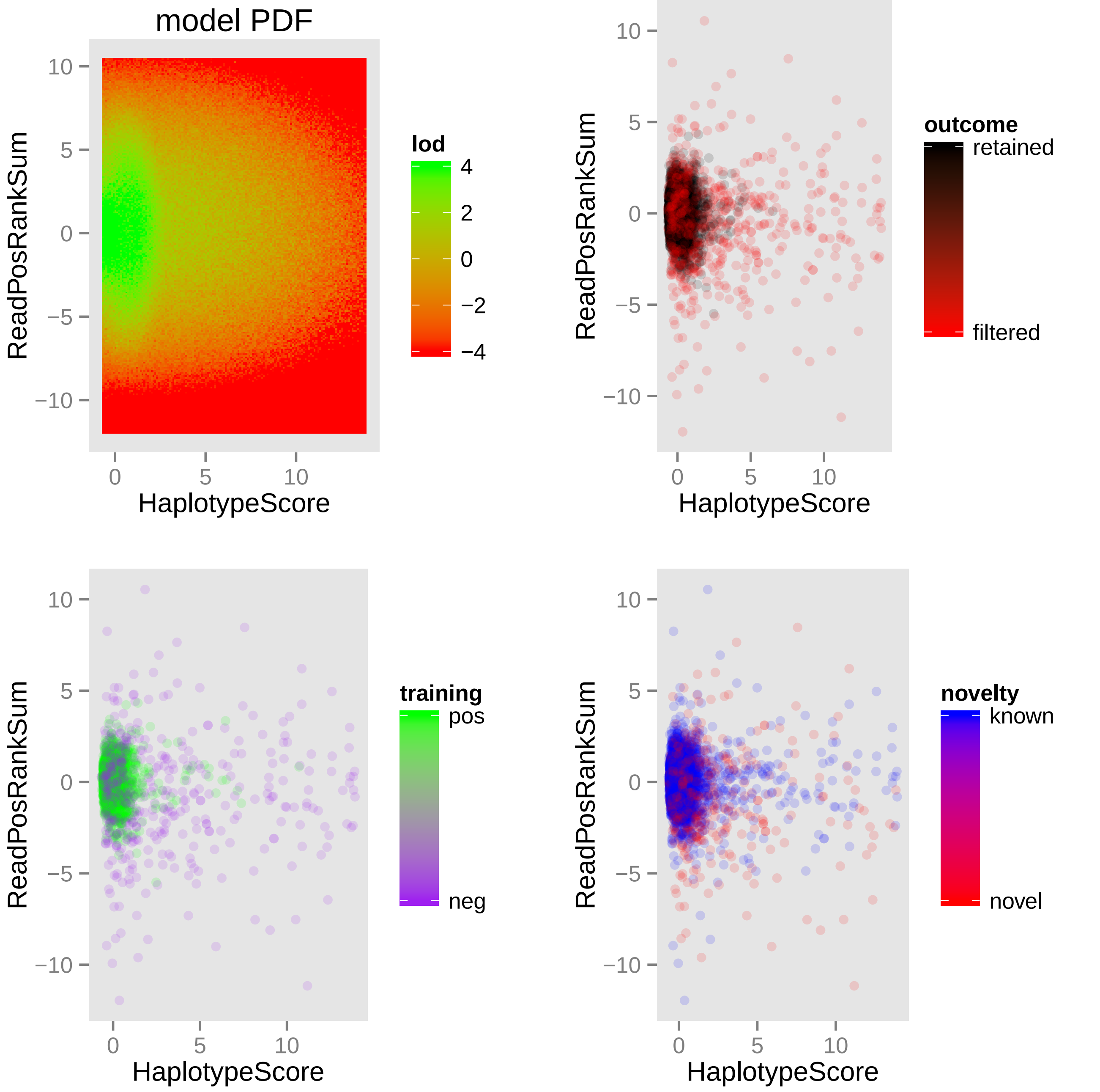

model PDF
lod
4
2
0
−2
−4
outcome
retained
filtered
training
pos
neg
novelty
known
novel
ReadPosRankSum
HaplotypeScore
10
5
0
−5
−10

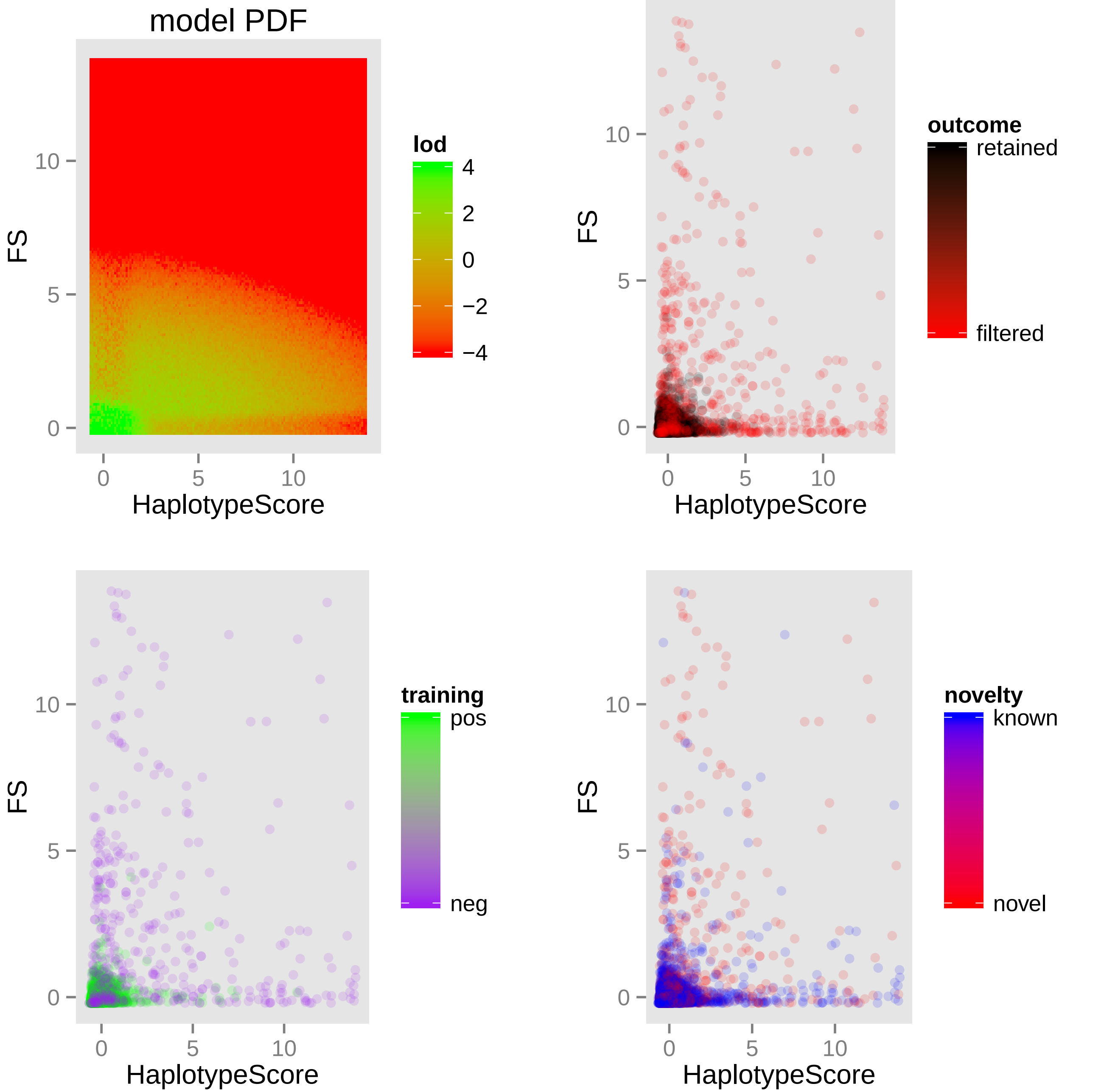
model PDF
lod
4
2
0
−2
−4
FS
HaplotypeScore
outcome
retained
filtered
training
pos
neg
novelty
known
novel

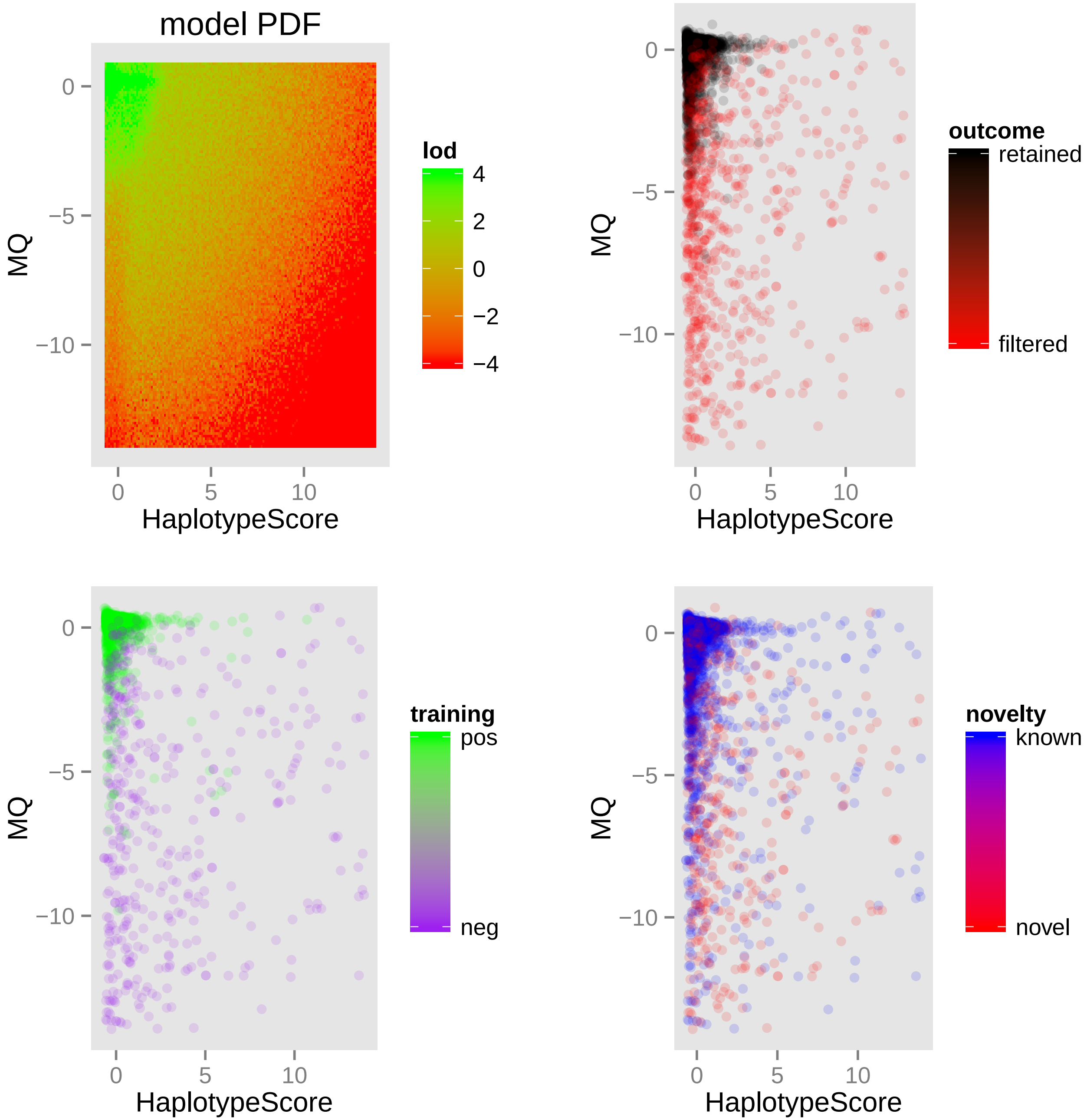

model PDF
MQ
HaplotypeScore
lod
4
2
0
−2
−4
0
−5
−10
5
10
outcome
retained
filtered
training
pos
neg
novelty
known
novel

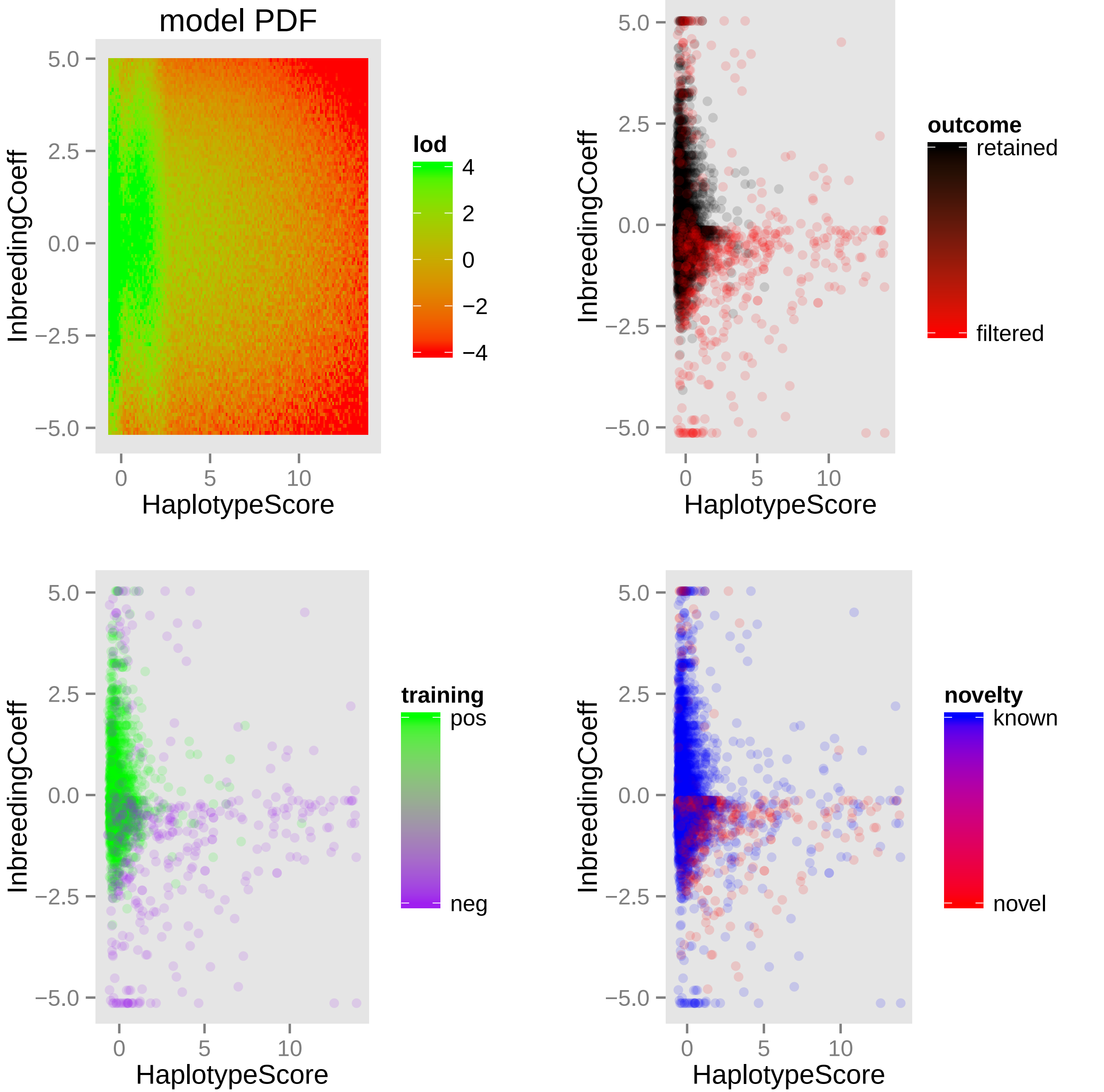

model PDF
InbreedingCoeff
HaplotypeScore
lod
4
2
0
−2
−4
5.0
2.5
0.0
−2.5
−5.0
0
5
10
outcome
retained
filtered
training
pos
neg
novelty
known
novel

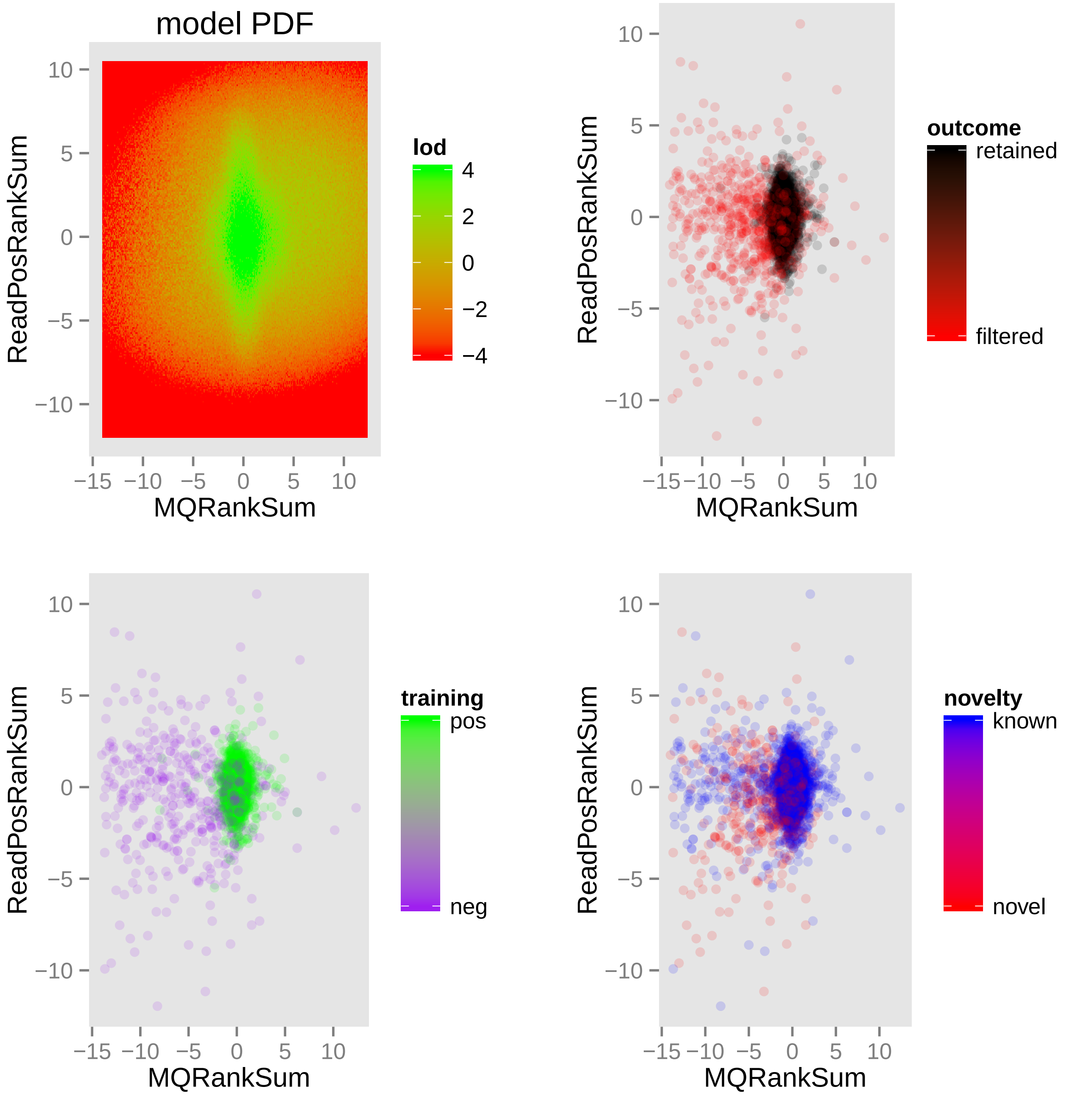
model PDF
lod
4
2
0
−2
−4
ReadPosRankSum
MQRankSum
10
5
0
−5
−10
−15
outcome
retained
filtered
training
pos
neg
novelty
known
novel

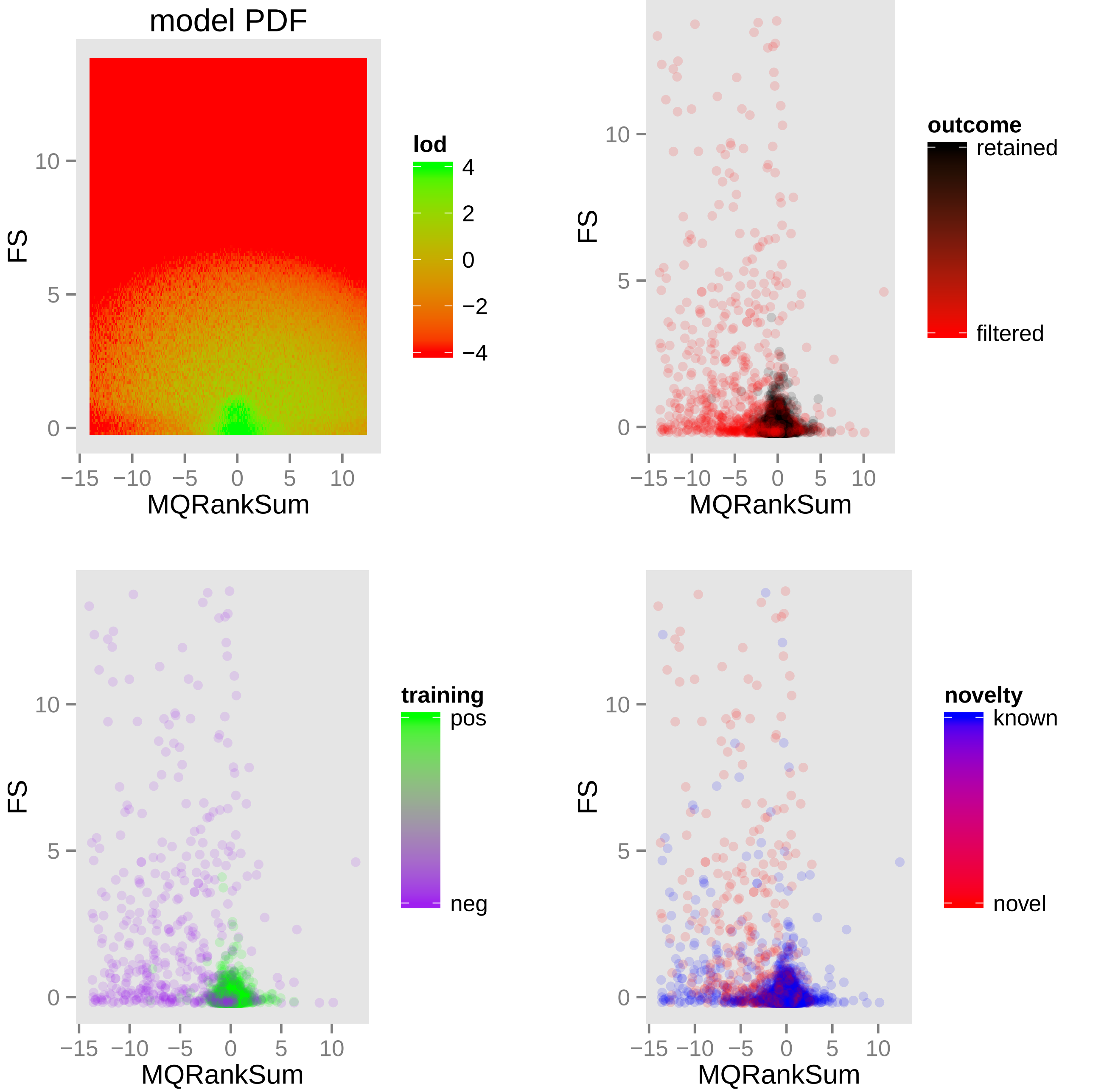
model PDF
FS
MQRankSum
lod
4
2
0
−2
−4
outcome
retained
filtered
training
pos
neg
novelty
known
novel
10
5
0
−15
−10
−5
0
5
10

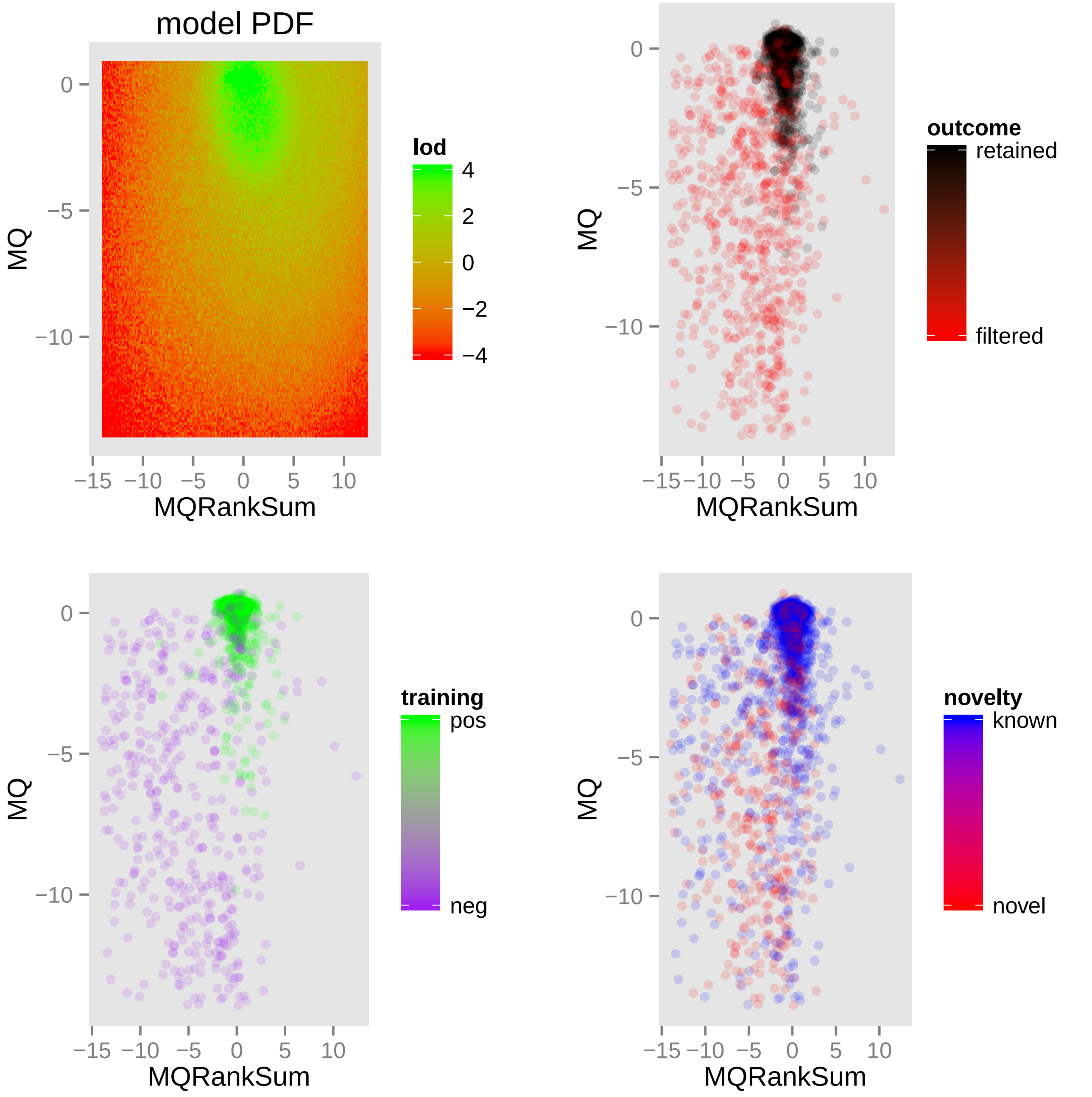
model PDF
MQ
MQRankSum
lod
4
2
0
−2
−4
0
−5
−10
−15
−10
−5
0
5
10
outcome
retained
filtered
training
pos
neg
novelty
known
novel

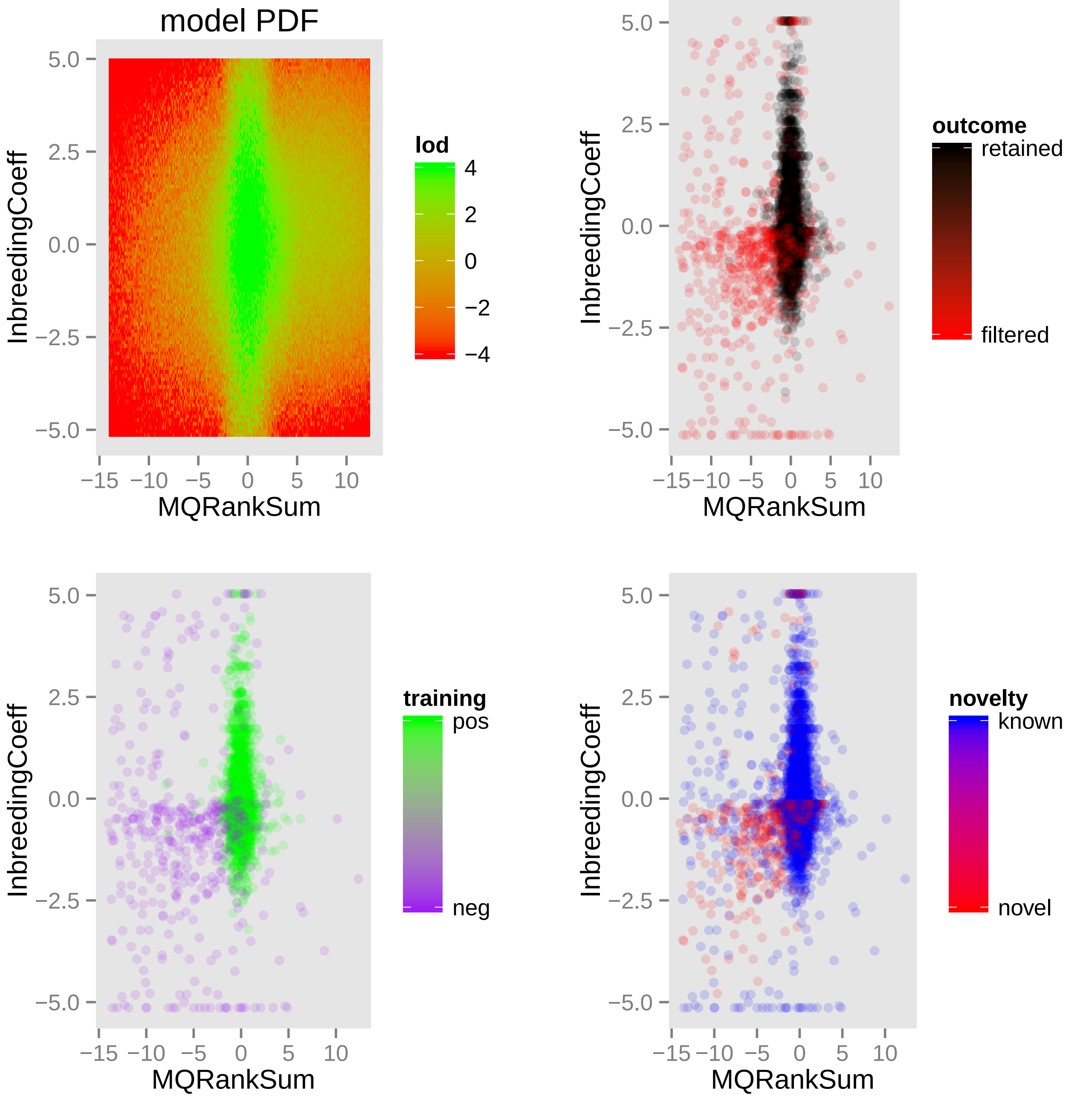
model PDF
lod
4
2
0
−2
−4
InbreedingCoeff
MQRankSum
5.0
2.5
0.0
−2.5
−5.0
−15
−10
−5
0
5
10
outcome
retained
filtered
training
pos
neg
novelty
known
novel

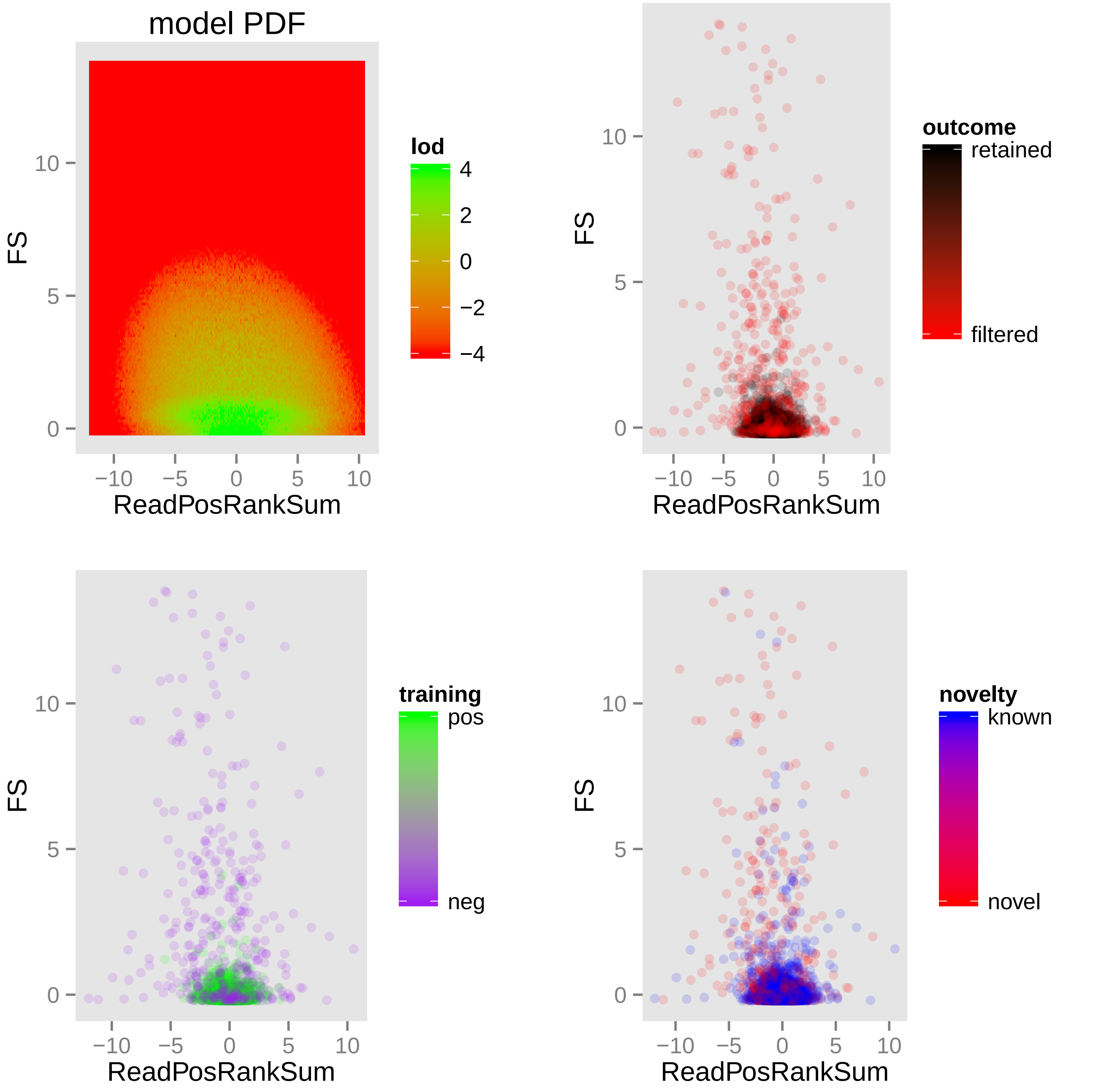
model PDF
lod
4
2
0
−2
−4
FS
ReadPosRankSum
outcome
retained
filtered
training
pos
neg
novelty
known
novel
10
5
0
−10
−5
0
5
10

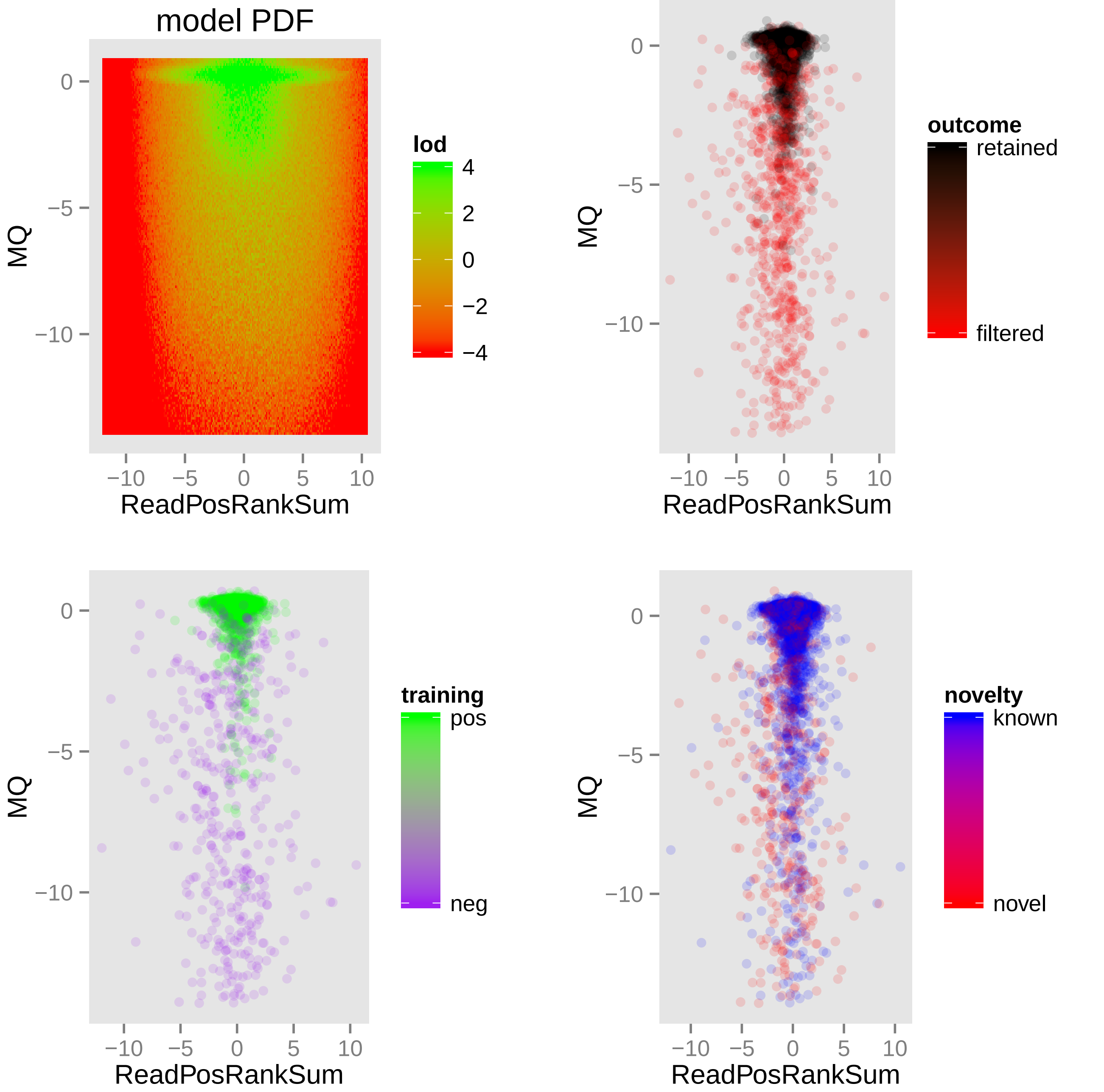
model PDF
lod
4
2
0
−2
−4
MQ
ReadPosRankSum
outcome
retained
filtered
training
pos
neg
novelty
known
novel
0
−5
−10
5
10

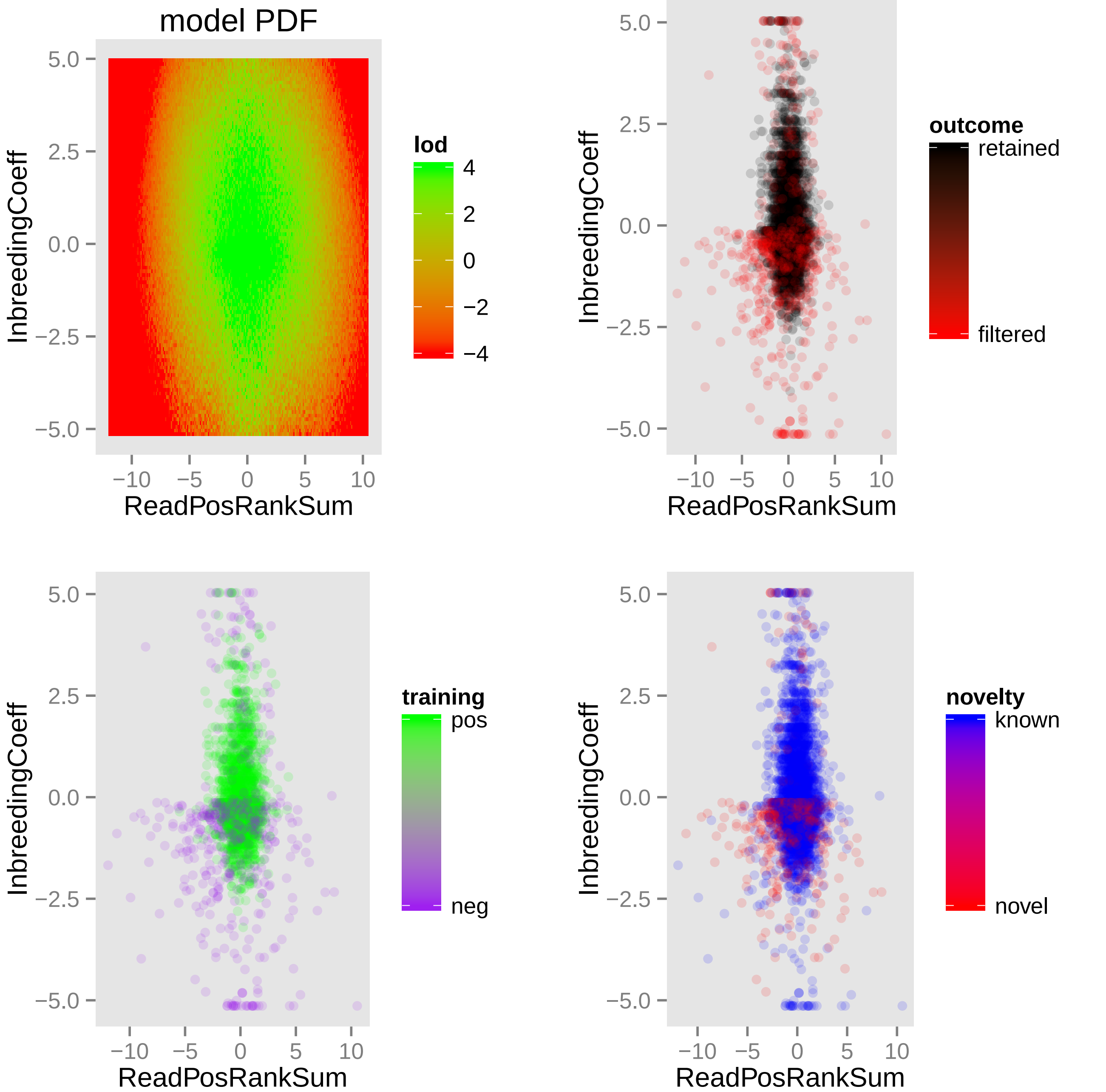

model PDF
lod
4
2
0
−2
−4
InbreedingCoeff
ReadPosRankSum
5.0
2.5
0.0
−2.5
−5.0
−10
−5
0
5
10
outcome
retained
filtered
training
pos
neg
novelty
known
novel

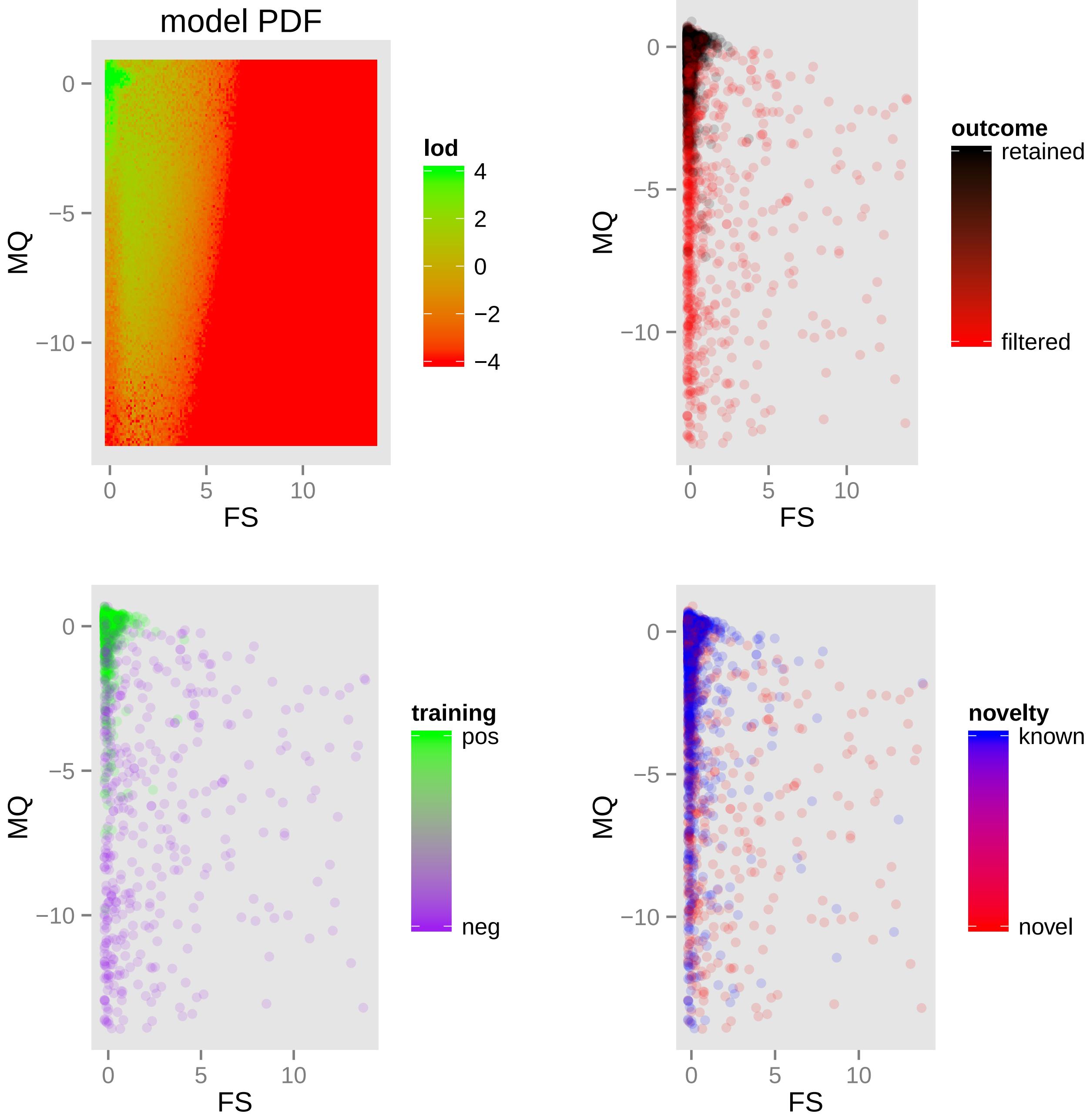

model PDF
MQ
FS
lod
4
2
0
−2
−4
0
−5
−10
0
5
10
outcome
retained
filtered
training
pos
neg
novelty
known
novel

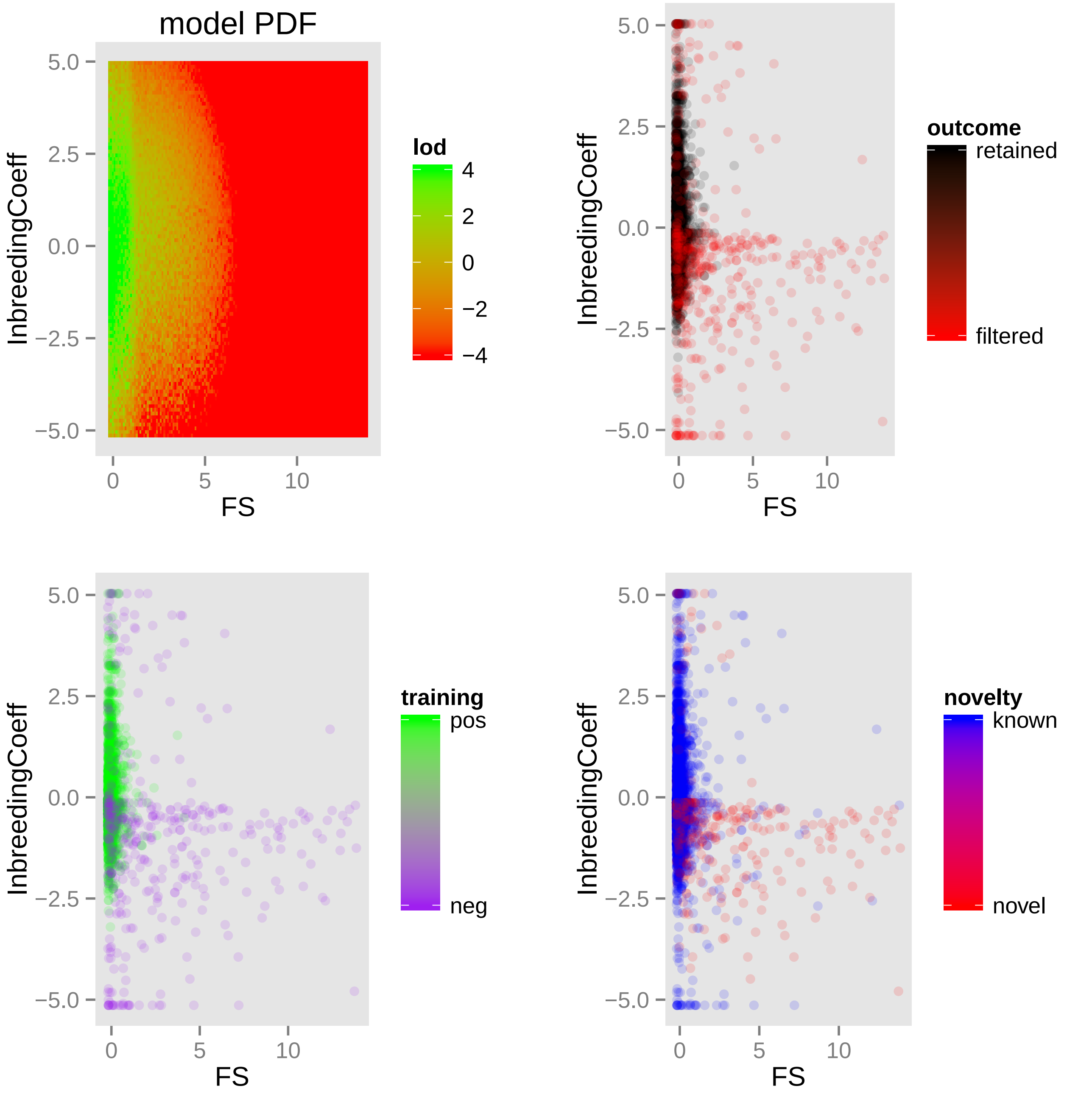
model PDF
lod
4
2
0
−2
−4
InbreedingCoeff
FS
5.0
2.5
0.0
−2.5
−5.0
0
5
10
outcome
retained
filtered
training
pos
neg
novelty
known
novel

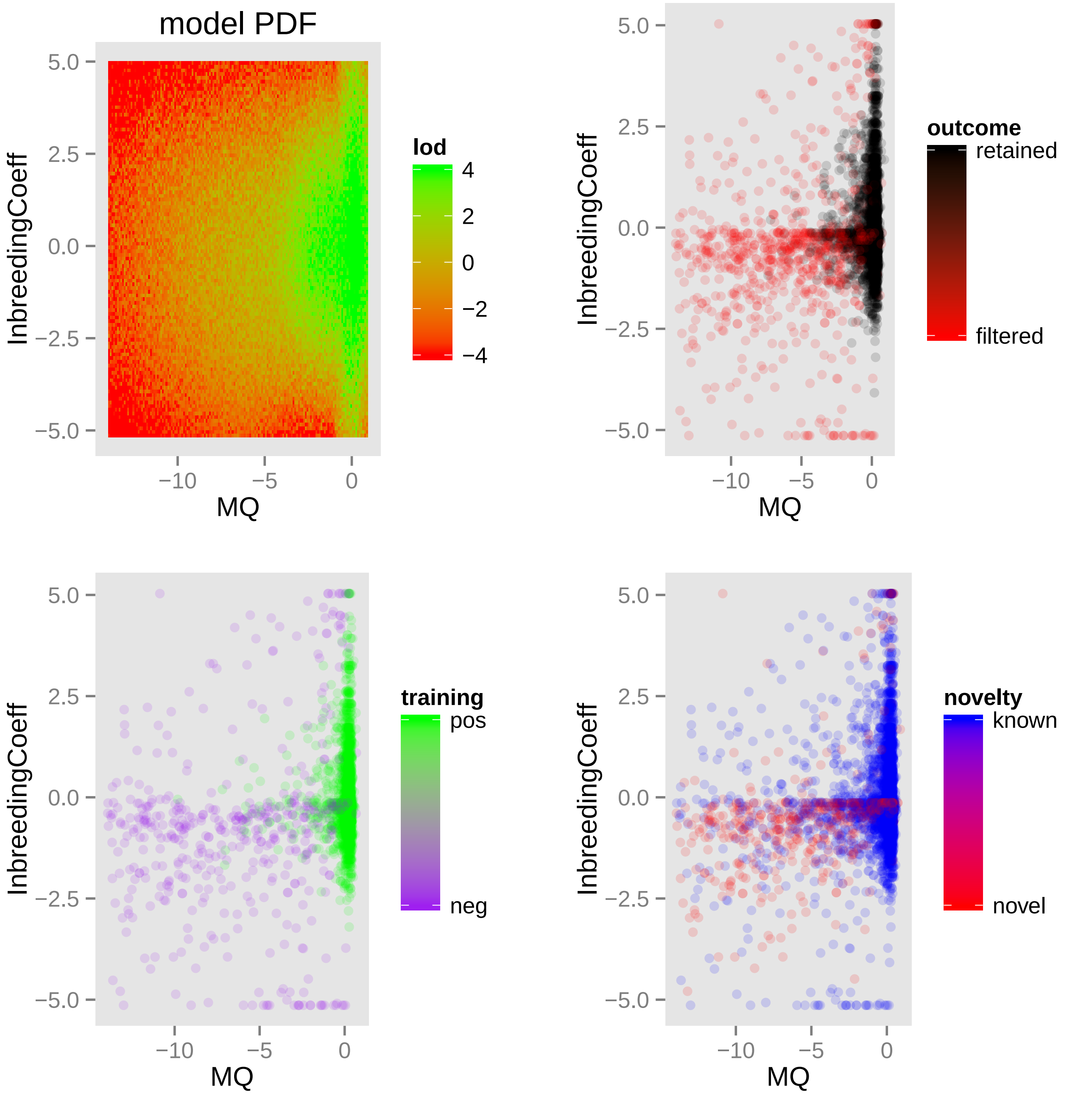
model PDF
lod
4
2
0
−2
−4
outcome
retained
filtered
training
pos
neg
novelty
known
novel
InbreedingCoeff
5.0
2.5
0.0
−2.5
−5.0
MQ
−10
−5
0

UnifiedGenotyper INDELs VQSR

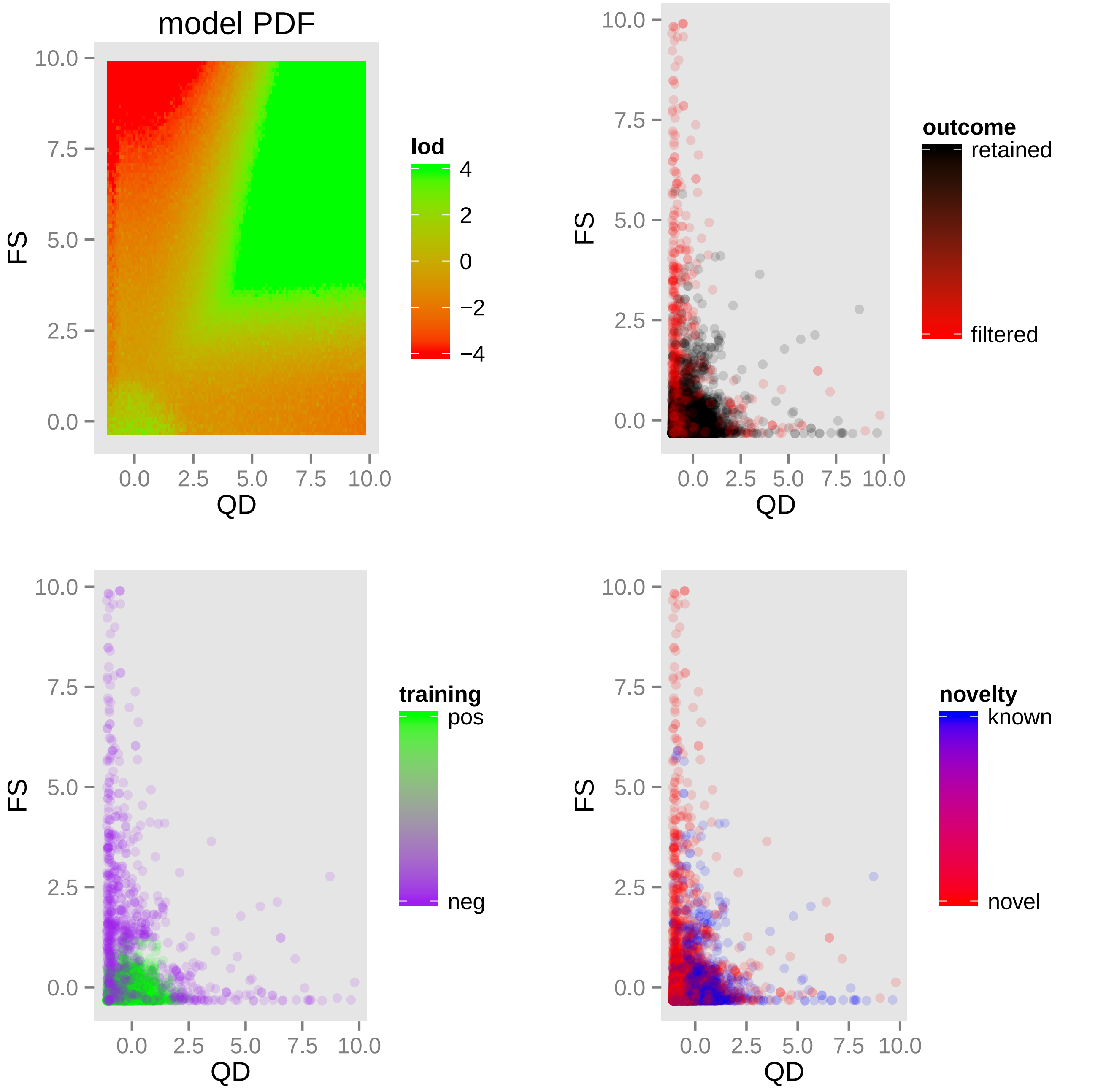

model PDF
lod
4
2
0
−2
−4
FS
QD
0.0
2.5
5.0
7.5
10.0
outcome
retained
filtered
training
pos
neg
novelty
known
novel

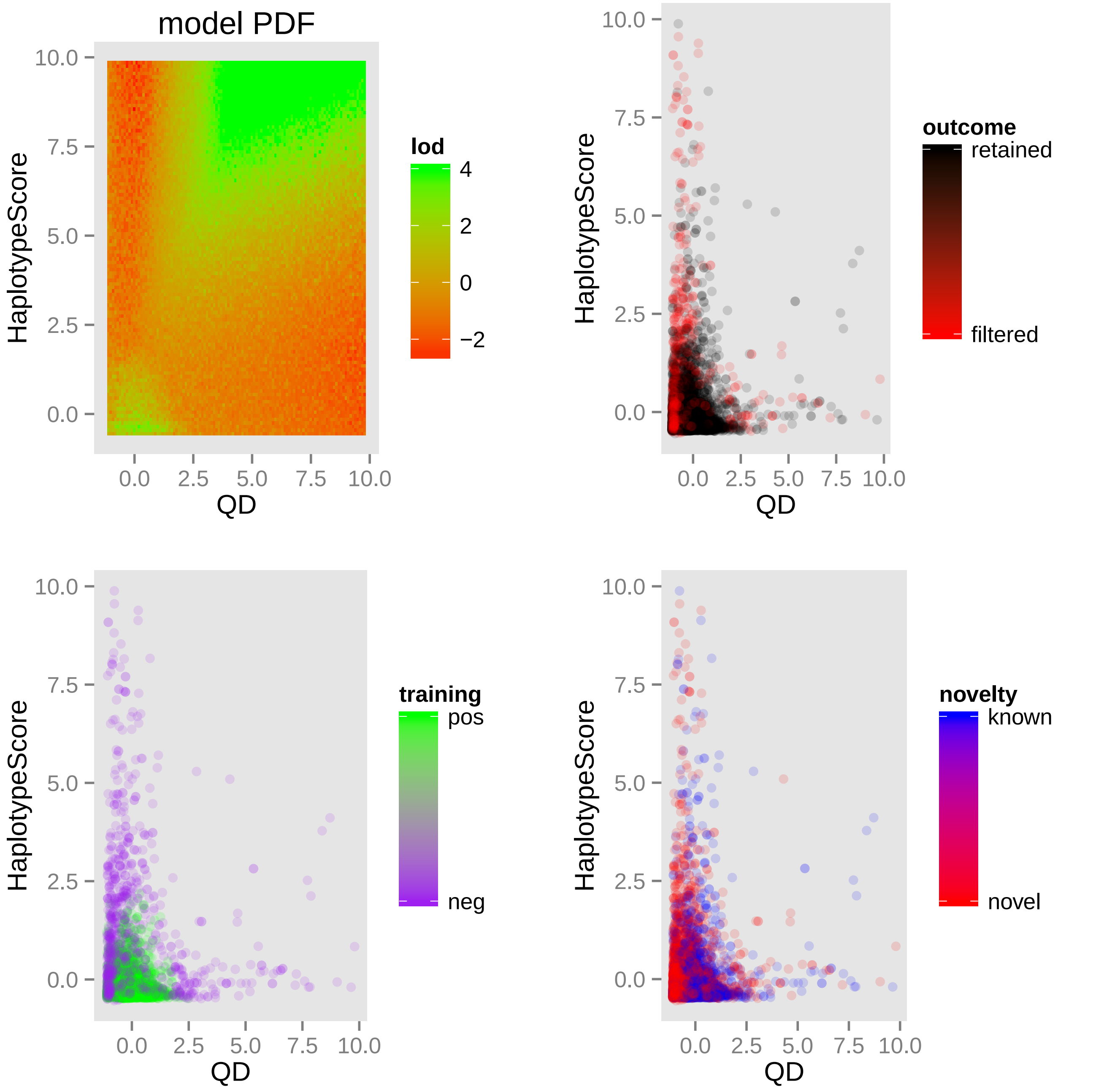
model PDF
HaplotypeScore
QD
lod
4
2
0
−2
outcome
retained
filtered
training
pos
neg
novelty
known
novel
0.0
2.5
5.0
7.5
10.0

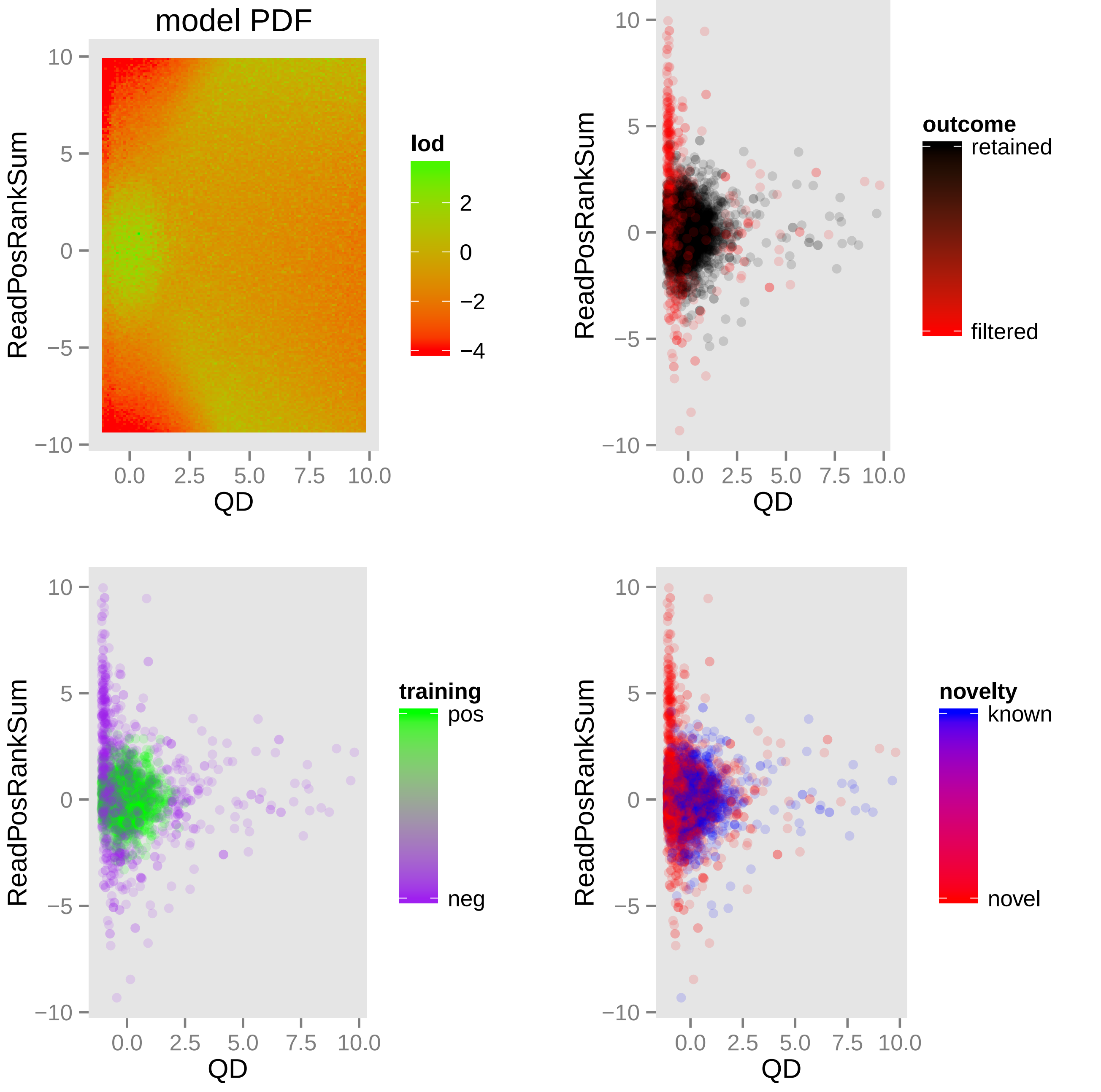

model PDF
lod
2
0
−2
−4
ReadPosRankSum
QD
10
5
0
−5
−10
0.0
2.5
5.0
7.5
10.0
outcome
retained
filtered
training
pos
neg
novelty
known
novel

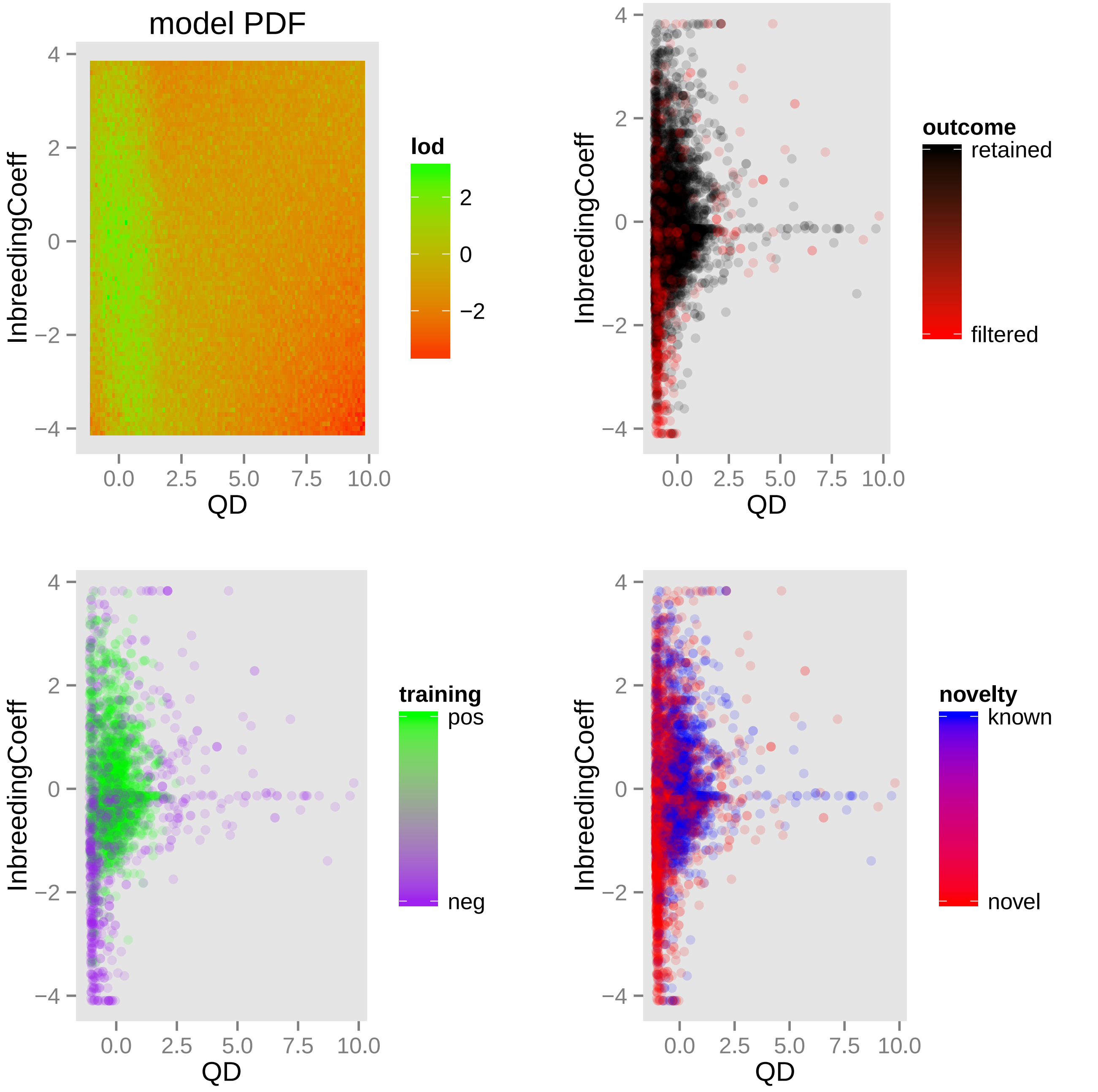

model PDF
lod
retained
filtered
outcome
training
pos
neg
novelty
known
novel
InbreedingCoeff
QD

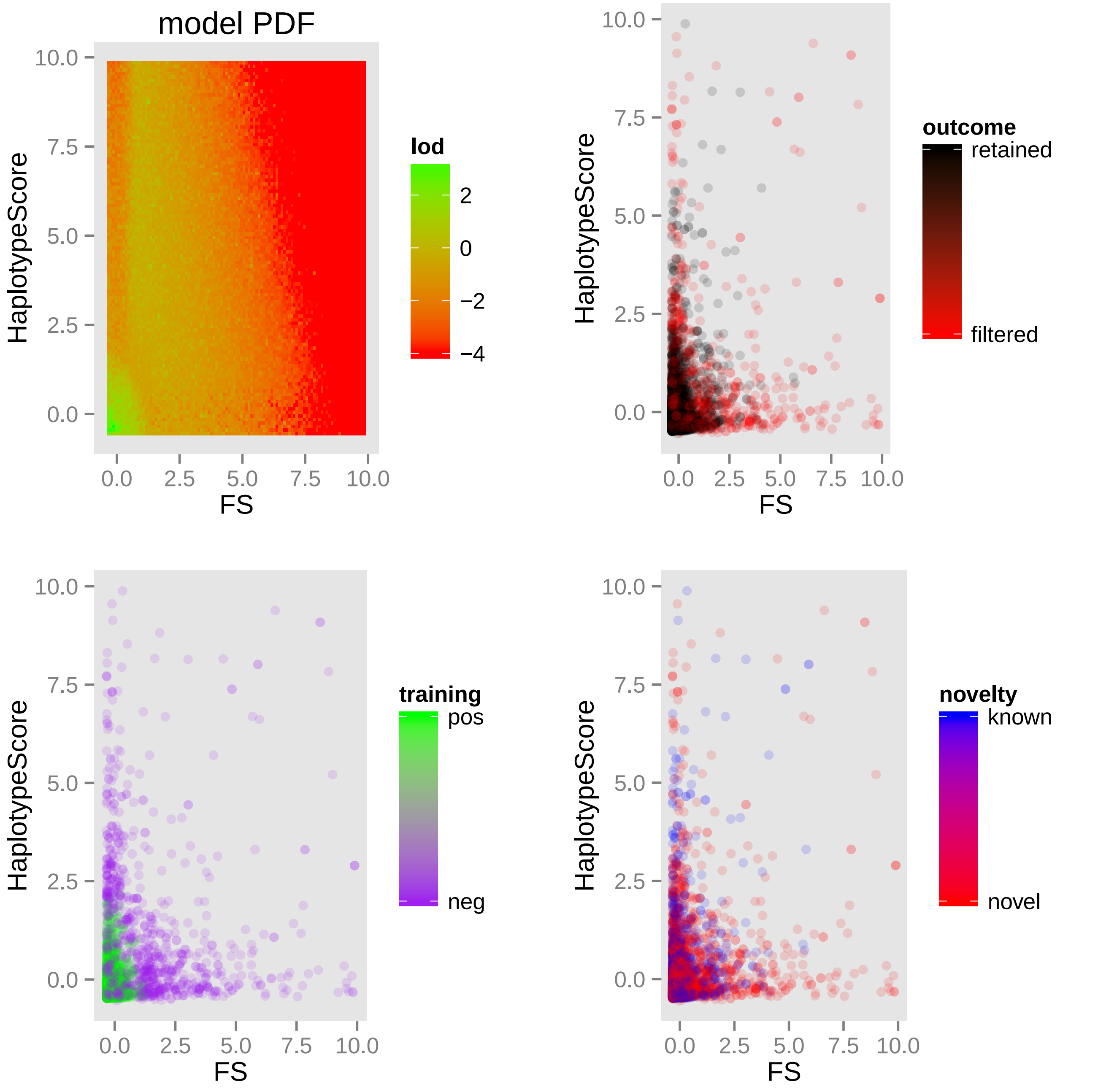
model PDF
lod
2
0
−2
−4
HaplotypeScore
FS
0.0
2.5
5.0
7.5
10.0
outcome
retained
filtered
training
pos
neg
novelty
known
novel

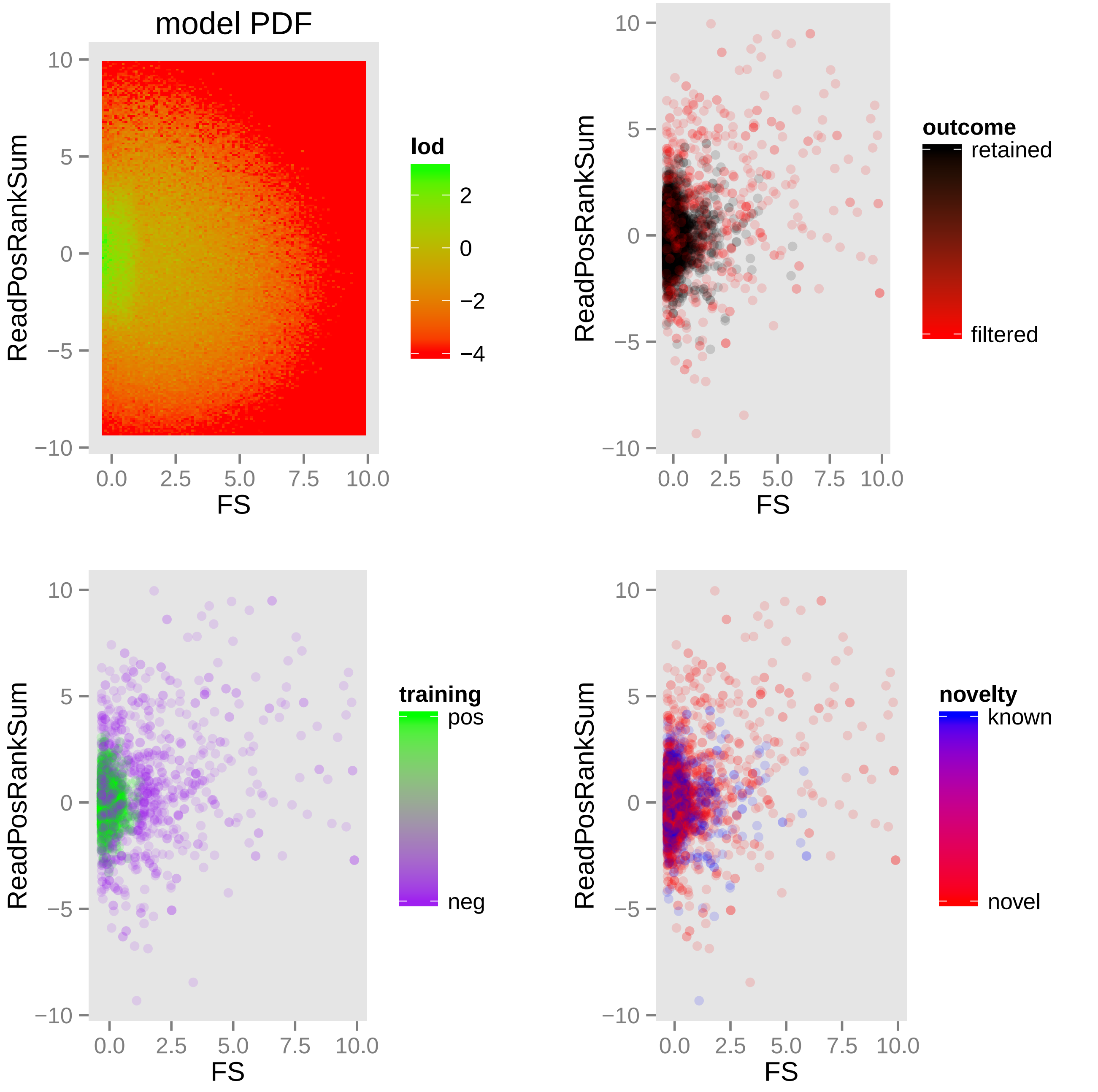
model PDF
lod
2
0
−2
−4
ReadPosRankSum
FS
outcome
retained
filtered
training
pos
neg
novelty
known
novel
10
5
0
−5
−10
0.0
2.5
5.0
7.5
10.0

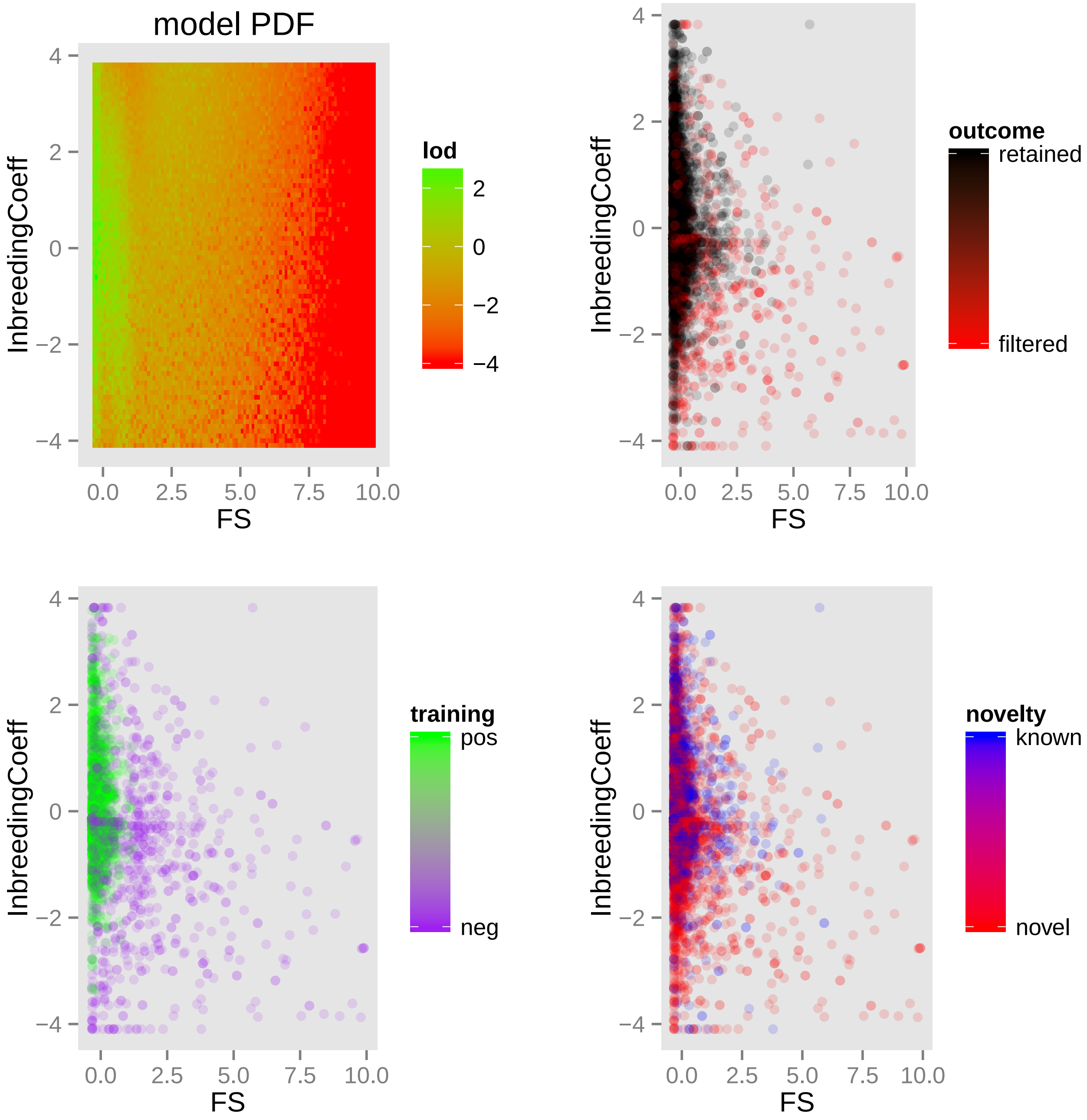
model PDF
lod
2
0
−2
−4
InbreedingCoeff
FS
0.0
2.5
5.0
7.5
10.0
outcome
retained
filtered
training
pos
neg
novelty
known
novel

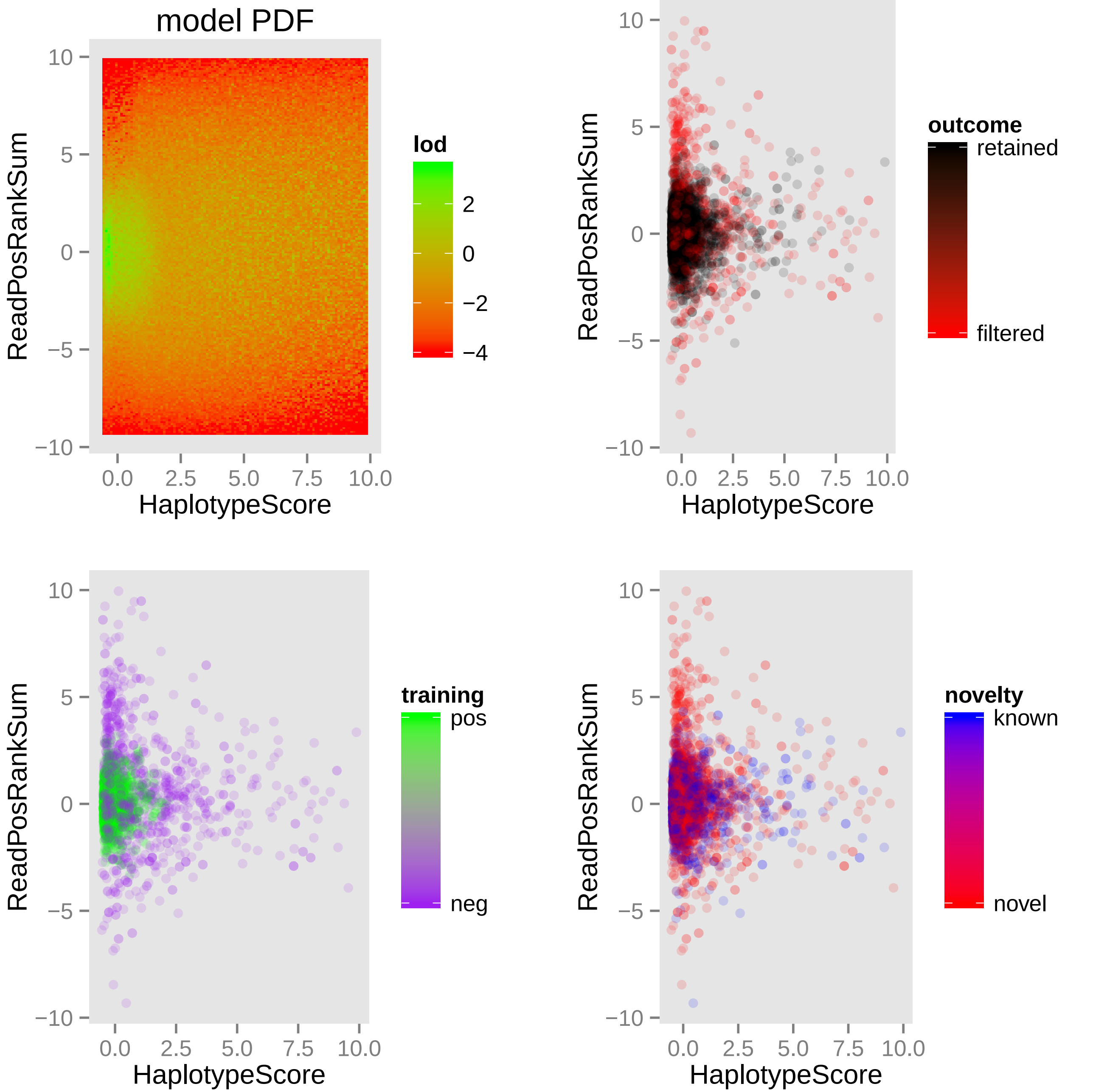
model PDF
lod
2
0
−2
−4
outcome
retained
filtered
training
pos
neg
novelty
known
novel
ReadPosRankSum
HaplotypeScore
10
5
0
−5
−10
0.0
2.5
5.0
7.5
10.0

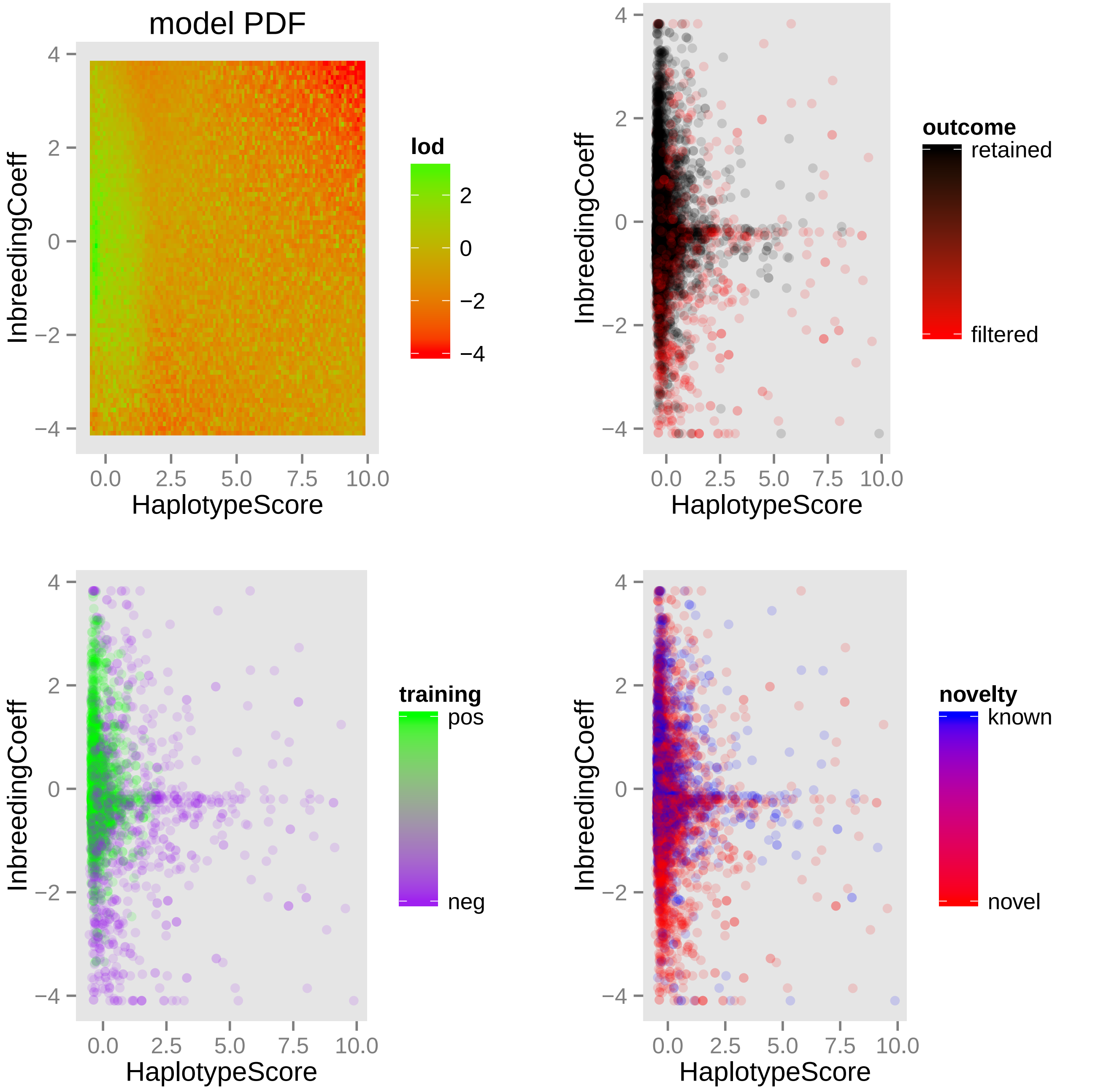
model PDF
InbreedingCoeff
HaplotypeScore
lod
2
0
−2
−4
4
2
0
−2
−4
0.0
2.5
5.0
7.5
10.0
outcome
retained
filtered
training
pos
neg
novelty
known
novel

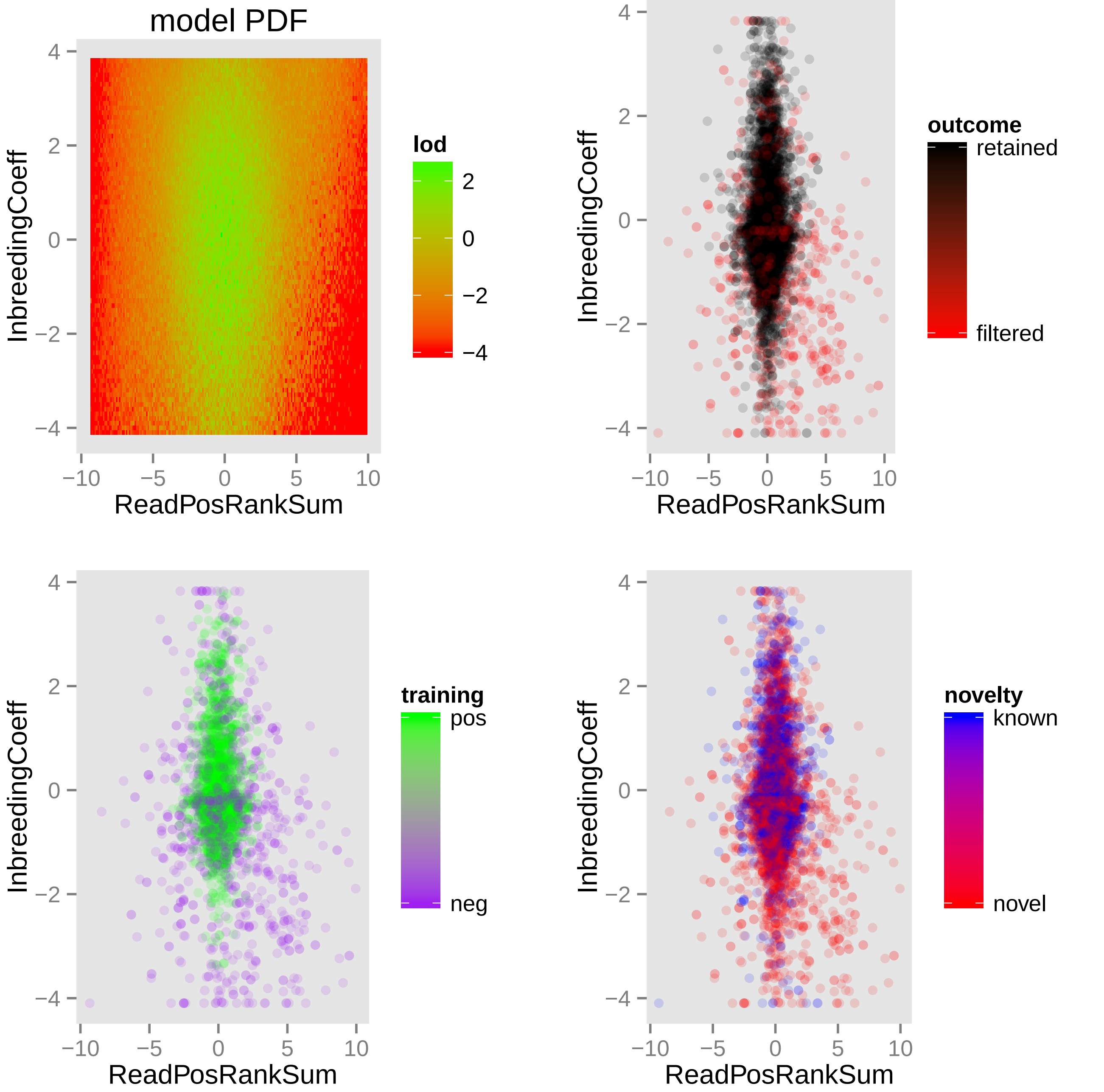
model PDF
lod
2
0
−2
−4
outcome
retained
filtered
training
pos
neg
novelty
known
novel
InbreedingCoeff
ReadPosRankSum
4
2
0
−2
−4
−10
−5
0
5
10

HaplotypeCaller 2.2.2 VQSR

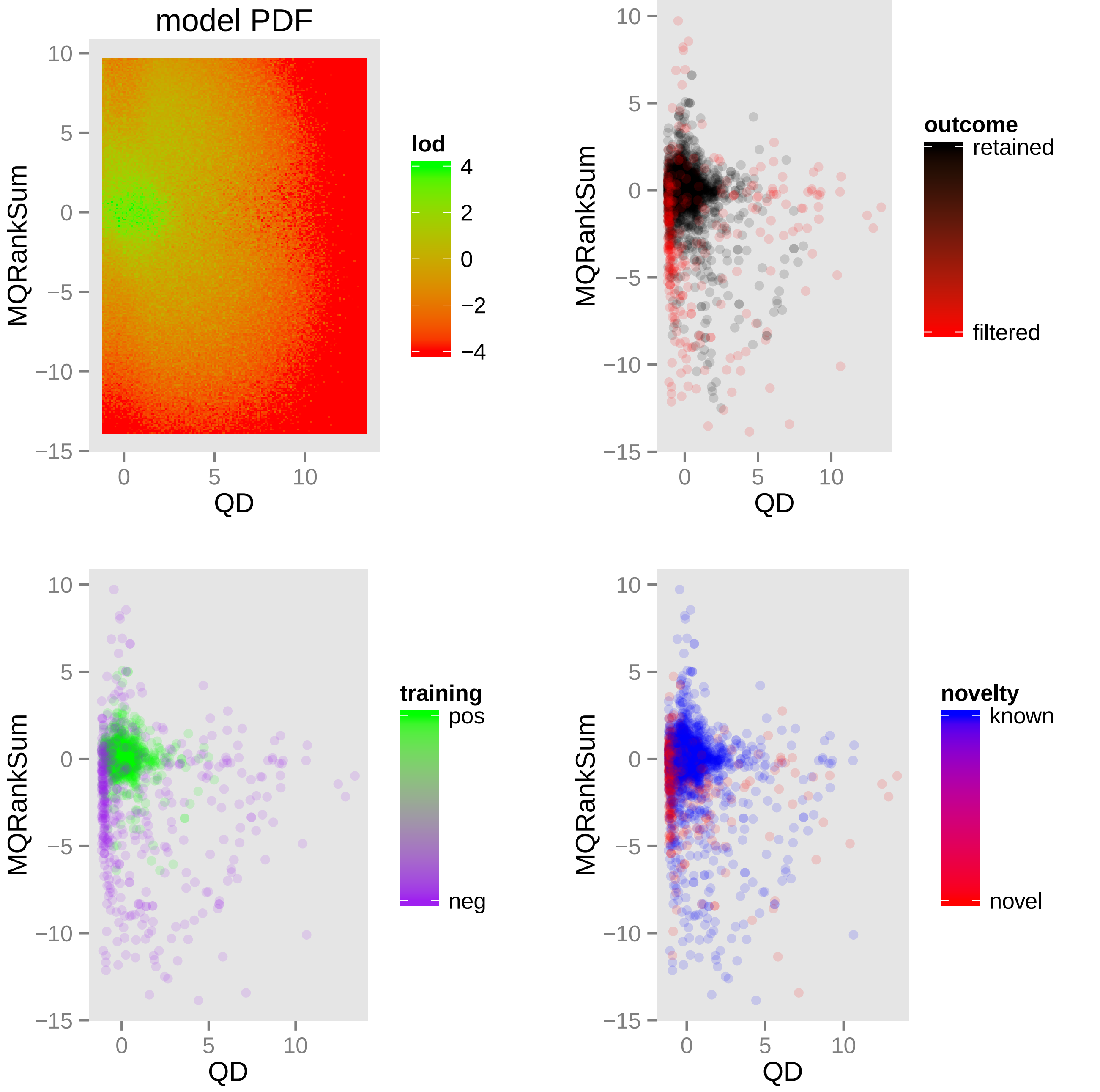
model PDF
lod
4
2
0
−2
−4
outcome
retained
filtered
training
pos
neg
novelty
known
novel
MQRankSum
QD
10
5
0
−5
−10
−15

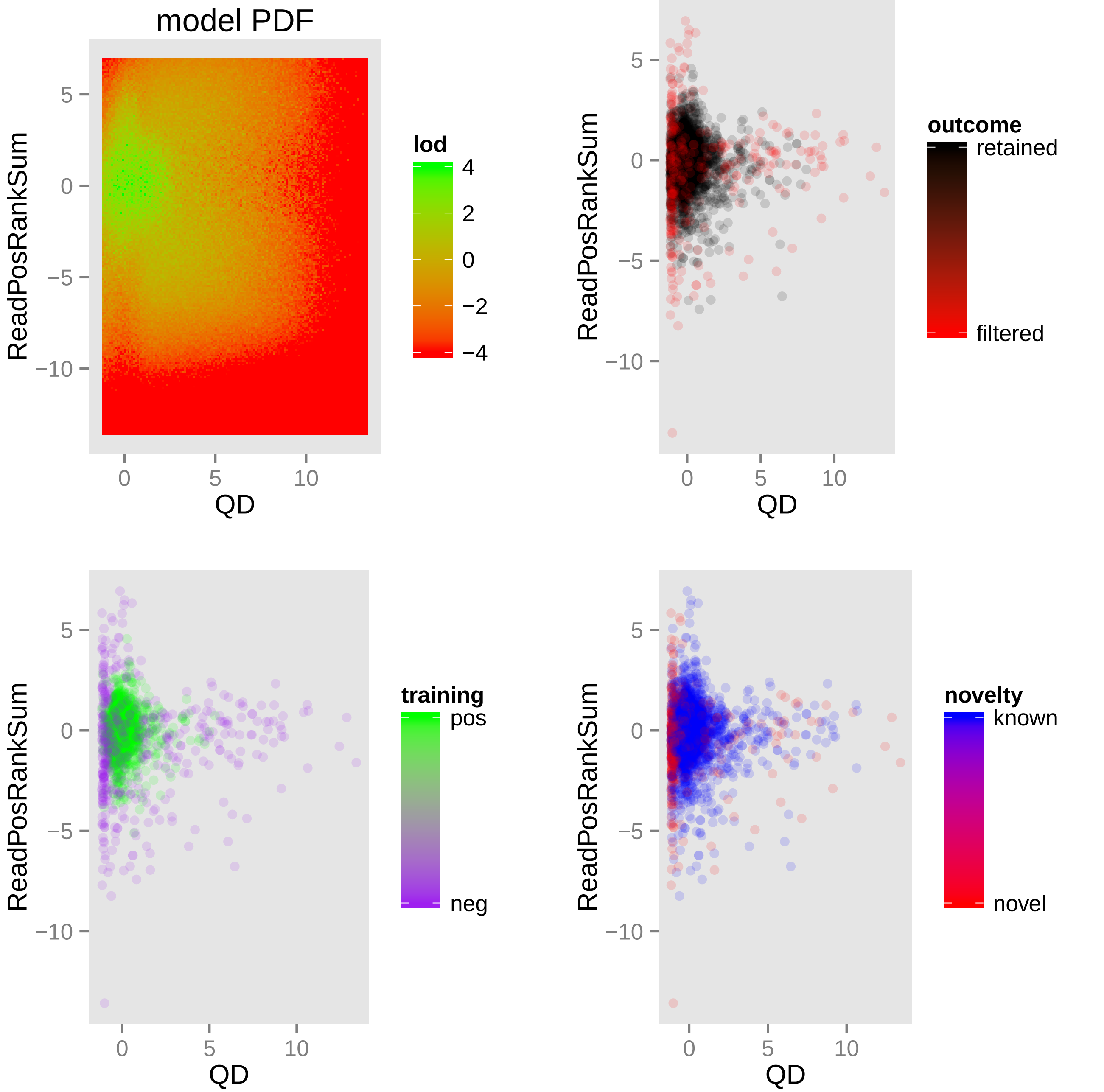

model PDF
lod
4
2
0
−2
−4
ReadPosRankSum
QD
outcome
retained
filtered
training
pos
neg
novelty
known
novel

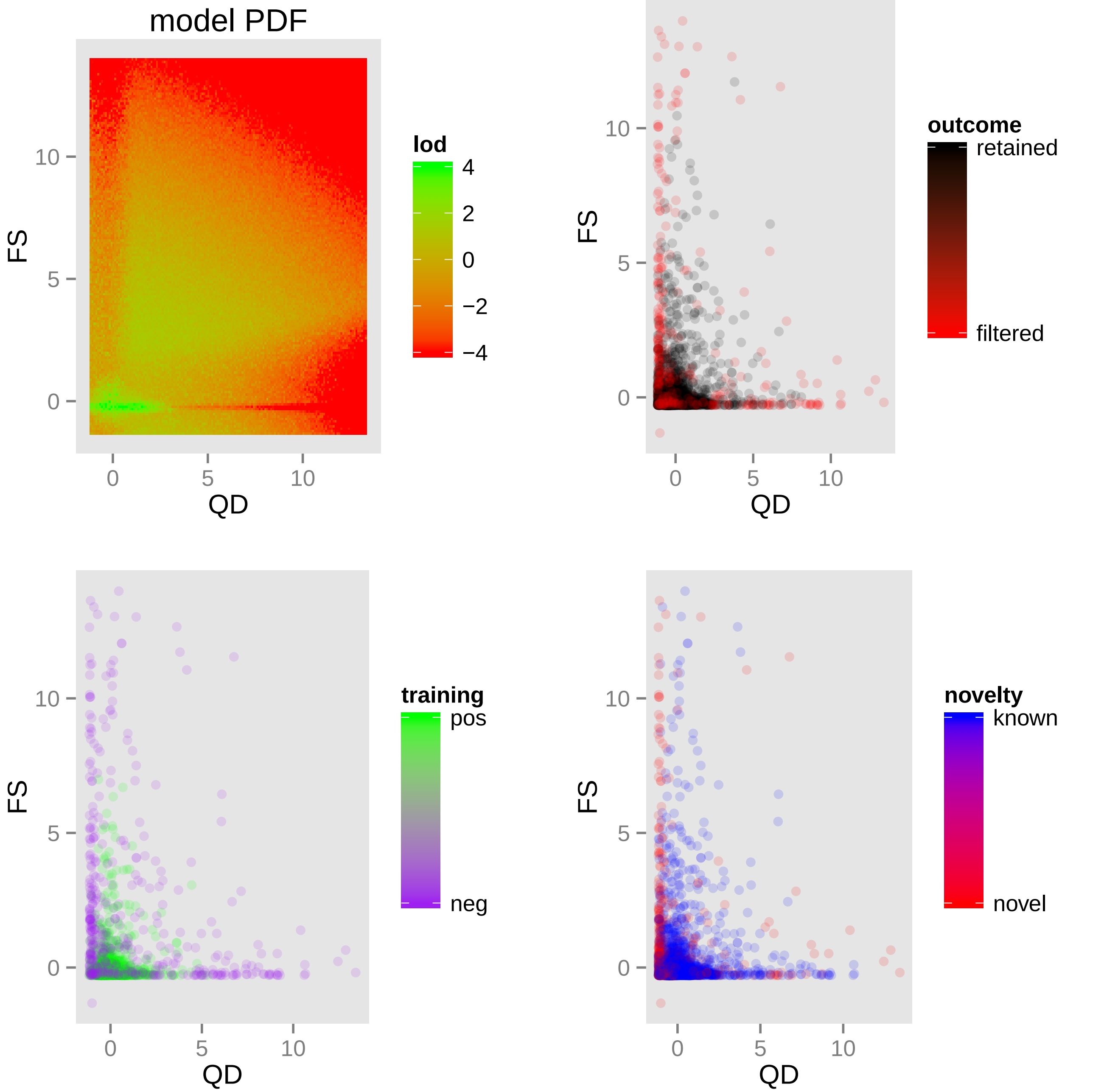

model PDF
FS
QD
lod
4
2
0
−2
−4
outcome
retained
filtered
training
pos
neg
novelty
known
novel

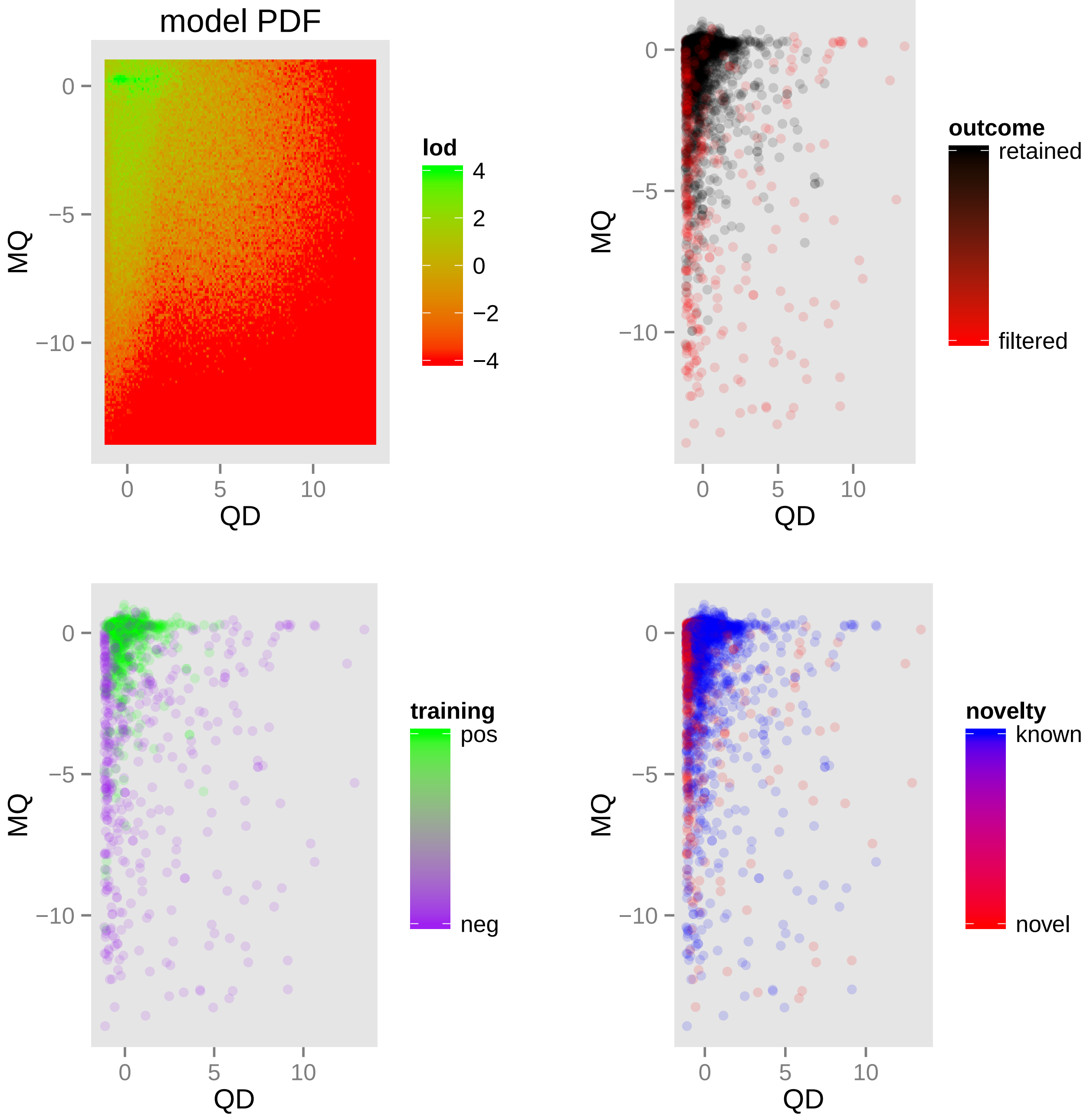

model PDF
MQ
QD
lod
4
2
0
−2
−4
0
−5
−10
0
5
10
outcome
retained
filtered
training
pos
neg
novelty
known
novel

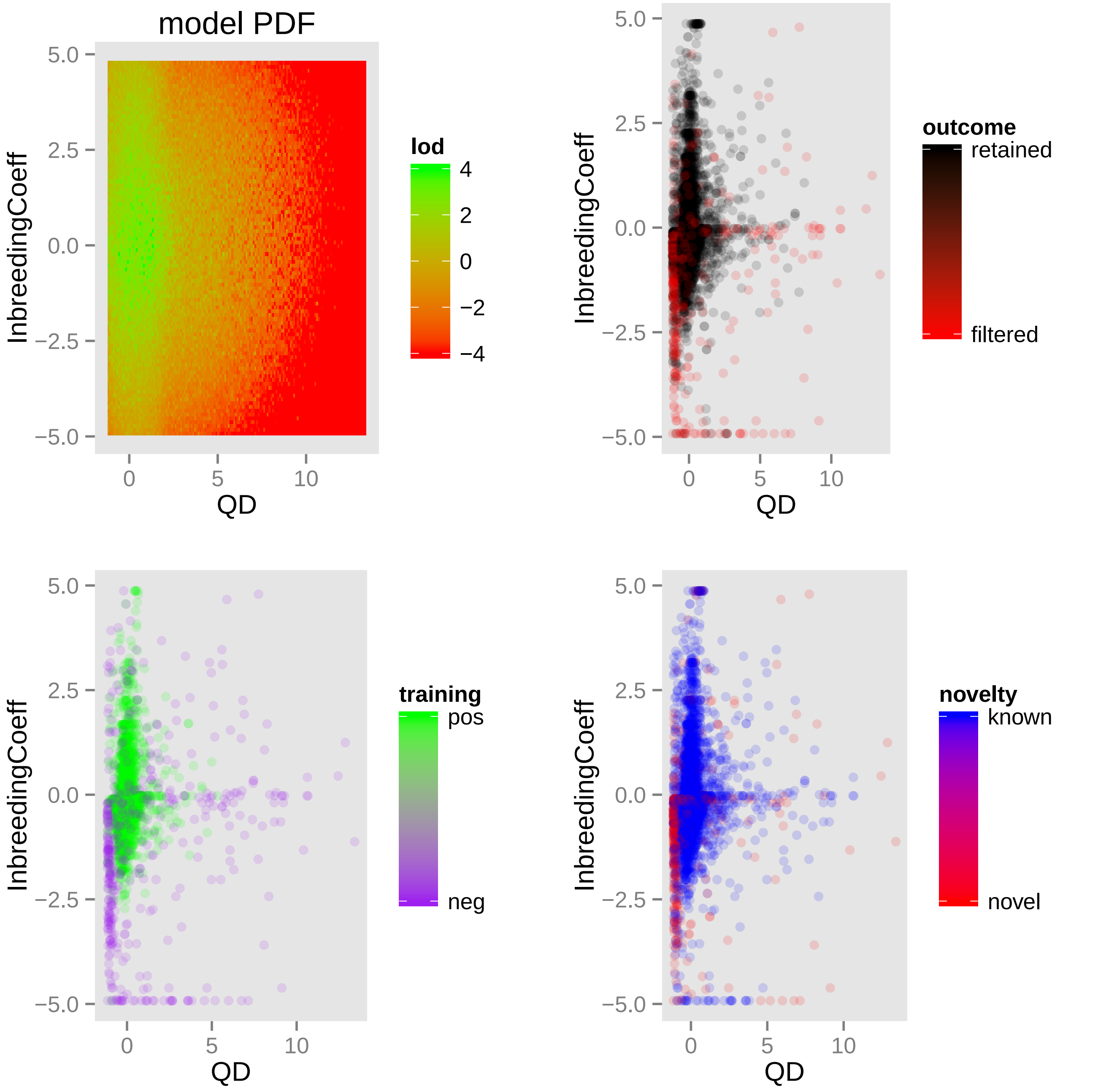
model PDF
lod
4
2
0
−2
−4
outcome
retained
filtered
training
pos
neg
novelty
known
novel
InbreedingCoeff
QD
5.0
2.5
0.0
−2.5
−5.0
0
5
10

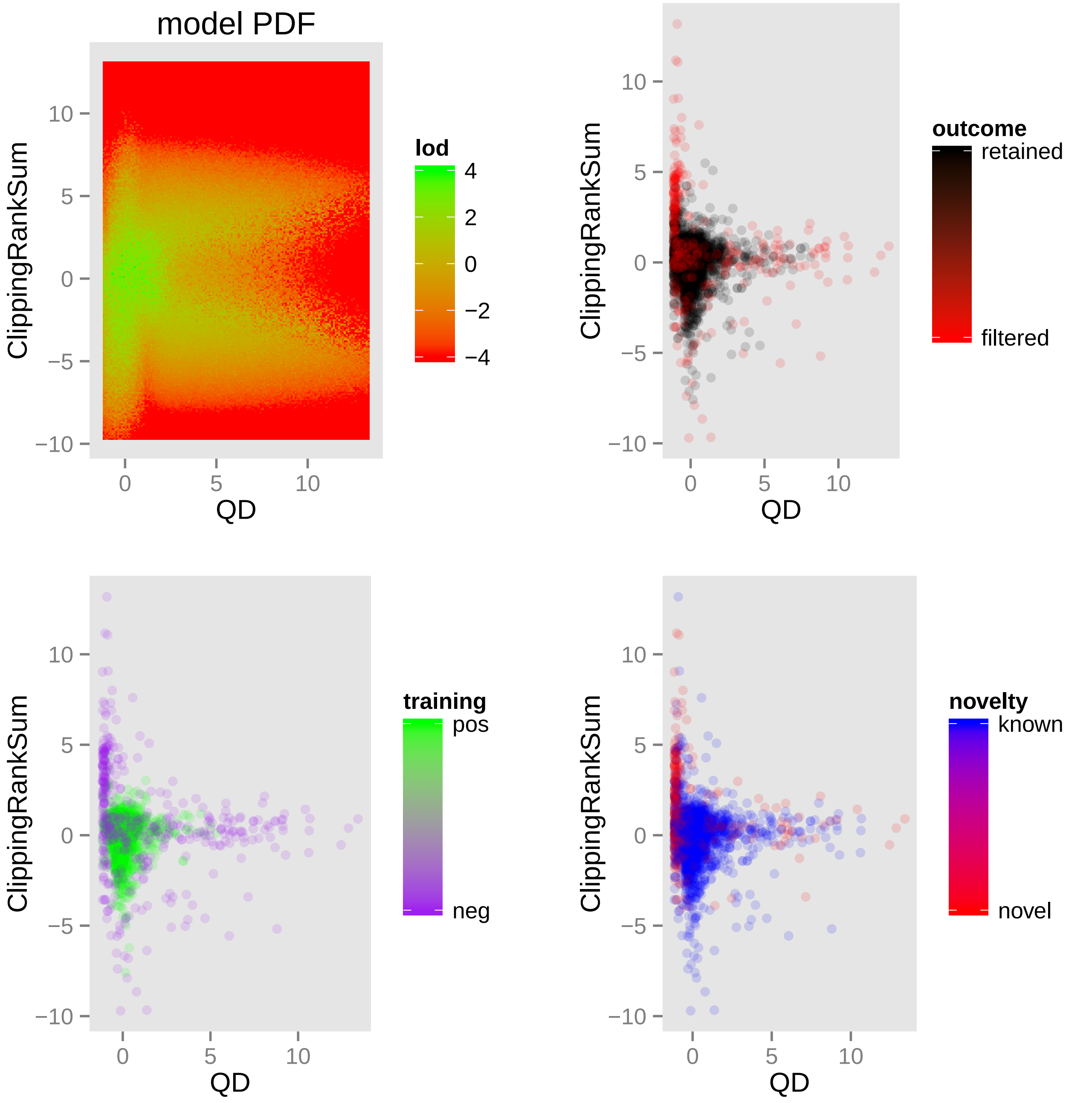

model PDF
ClippingRankSum
QD
lod
4
2
0
−2
−4
outcome
retained
filtered
training
pos
neg
novelty
known
novel
10
5
0
−5
−10

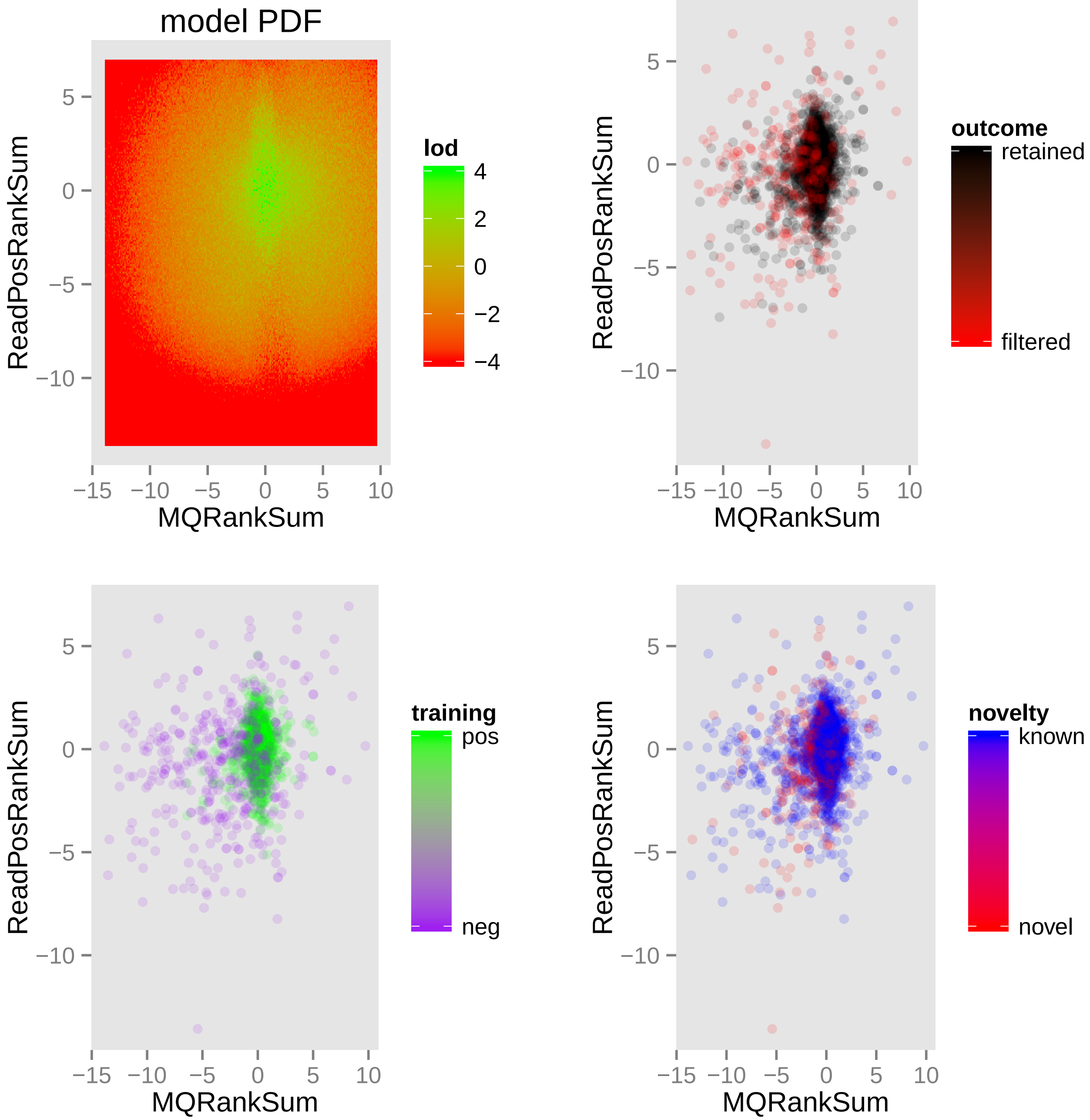
model PDF
lod
4
2
0
−2
−4
outcome
retained
filtered
training
pos
neg
novelty
known
novel
ReadPosRankSum
MQRankSum
5
0
−5
−10
−15
−10
−5
0
5
10

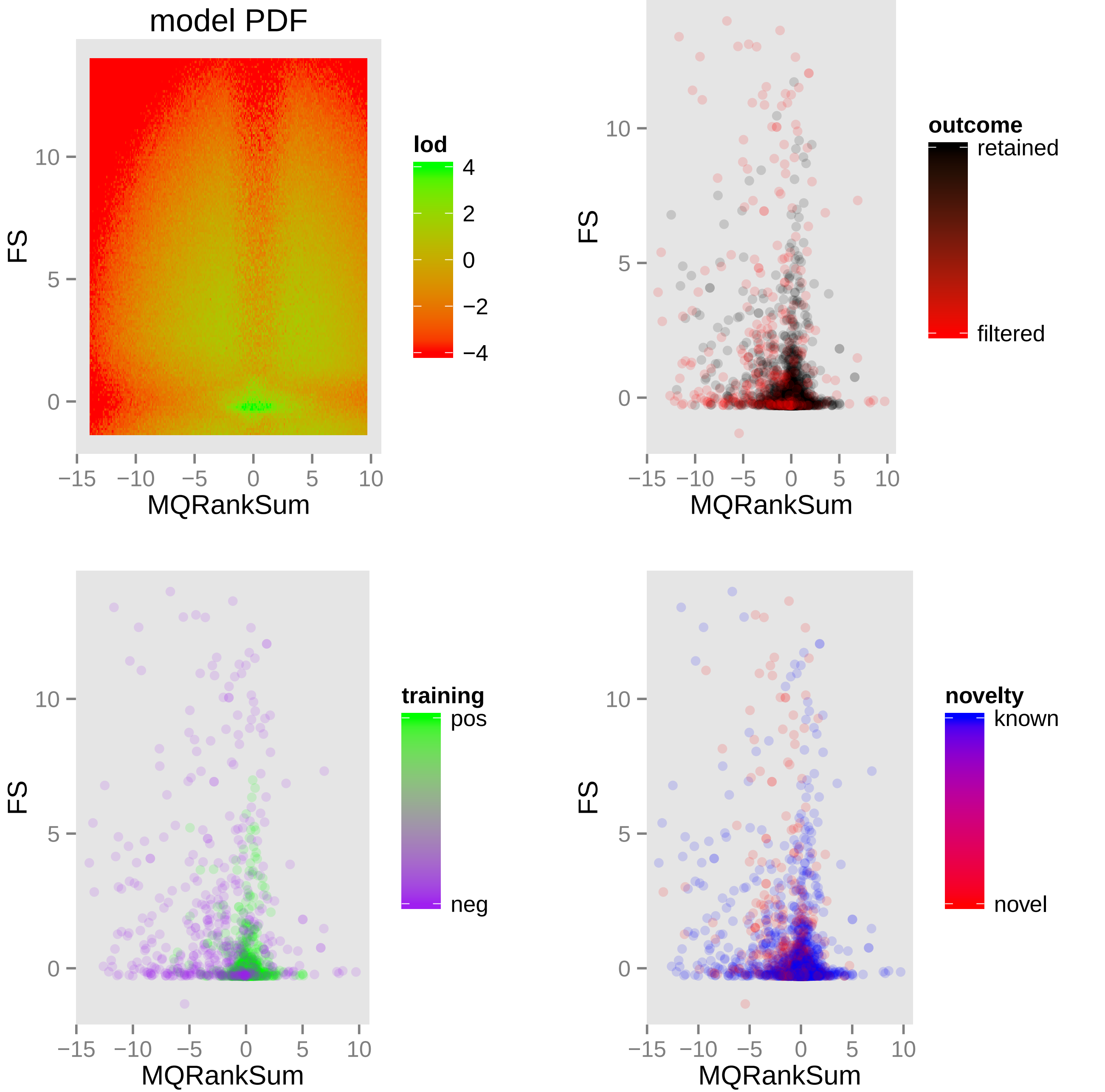
model PDF
FS
MQRankSum
lod
4
2
0
−2
−4
outcome
retained
filtered
training
pos
neg
novelty
known
novel
10
5
0
−15
−10
−5
0
5
10

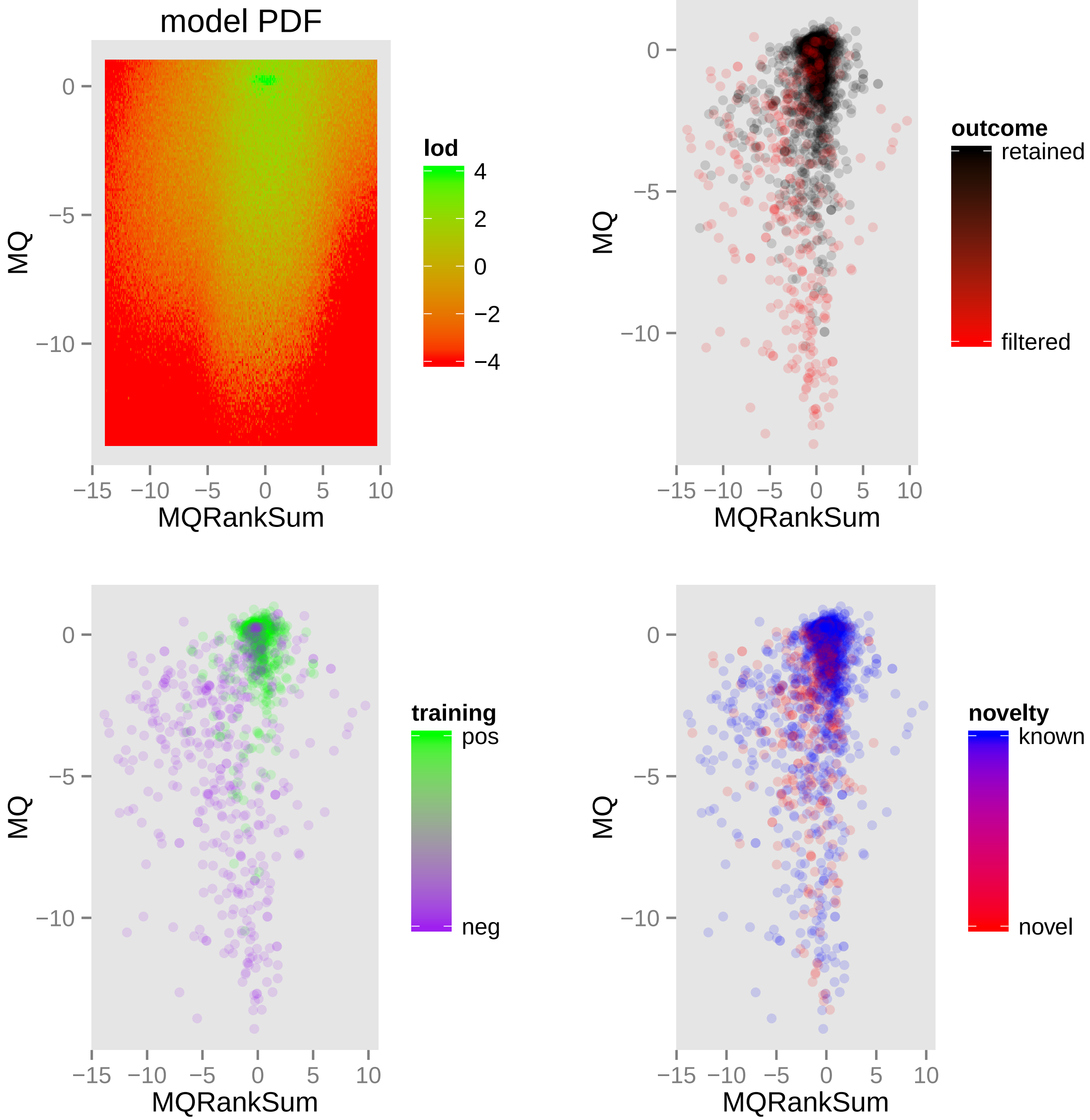

model PDF
MQ
MQRankSum
lod
4
2
0
−2
−4
0
−5
−10
−15
−10
−5
0
5
10
outcome
retained
filtered
training
pos
neg
novelty
known
novel

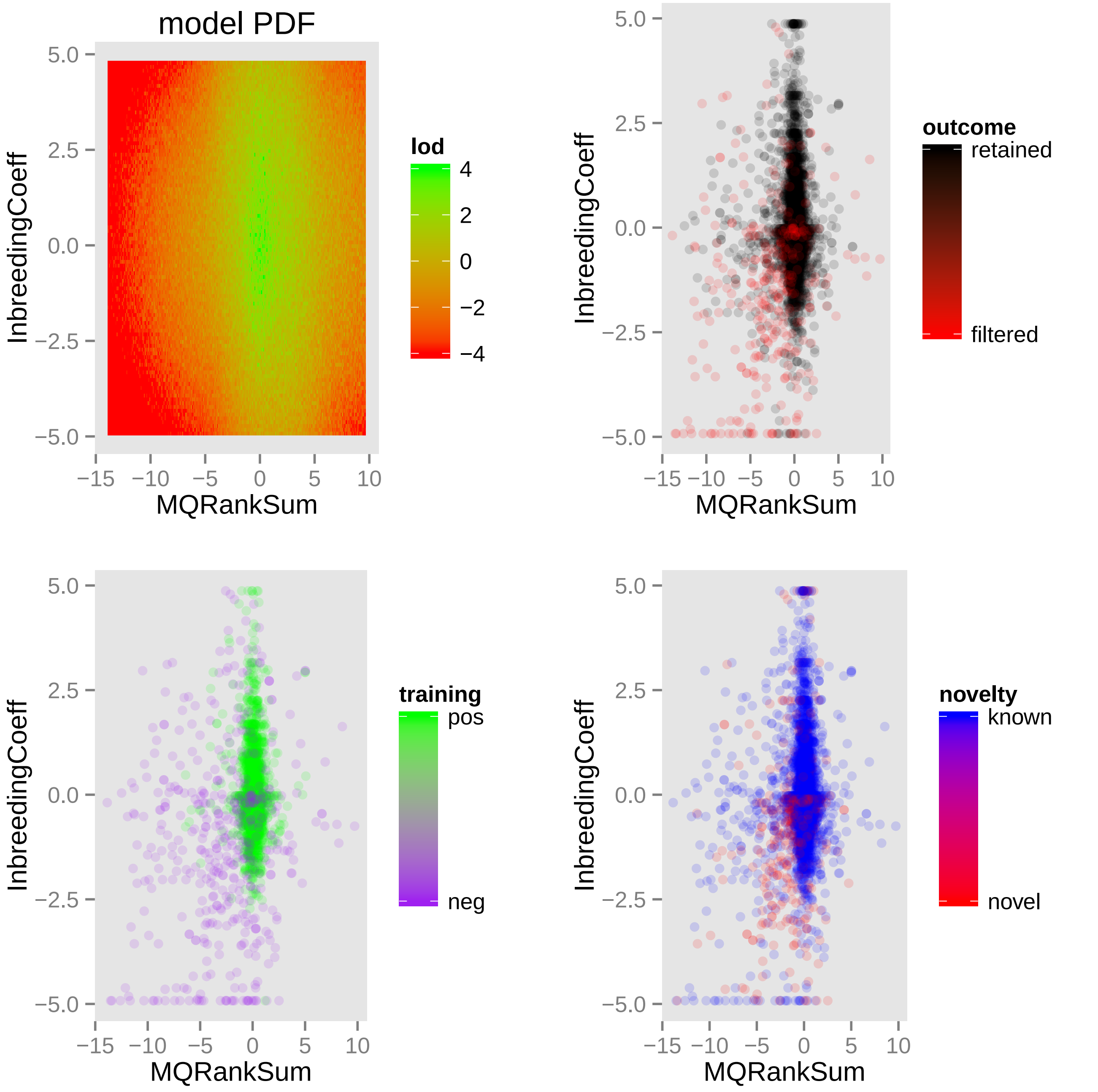
model PDF
lod
4
2
0
−2
−4
outcome
retained
filtered
training
pos
neg
novelty
known
novel
InbreedingCoeff
MQRankSum
5.0
2.5
0.0
−2.5
−5.0
−15
−10
−5
0
5
10

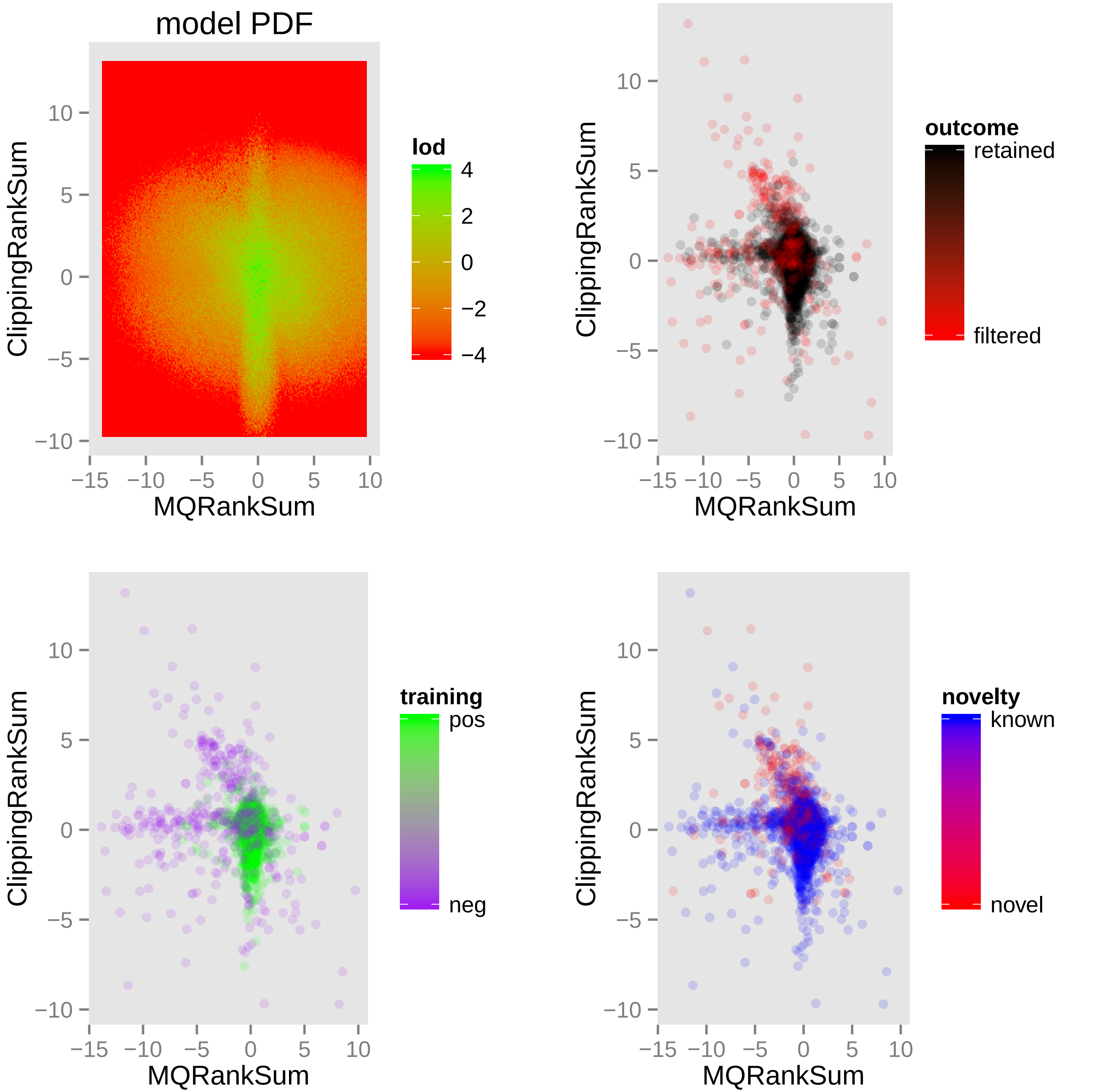
model PDF
lod
4
2
0
−2
−4
outcome
retained
filtered
training
pos
neg
novelty
known
novel
ClippingRankSum
MQRankSum
10
5
0
−5
−10
−15

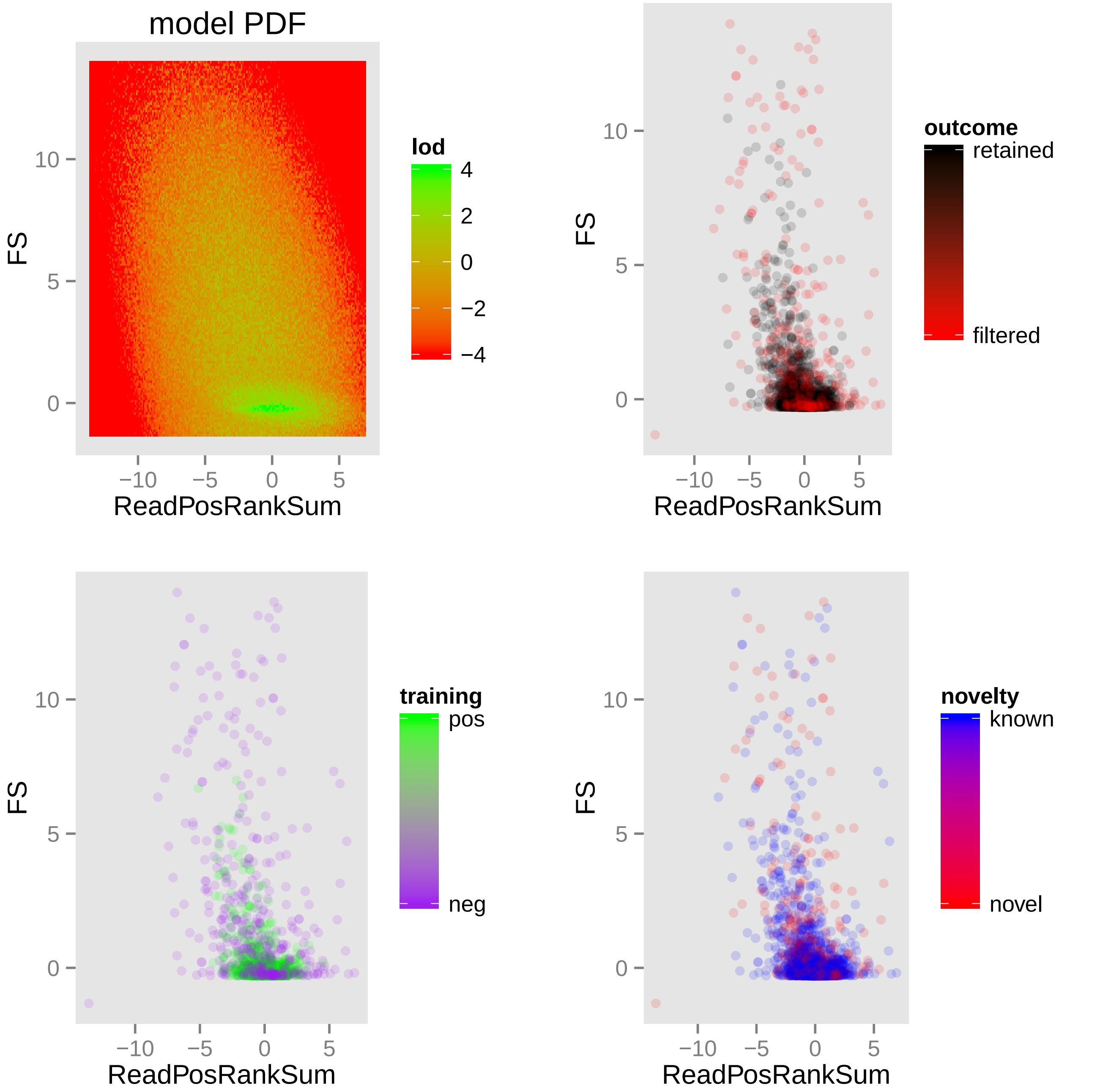
model PDF
lod
4
2
0
−2
−4
FS
ReadPosRankSum
−10
−5
0
5
10
outcome
retained
filtered
training
pos
neg
novelty
known
novel

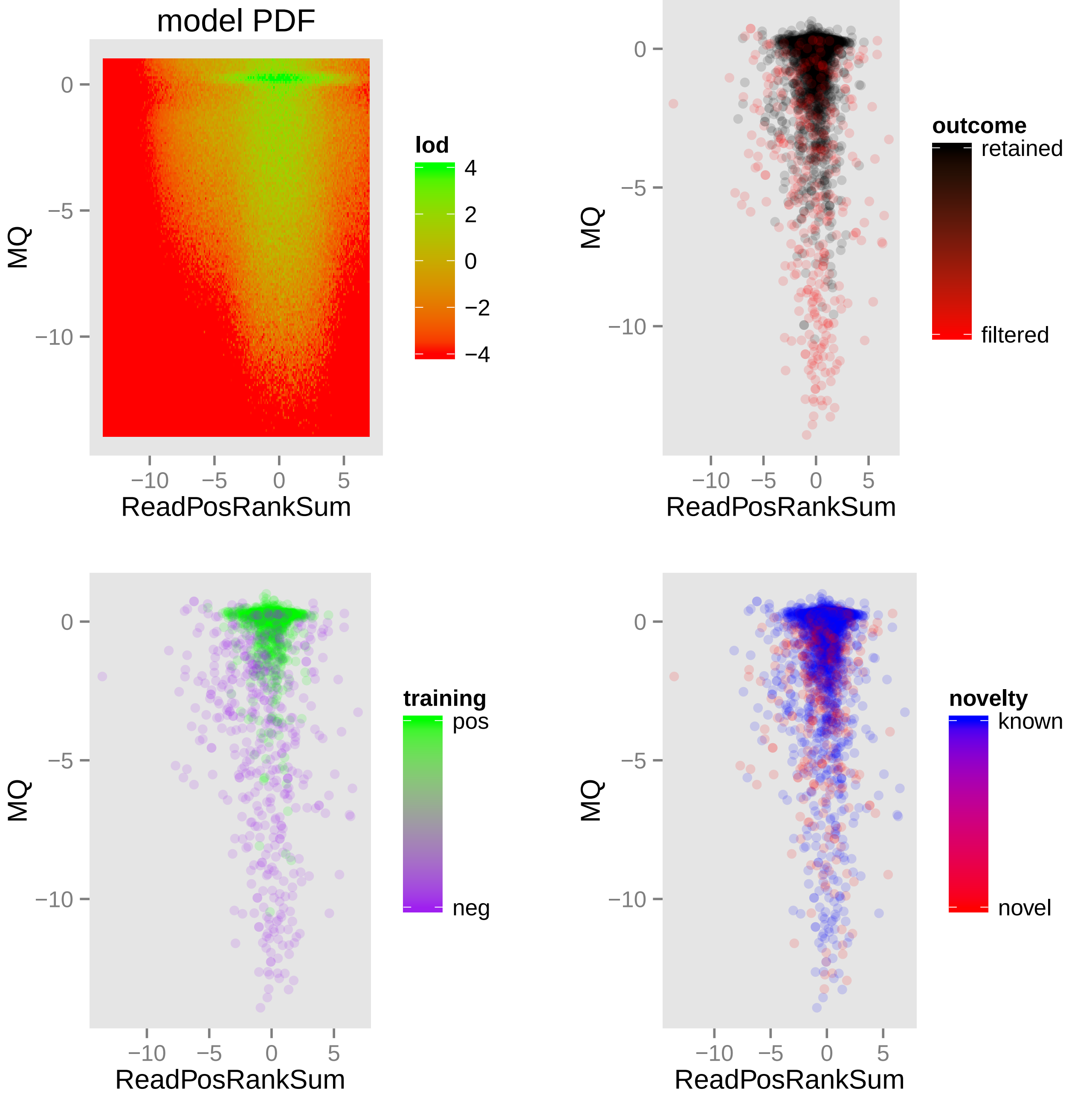
model PDF
lod
4
2
0
−2
−4
MQ
ReadPosRankSum
outcome
retained
filtered
training
pos
neg
novelty
known
novel

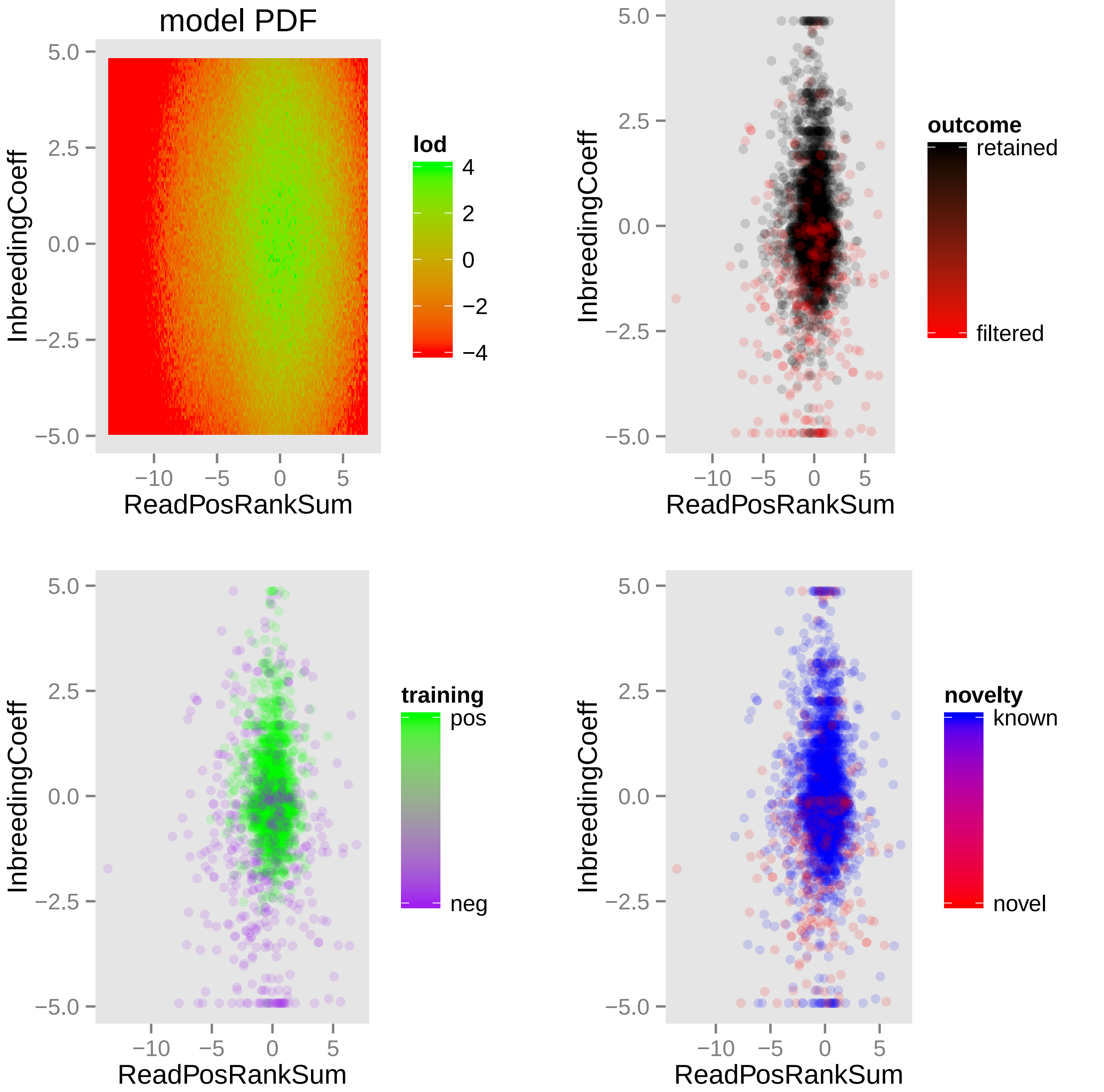
model PDF
lod
4
2
0
−2
−4
5.0
2.5
0.0
−2.5
−5.0
InbreedingCoeff
−10
−5
0
5
ReadPosRankSum
outcome
retained
filtered
training
pos
neg
novelty
known
novel

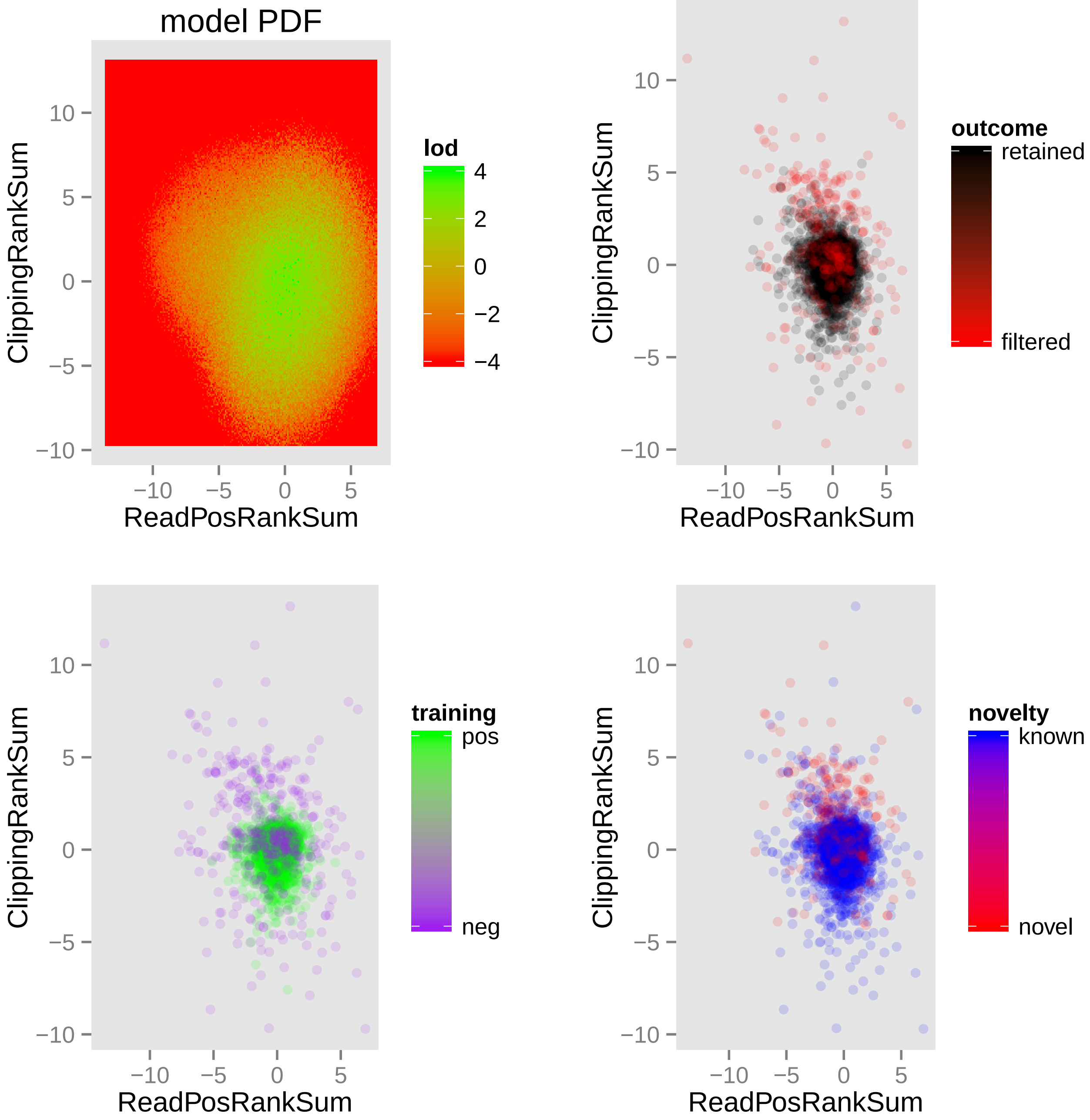
model PDF
lod
4
2
0
−2
−4
ClippingRankSum
ReadPosRankSum
10
5
0
−5
−10
outcome
retained
filtered
training
pos
neg
novelty
known
novel

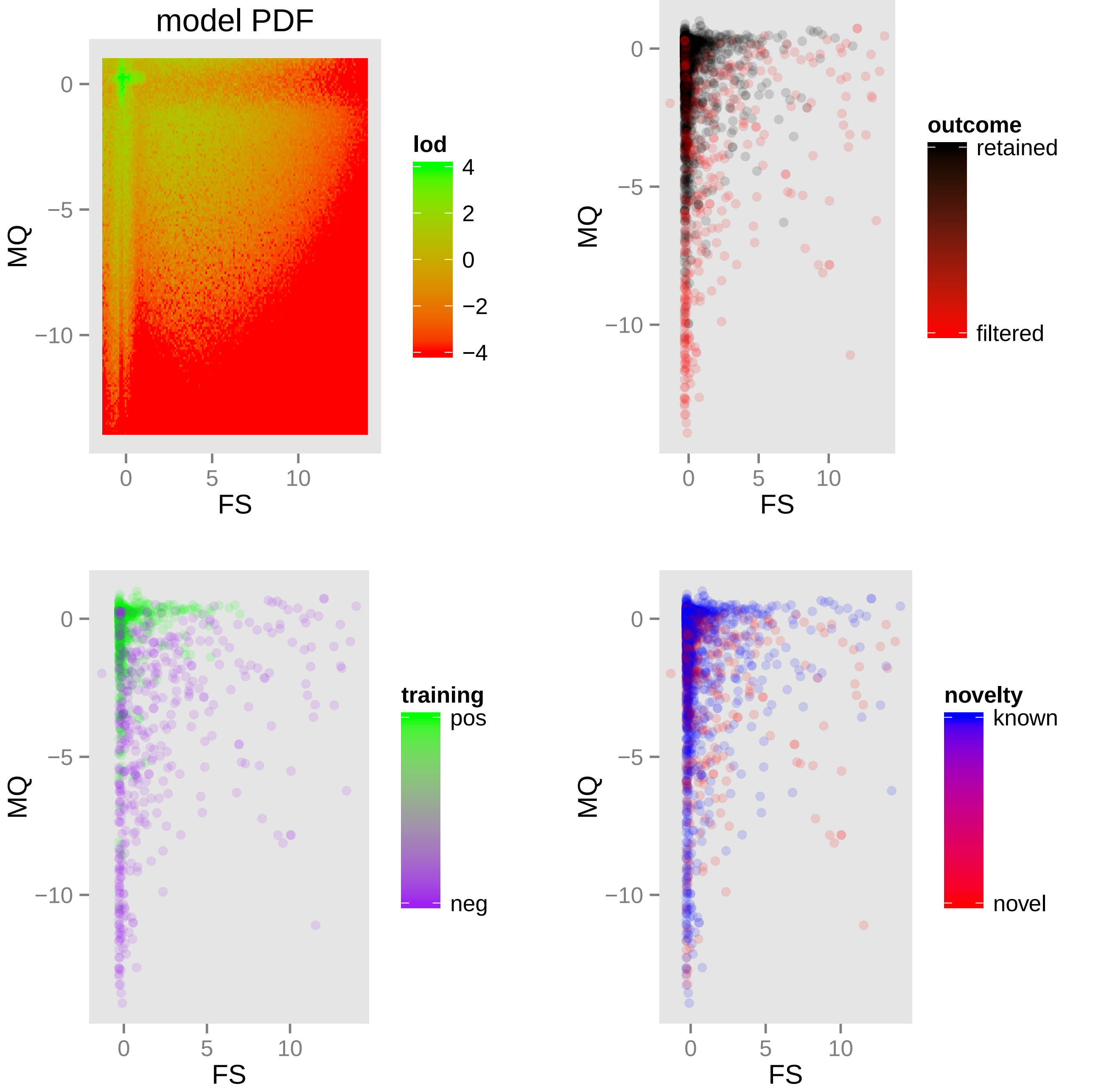
model PDF
MQ
FS
lod
4
2
0
−2
−4
0
−5
−10
0
5
10
outcome
retained
filtered
training
pos
neg
novelty
known
novel

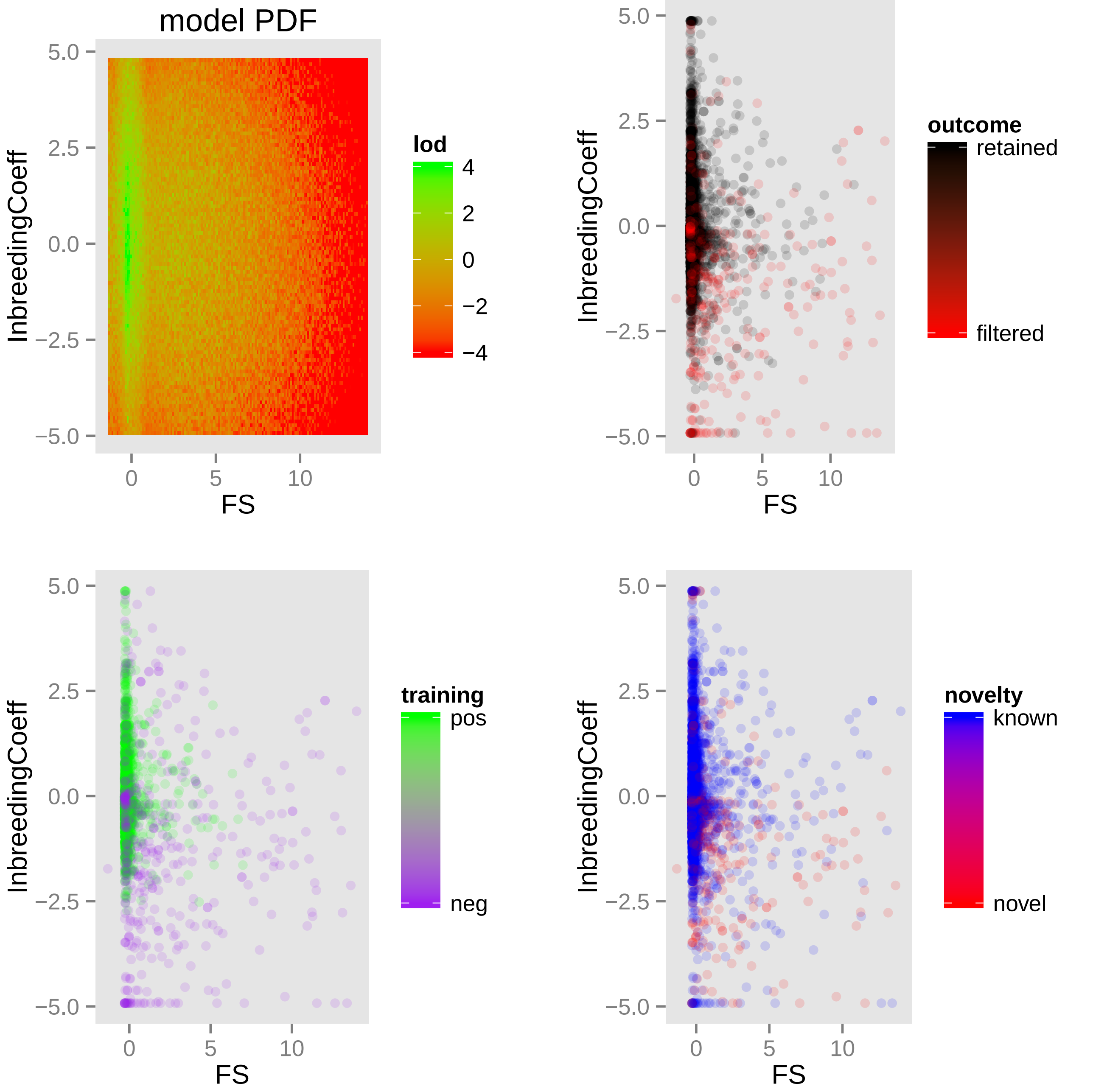
model PDF
lod
4
2
0
−2
−4
outcome
retained
filtered
training
pos
neg
novelty
known
novel
InbreedingCoeff
FS
5.0
2.5
0.0
−2.5
−5.0
0
5
10

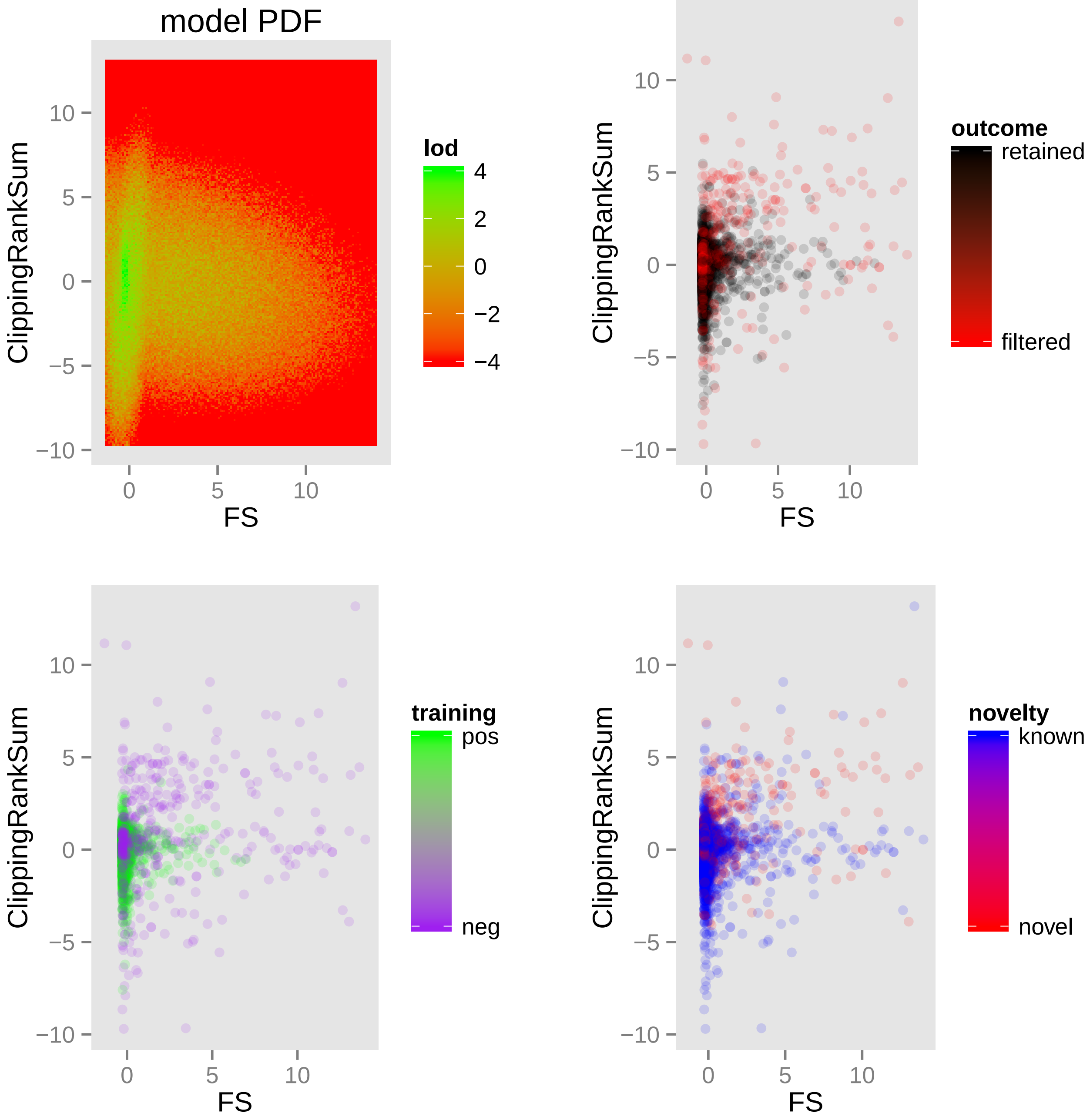
model PDF
lod
4
2
0
−2
−4
ClippingRankSum
FS
outcome
retained
filtered
training
pos
neg
novelty
known
novel

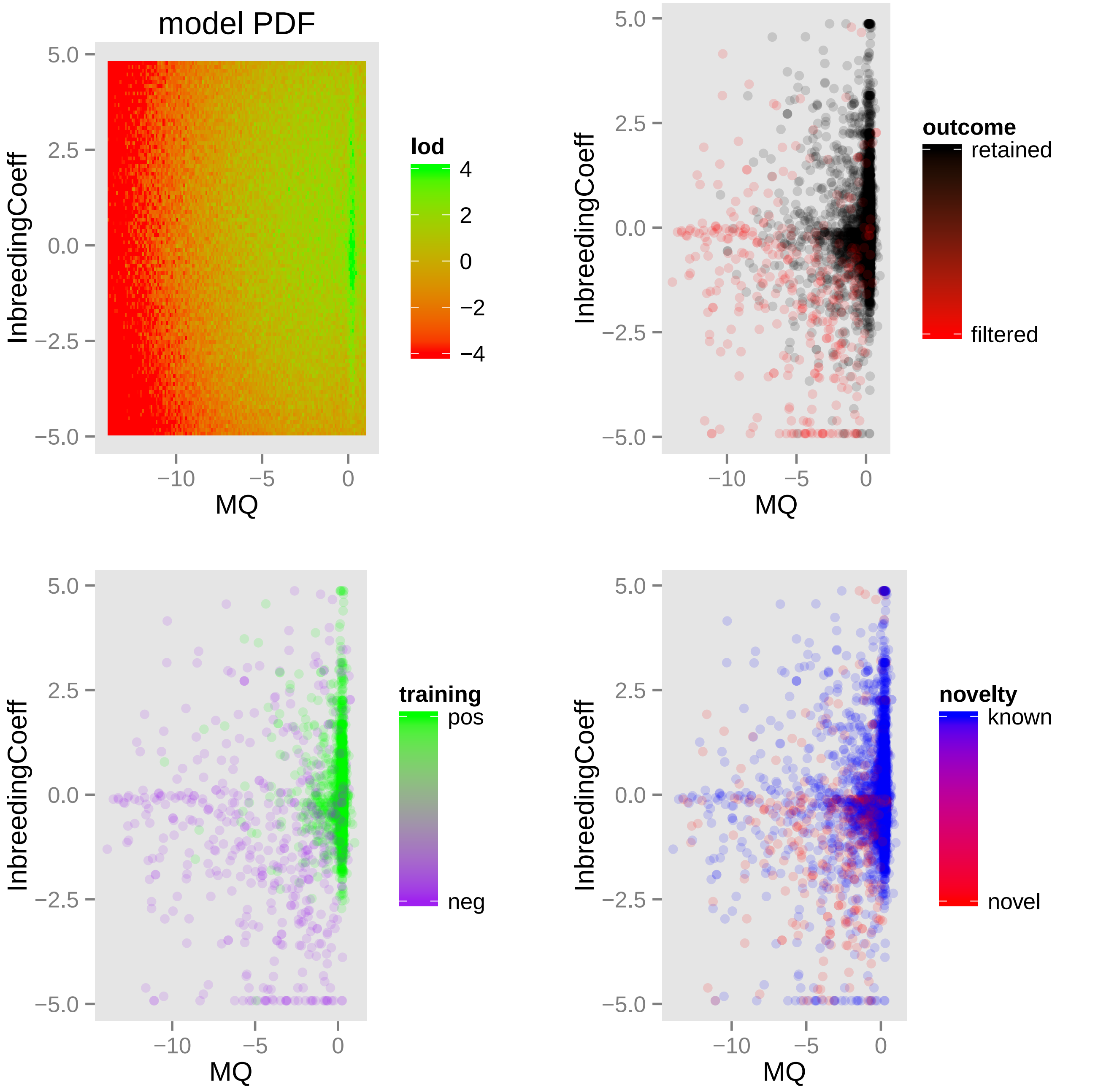
model PDF
InbreedingCoeff
MQ
5.0
2.5
0.0
−2.5
−5.0
−10
−5
0
lod
4
2
0
−2
−4
outcome
retained
filtered
training
pos
neg
novelty
known
novel

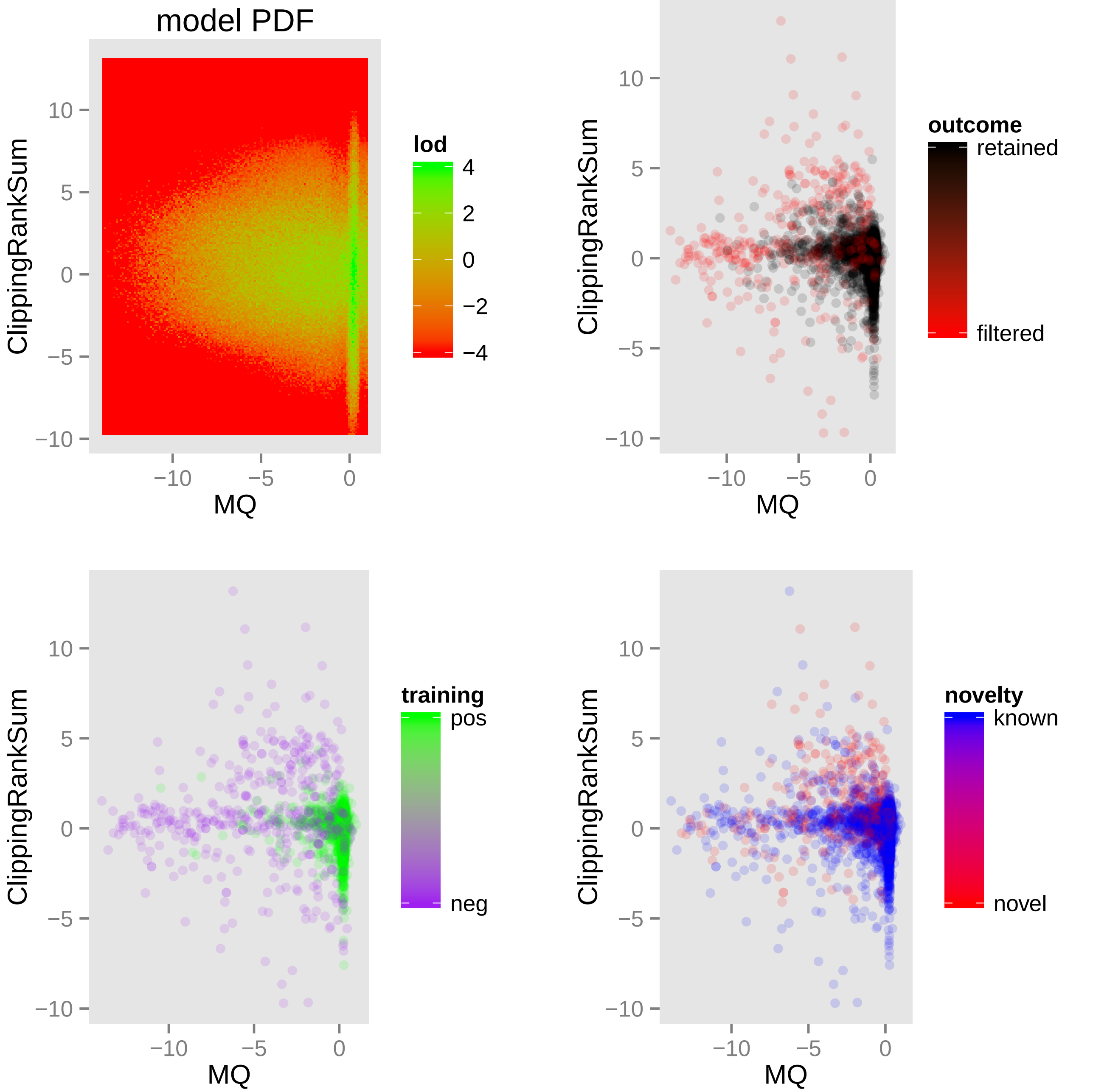
model PDF
lod
4
2
0
−2
−4
ClippingRankSum
MQ
10
5
0
−5
−10
outcome
retained
filtered
training
pos
neg
novelty
known
novel

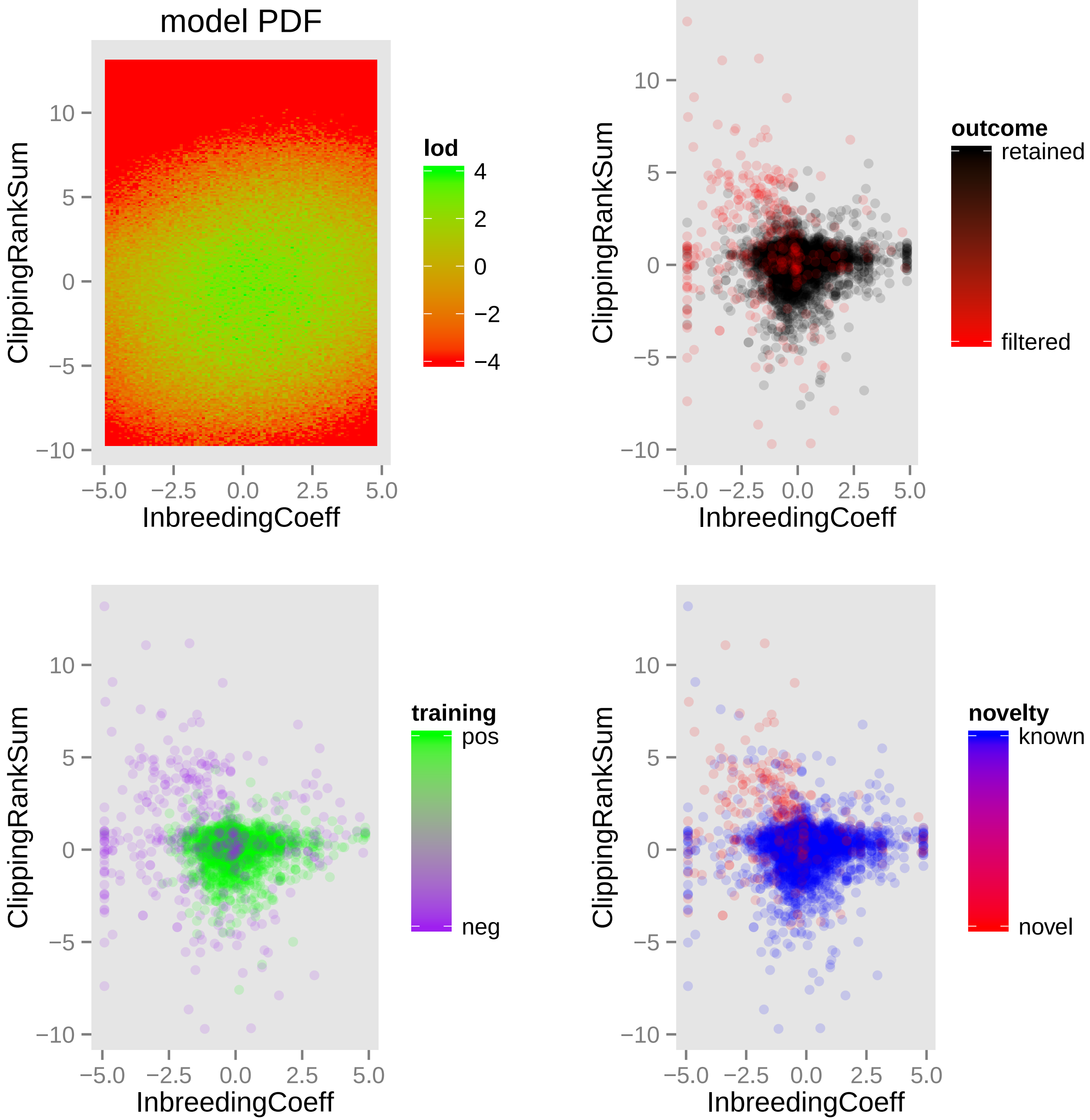
model PDF
lod
4
2
0
−2
−4
ClippingRankSum
InbreedingCoeff
outcome
retained
filtered
training
pos
neg
novelty
known
novel
10
5
0
−5
−10
−5.0
−2.5
0.0
2.5
5.0

HaplotypeCaller 2.4.9 VQSR

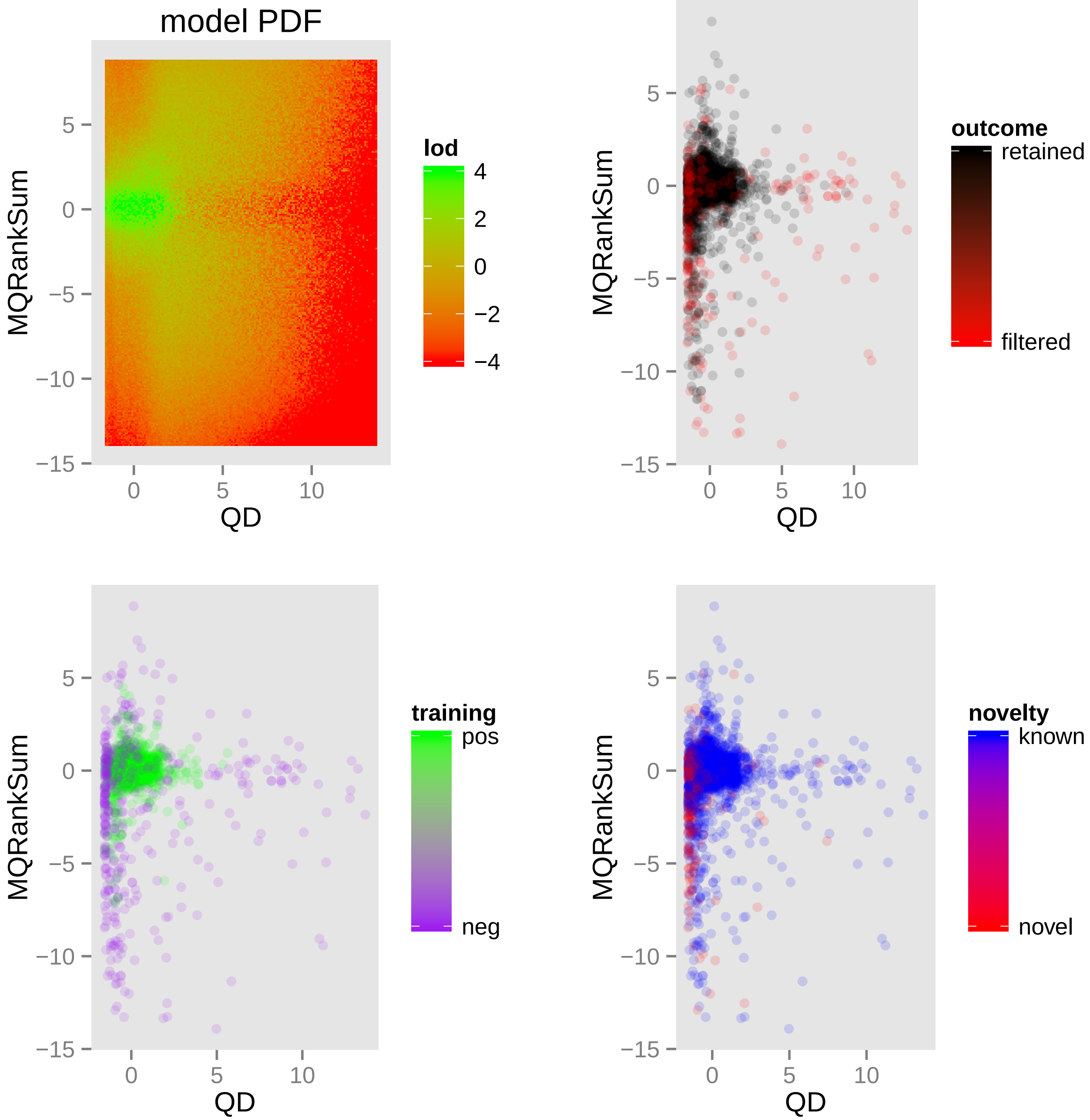

model PDF
lod
4
2
0
−2
−4
outcome
retained
filtered
training
pos
neg
novelty
known
novel
MQRankSum
QD
5
0
−5
−10
−15
10

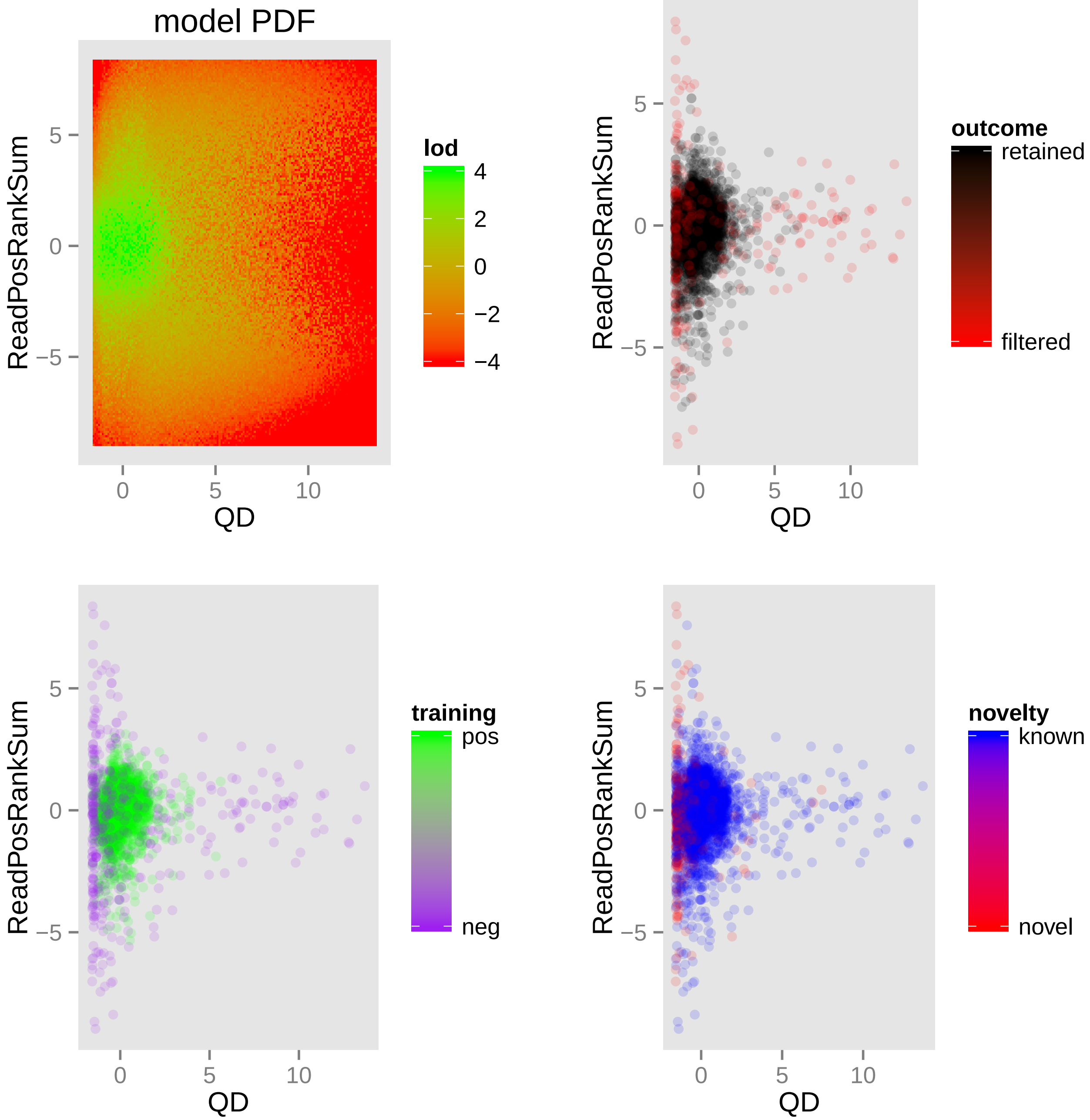
model PDF
ReadPosRankSum
QD
lod
4
2
0
−2
−4
outcome
retained
filtered
training
pos
neg
novelty
known
novel
5
0
−5
10

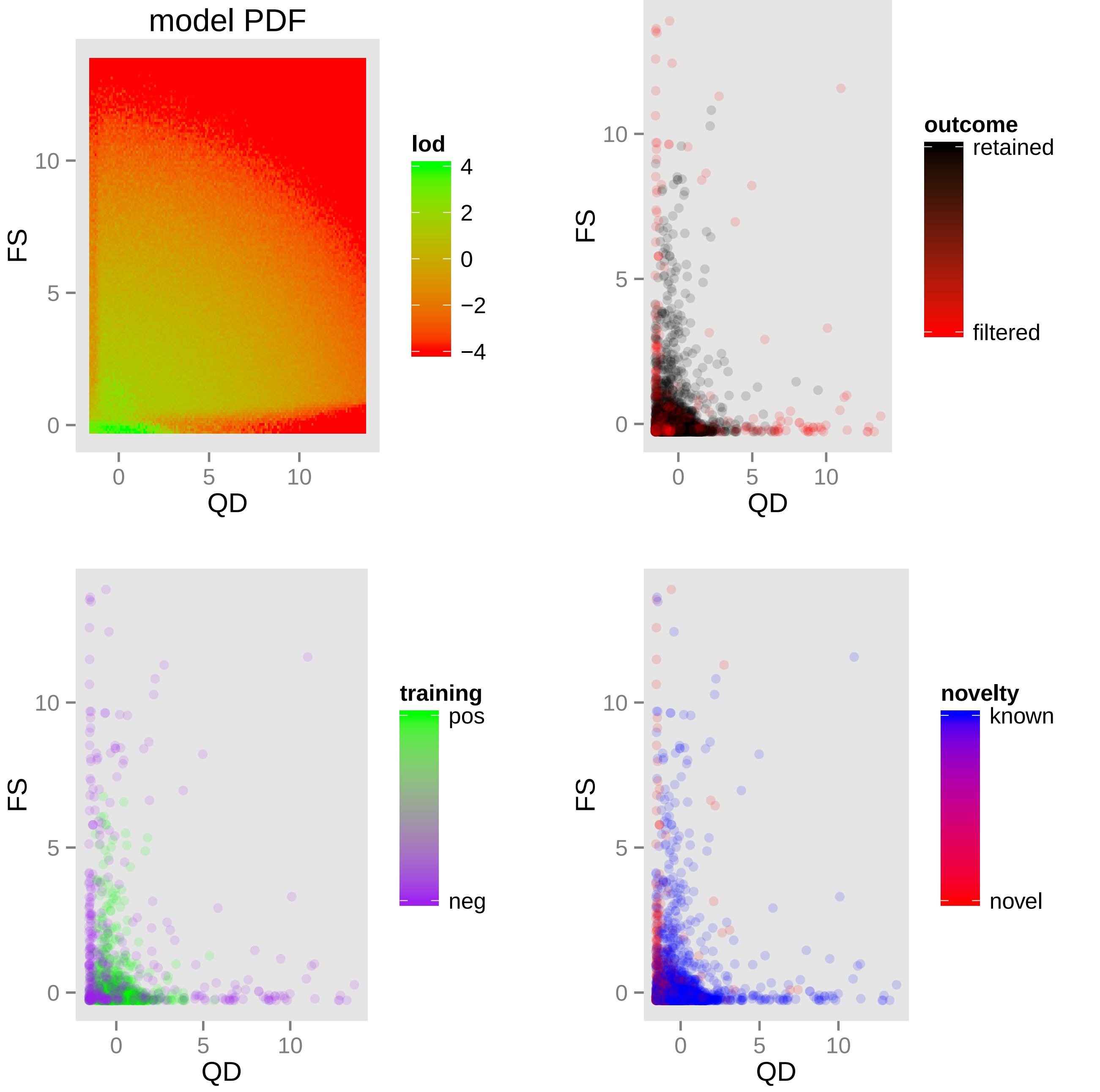

model PDF
lod
4
2
0
−2
−4
FS
QD
0
5
10
outcome
retained
filtered
training
pos
neg
novelty
known
novel

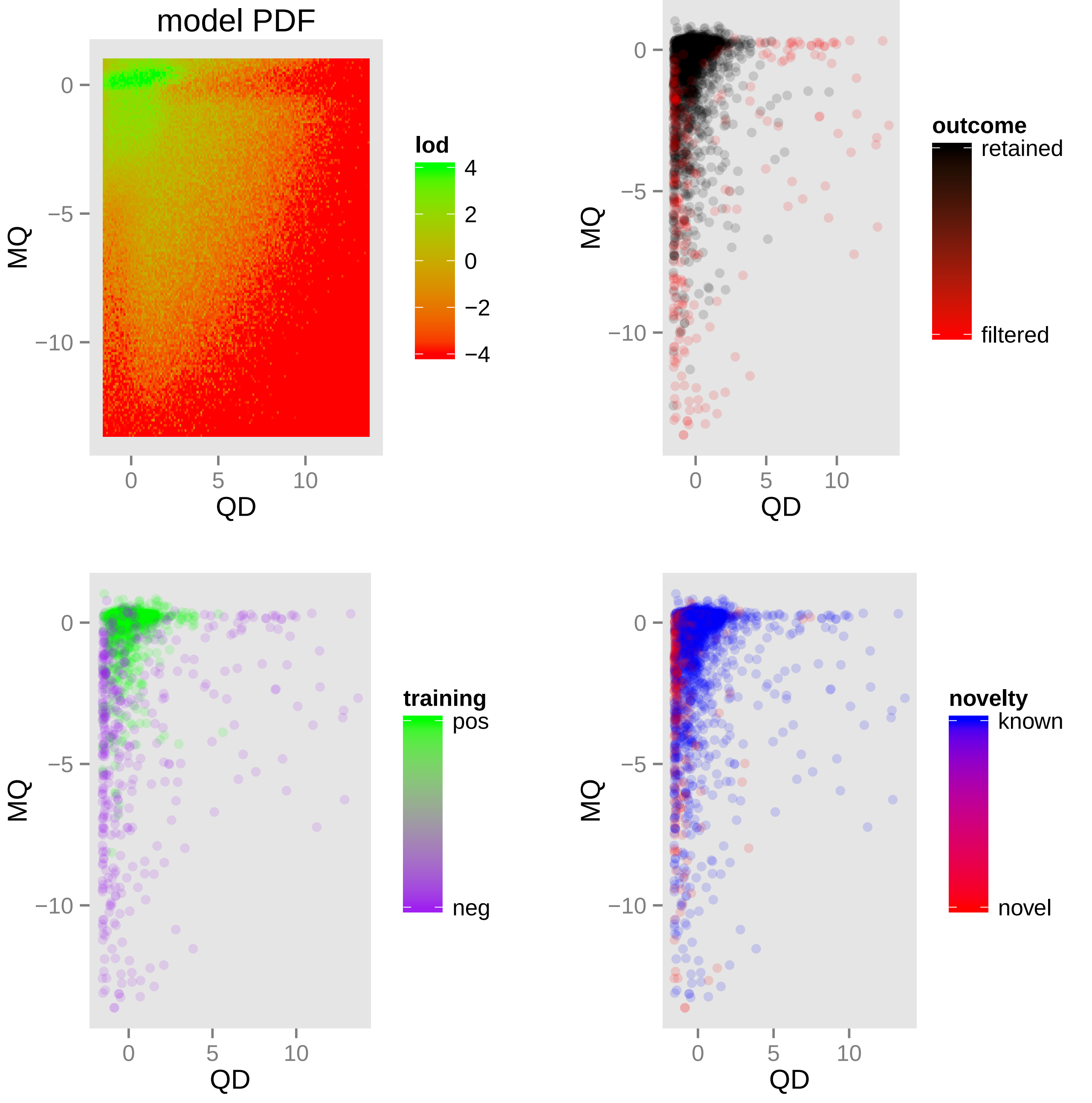
model PDF
lod
4
2
0
−2
−4
0
−5
−10
MQ
0
5
10
QD
outcome
retained
filtered
MQ
QD
training
pos
neg
MQ
QD
novelty
known
novel
MQ
QD

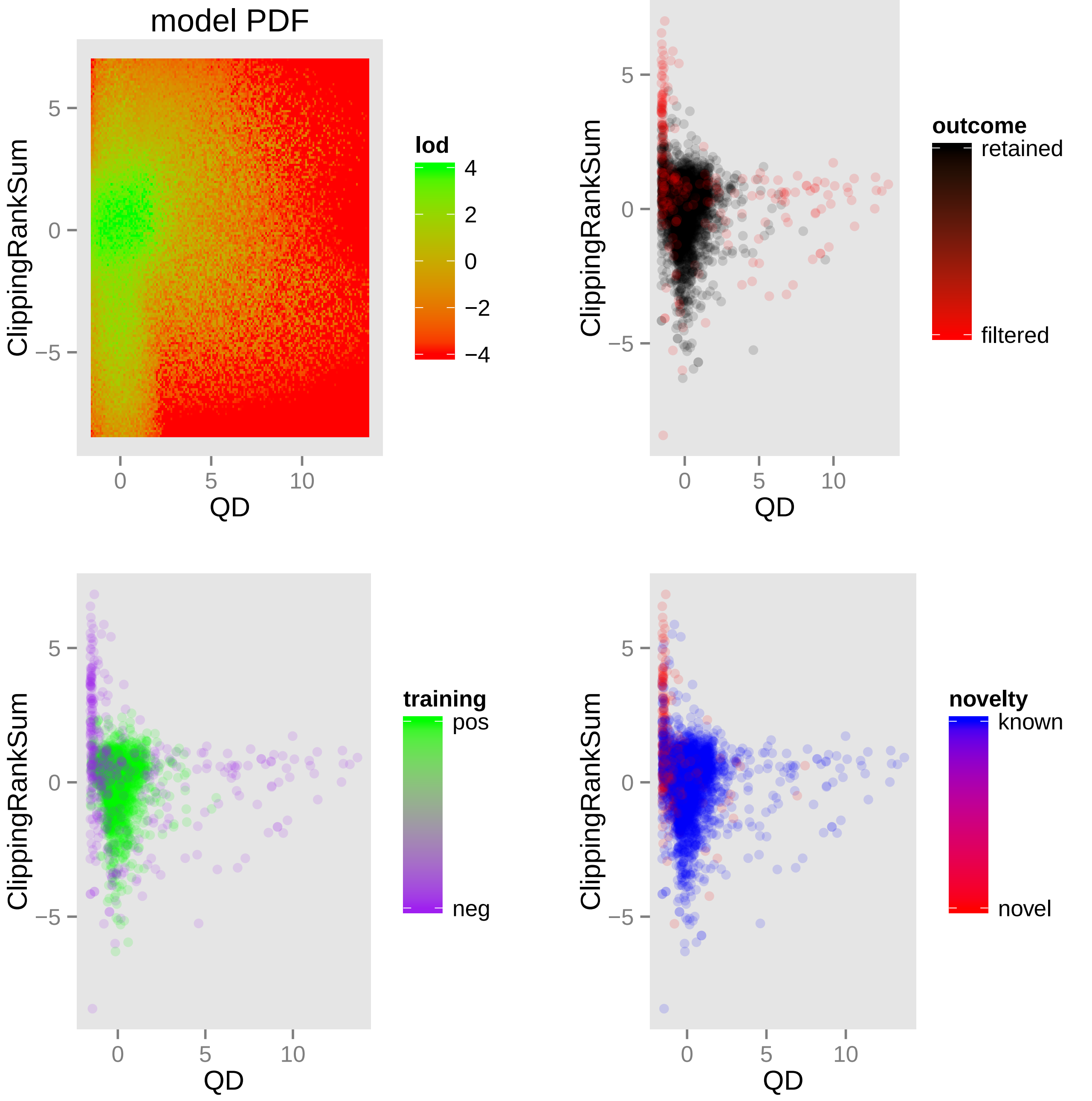
model PDF
ClippingRankSum
QD
lod
4
2
0
−2
−4
outcome
retained
filtered
training
pos
neg
novelty
known
novel

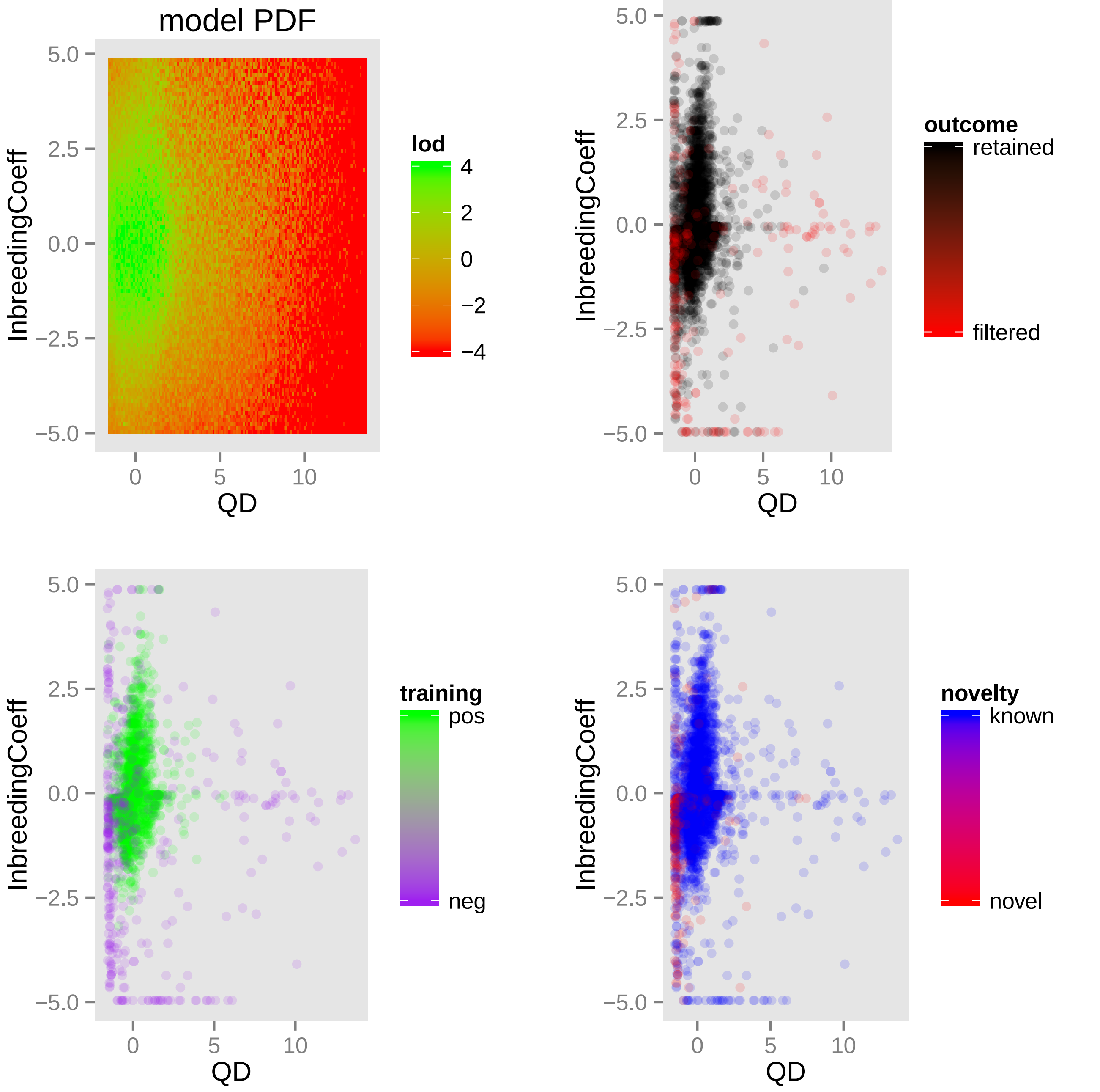

model PDF
InbreedingCoeff
QD
lod
4
2
0
−2
−4
5.0
2.5
0.0
−2.5
−5.0
0
5
10
outcome
retained
filtered
training
pos
neg
novelty
known
novel

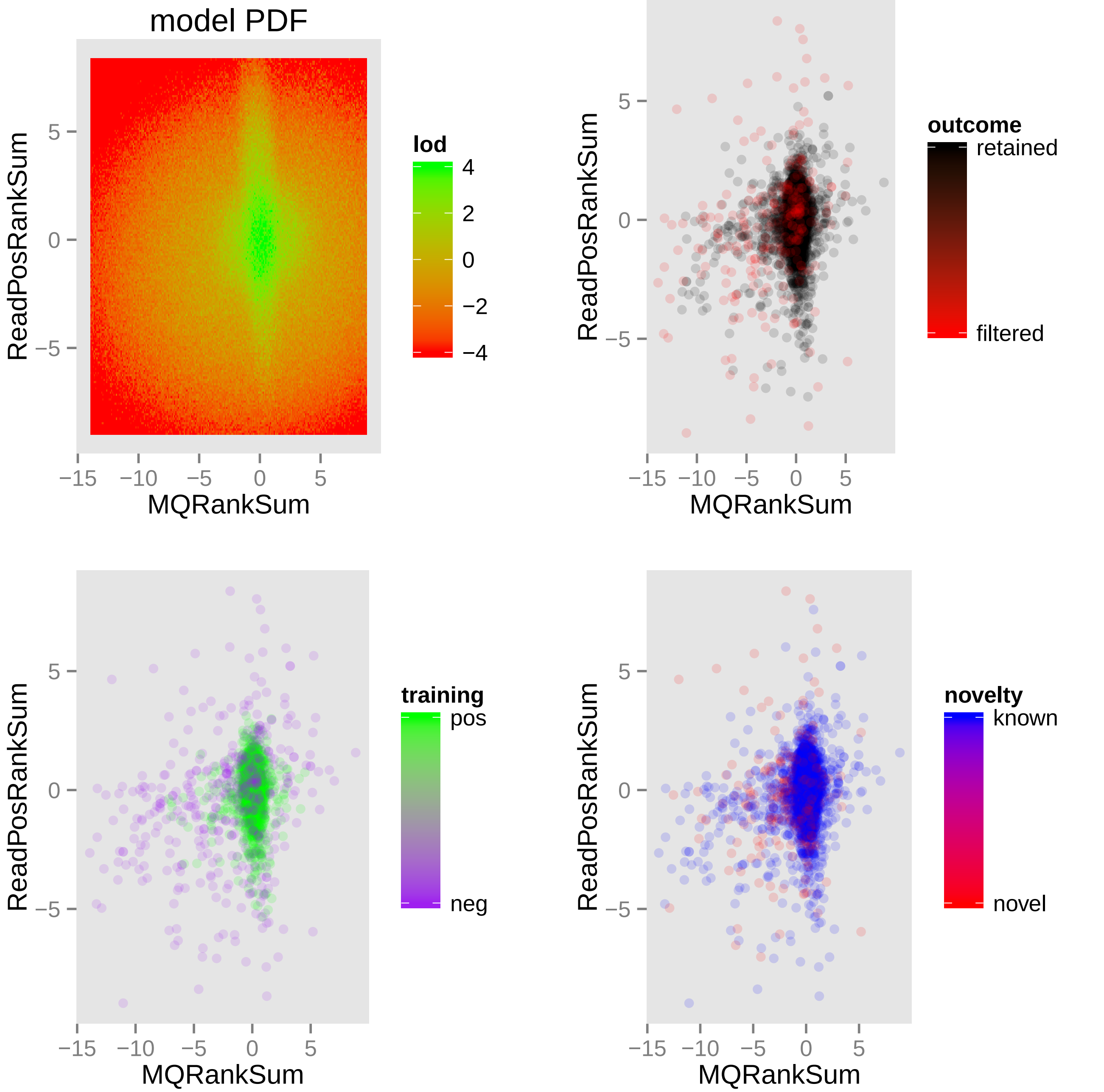

model PDF
ReadPosRankSum
MQRankSum
lod
4
2
0
−2
−4
outcome
retained
filtered
training
pos
neg
novelty
known
novel
−15
−10
−5
0
5

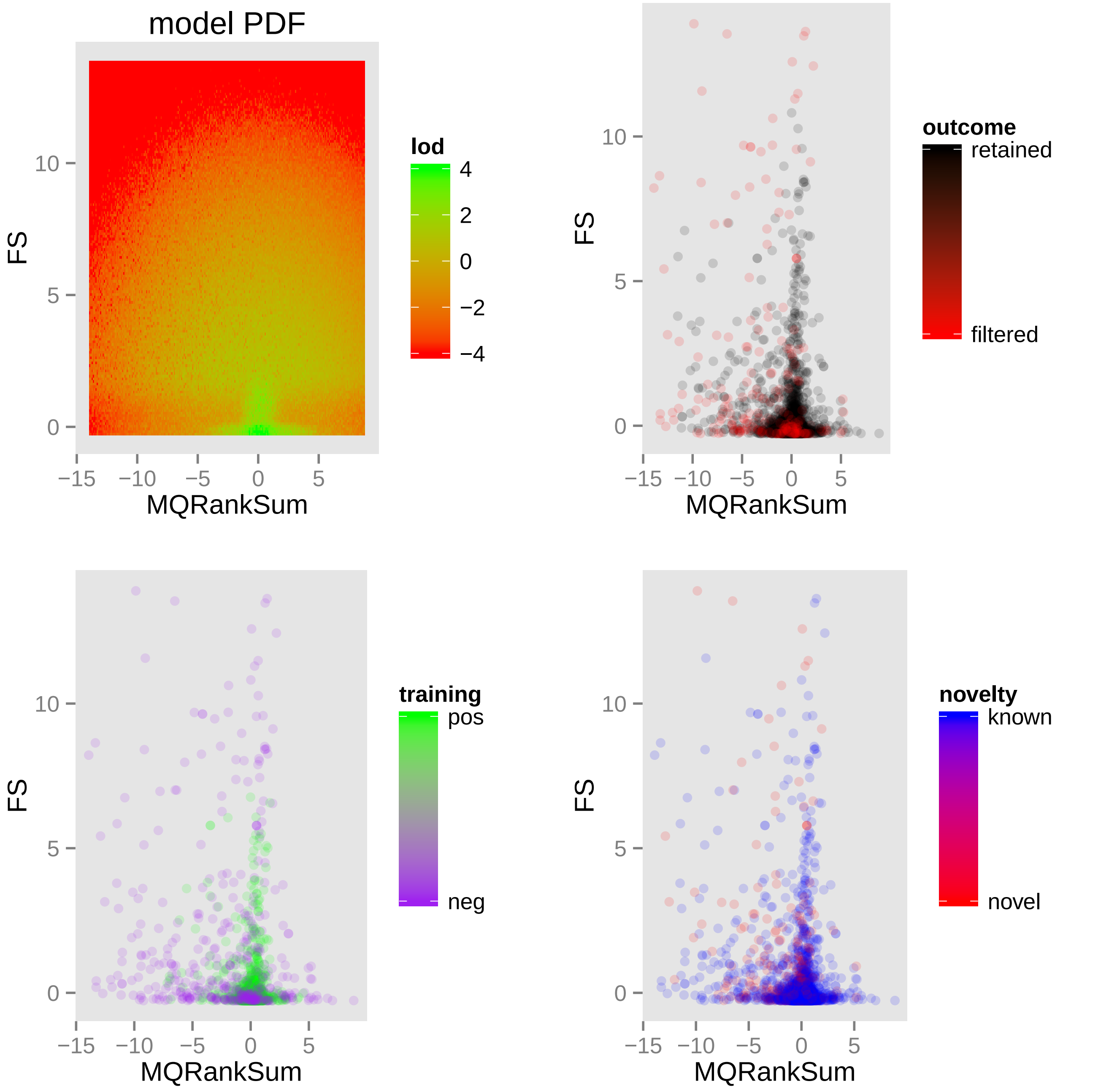
model PDF
lod
4
2
0
−2
−4
FS
MQRankSum
outcome
retained
filtered
training
pos
neg
novelty
known
novel
−15
−10
−5
0
5
10

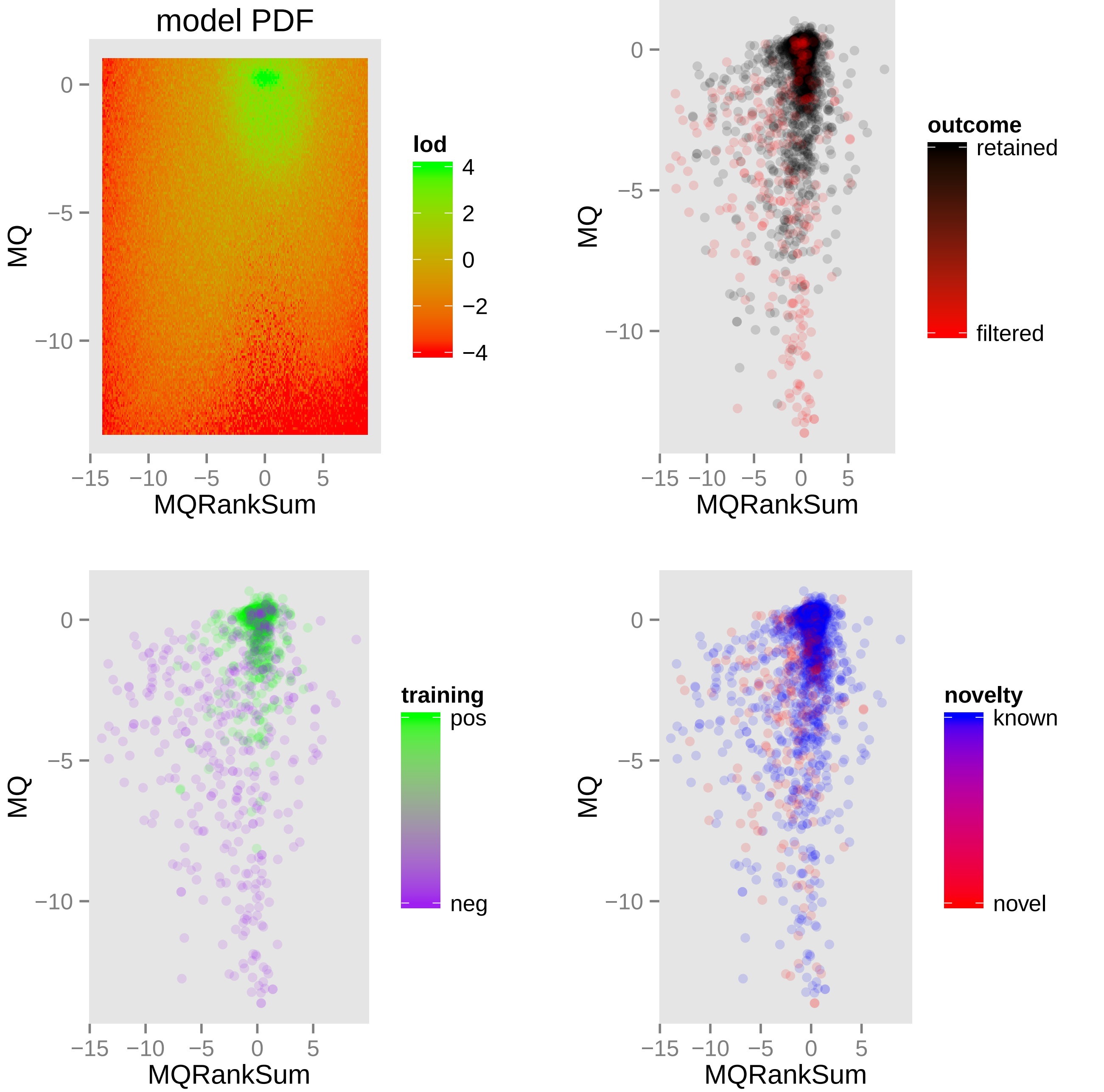

model PDF
MQ
MQRankSum
lod
4
2
0
−2
−4
outcome
retained
filtered
training
pos
neg
novelty
known
novel

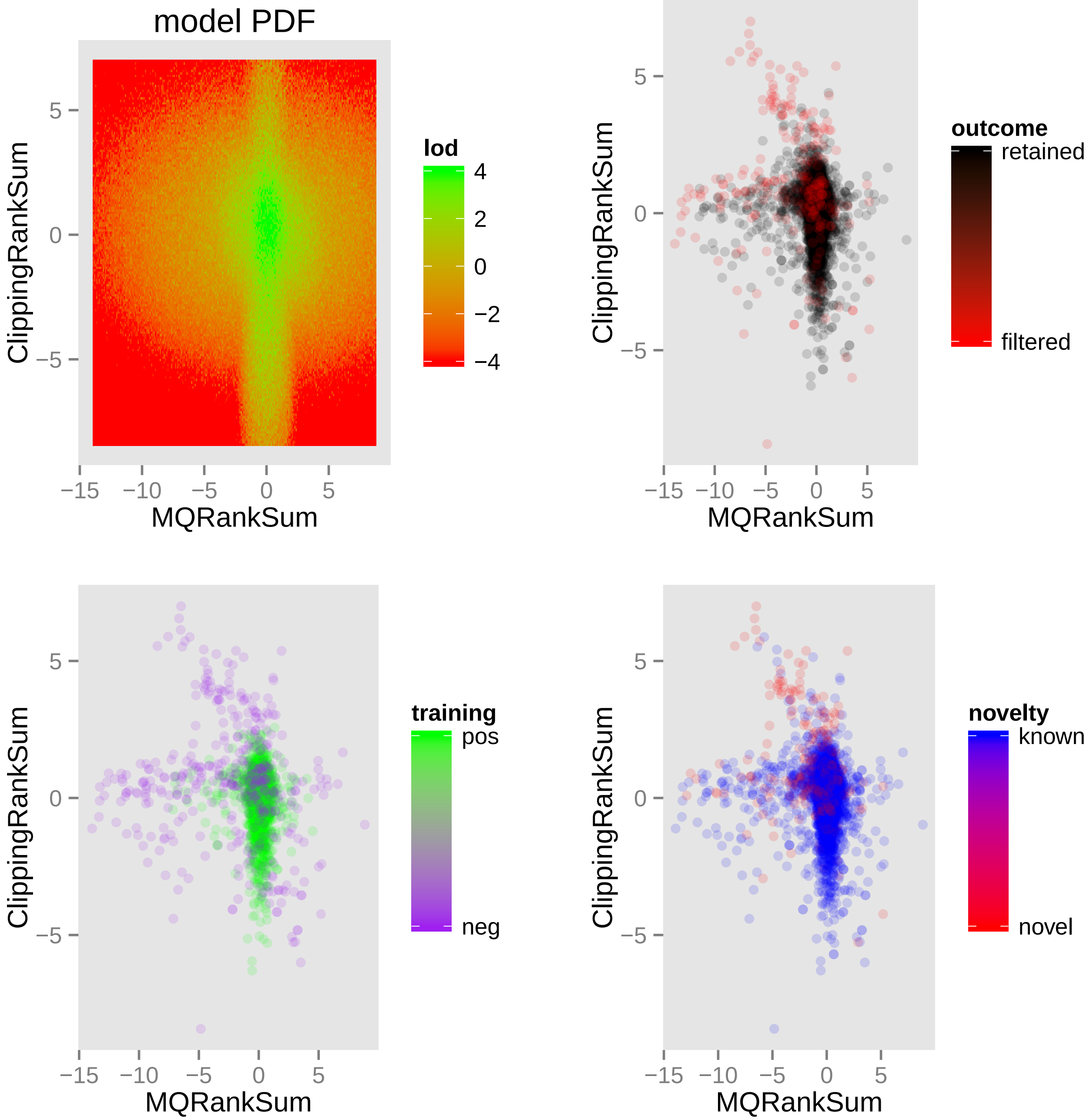
model PDF
lod
4
2
0
−2
−4
ClippingRankSum
MQRankSum
−15
−10
−5
0
5
outcome
retained
filtered
training
pos
neg
novelty
known
novel

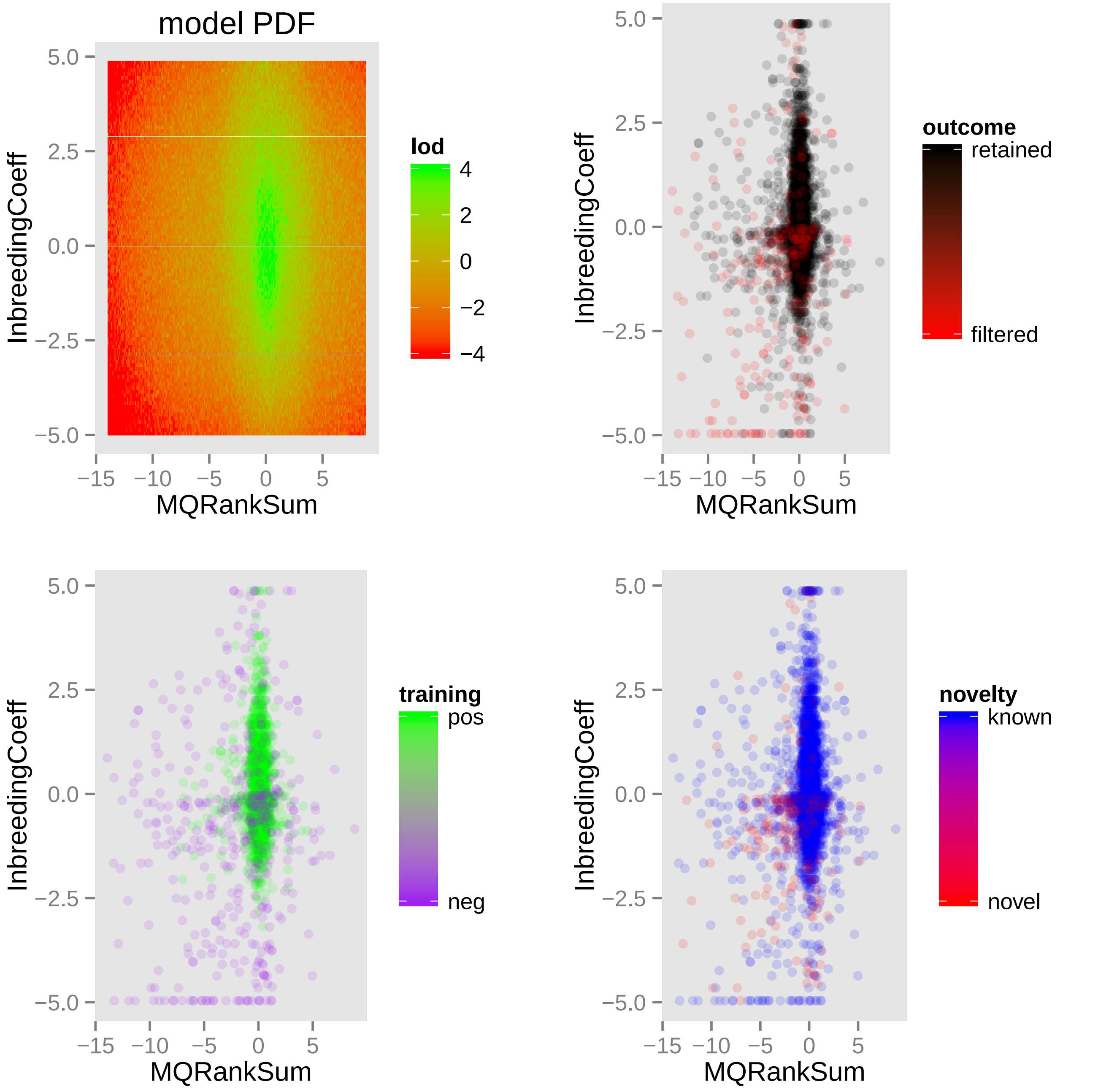

model PDF
InbreedingCoeff
MQRankSum
lod
4
2
0
−2
−4
outcome
retained
filtered
training
pos
neg
novelty
known
novel
5.0
2.5
0.0
−2.5
−5.0
−15
−10
−5
0
5

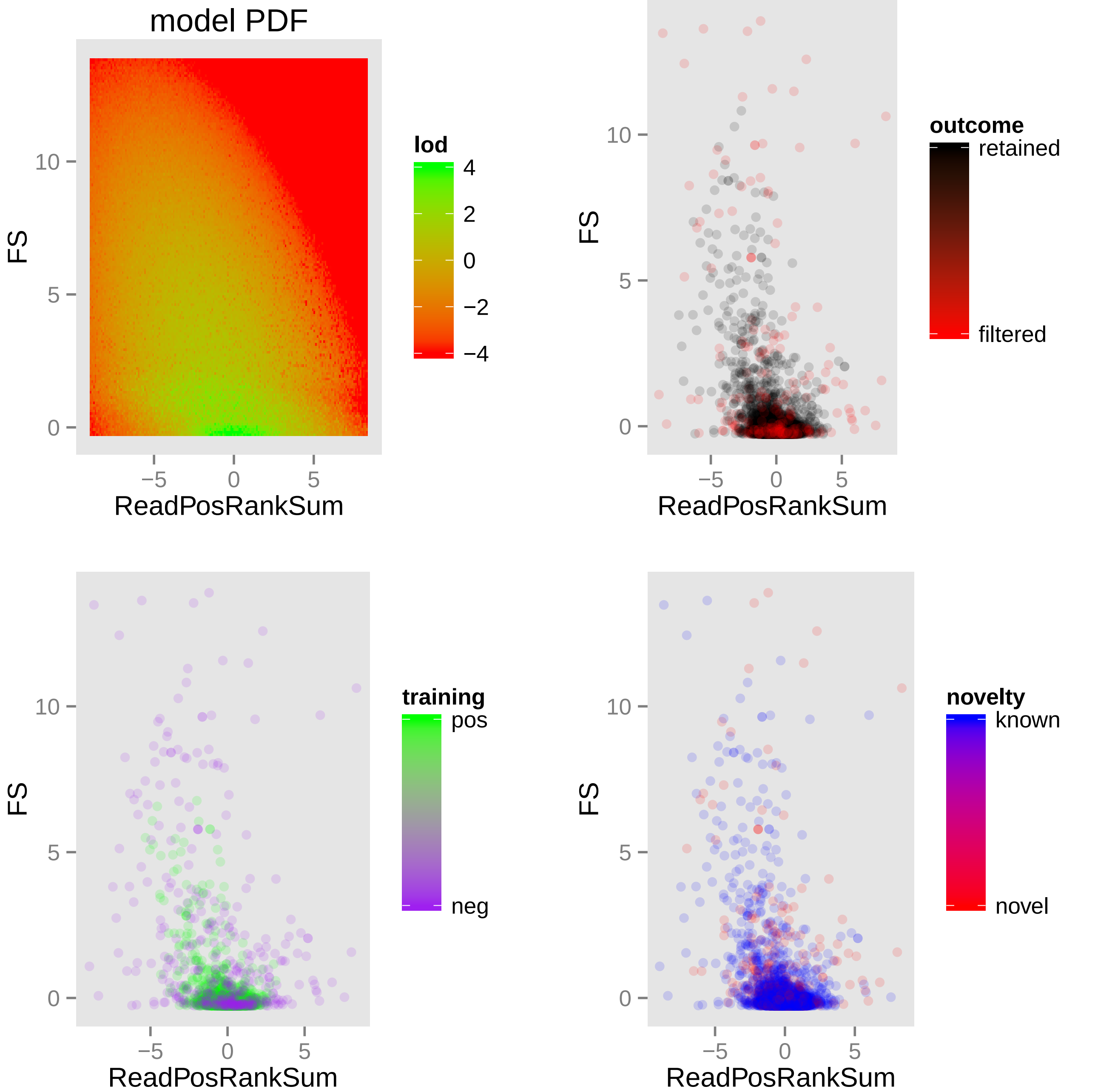
model PDF
lod
4
2
0
−2
−4
FS
ReadPosRankSum
outcome
retained
filtered
training
pos
neg
novelty
known
novel
10
5
0
−5
0
5

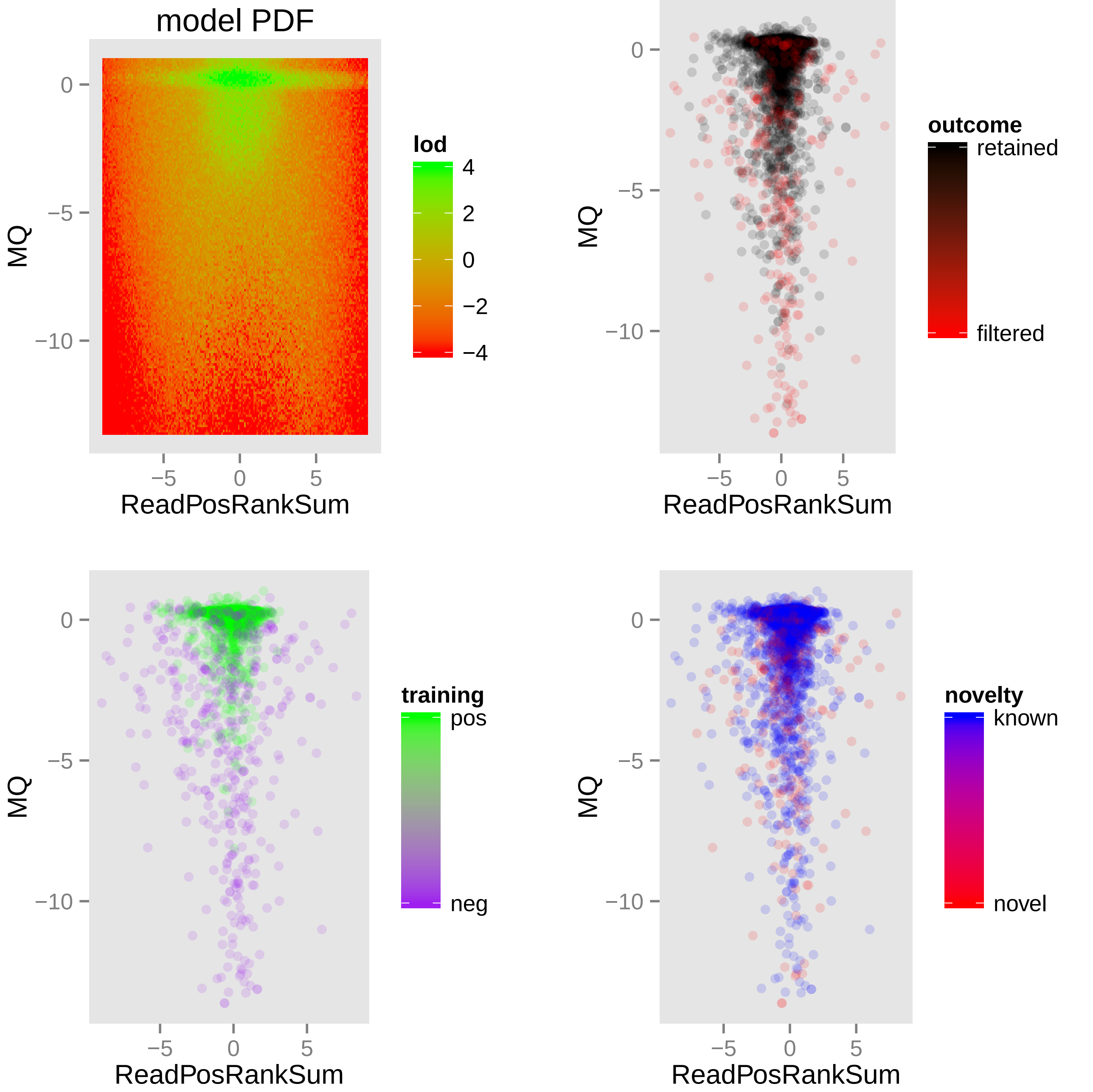

model PDF
lod
4
2
0
−2
−4
MQ
0
−5
−10
ReadPosRankSum
−5
0
5
outcome
retained
filtered
training
pos
neg
novelty
known
novel

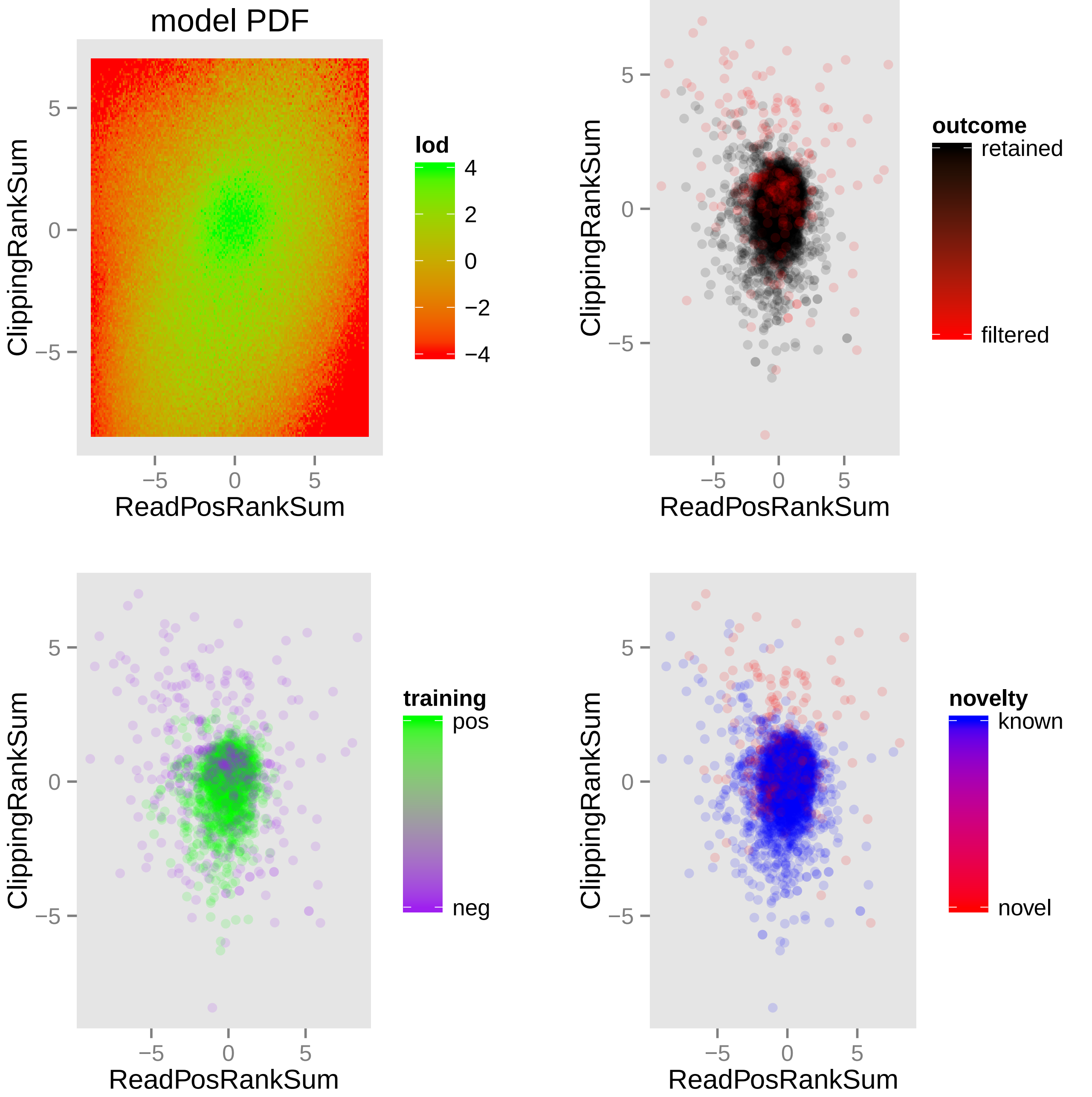

model PDF
lod
4
2
0
−2
−4
ClippingRankSum
ReadPosRankSum
outcome
retained
filtered
training
pos
neg
novelty
known
novel

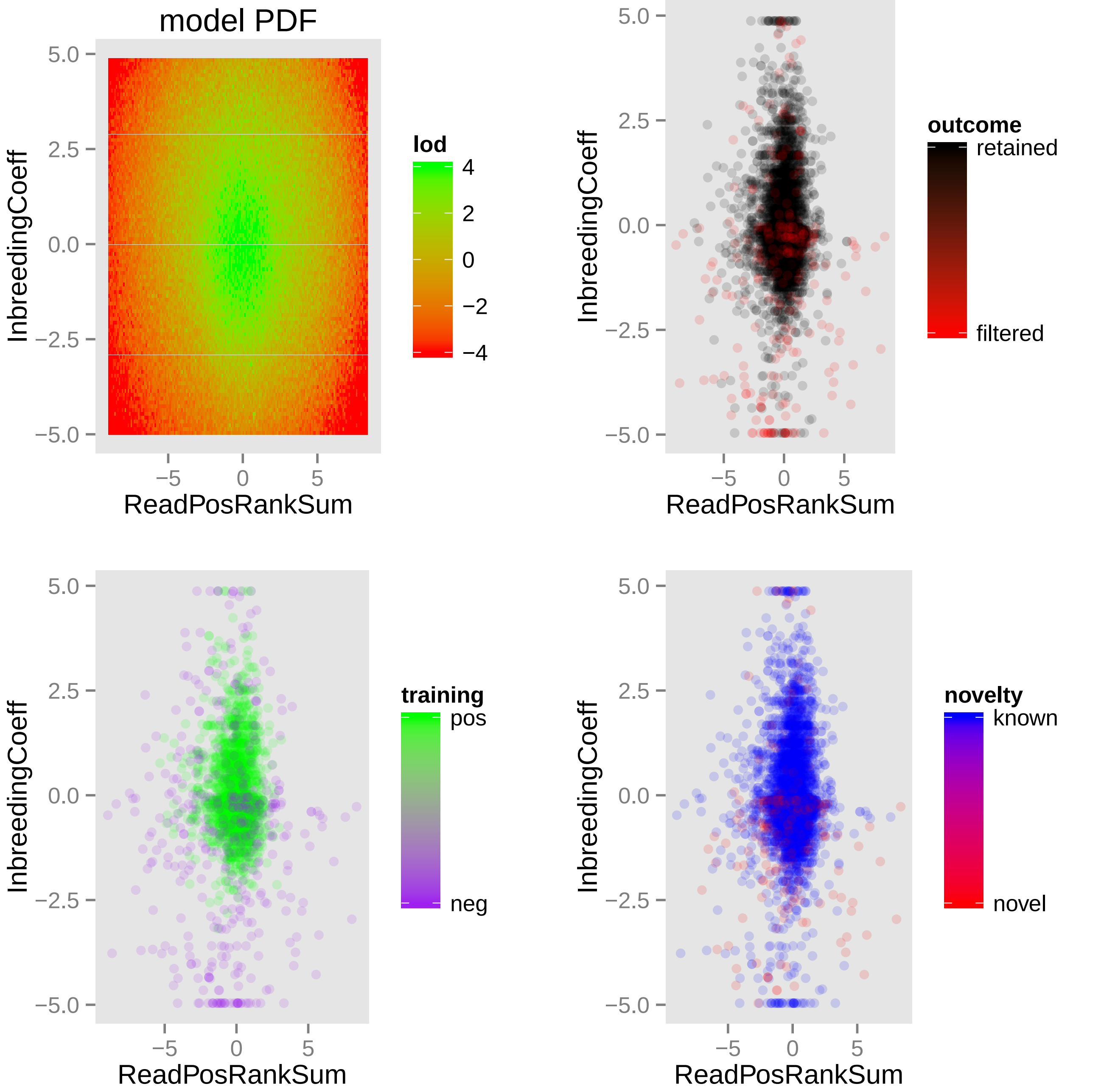

model PDF
InbreedingCoeff
ReadPosRankSum
lod
4
2
0
−2
−4
5.0
2.5
0.0
−2.5
−5.0
−5
0
5
outcome
retained
filtered
training
pos
neg
novelty
known
novel

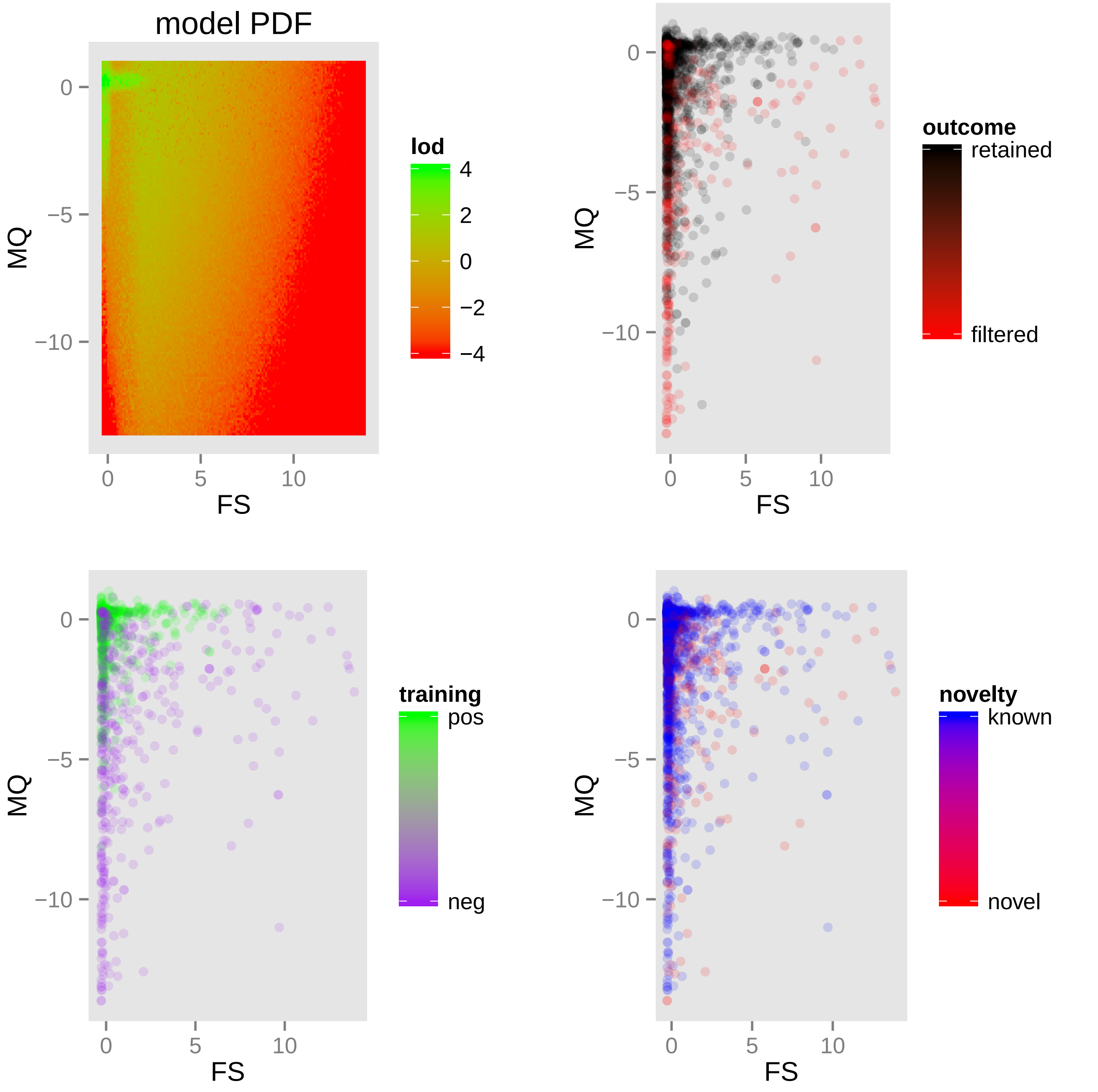

model PDF
lod
4
2
0
−2
−4
MQ
FS
outcome
retained
filtered
training
pos
neg
novelty
known
novel

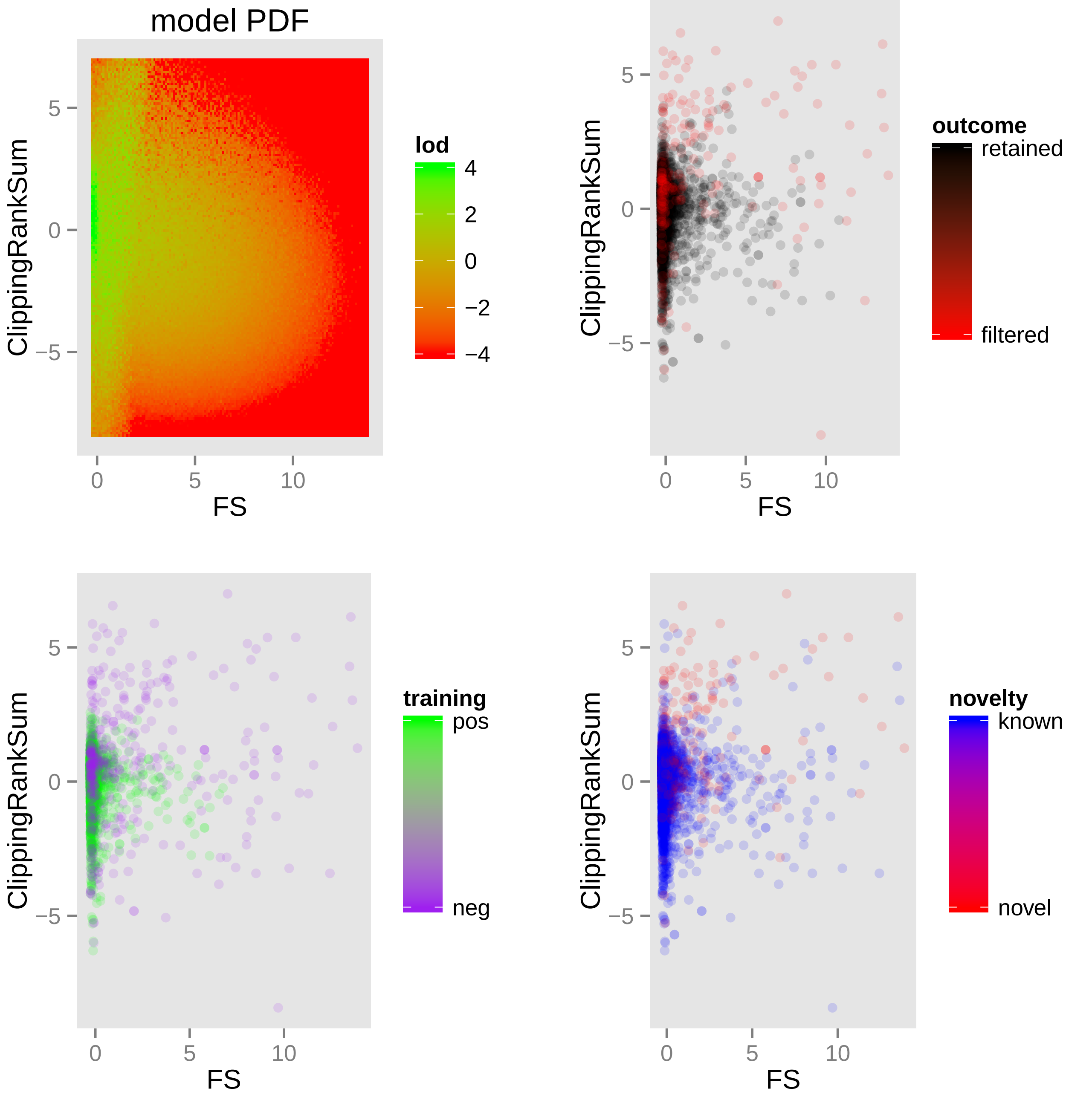

model PDF
ClippingRankSum
FS
lod
4
2
0
−2
−4
outcome
retained
filtered
training
pos
neg
novelty
known
novel

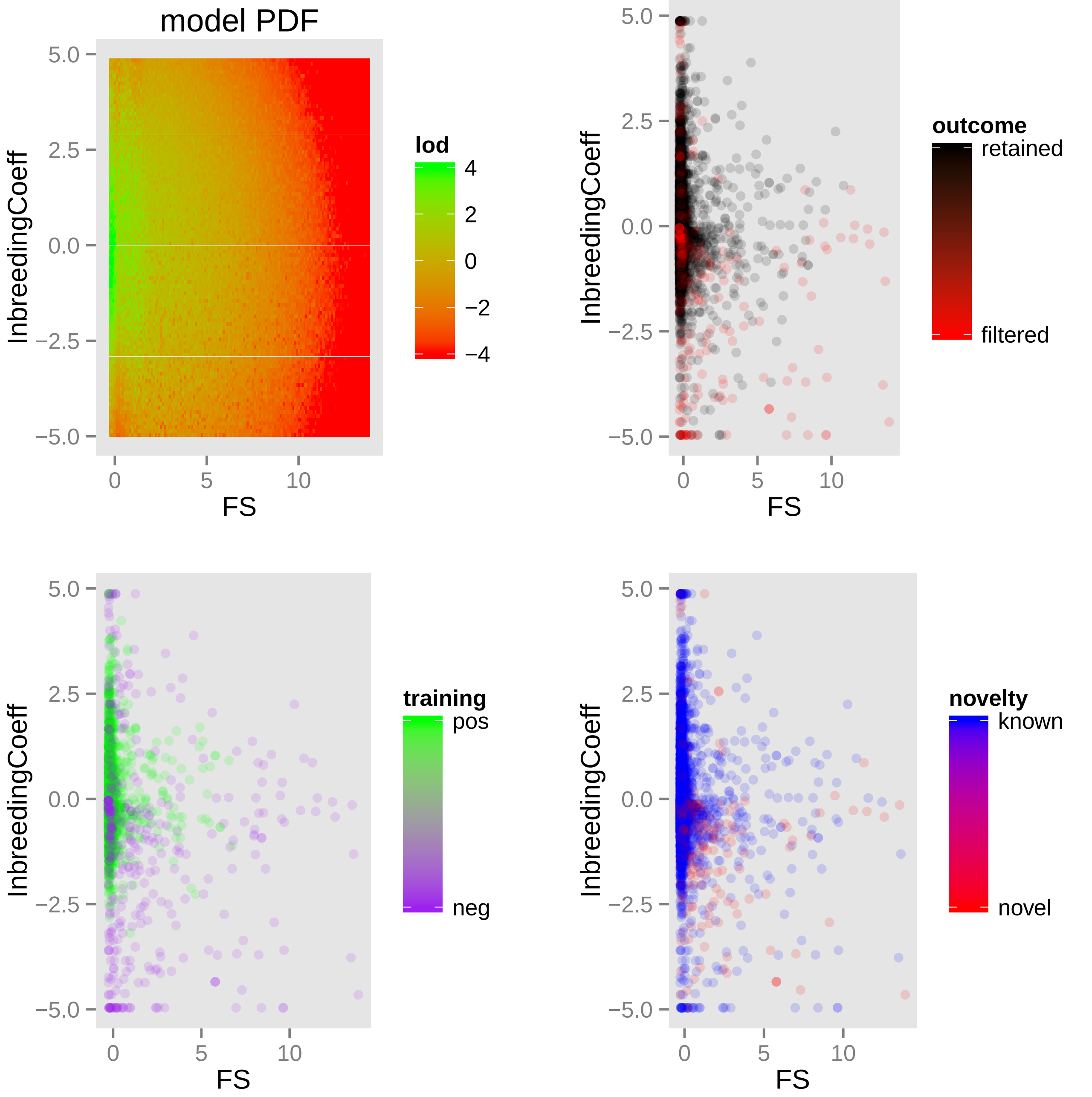
model PDF
InbreedingCoeff
FS
lod
4
2
0
−2
−4
outcome
retained
filtered
training
pos
neg
novelty
known
novel
5.0
2.5
0.0
−2.5
−5.0
0
5
10

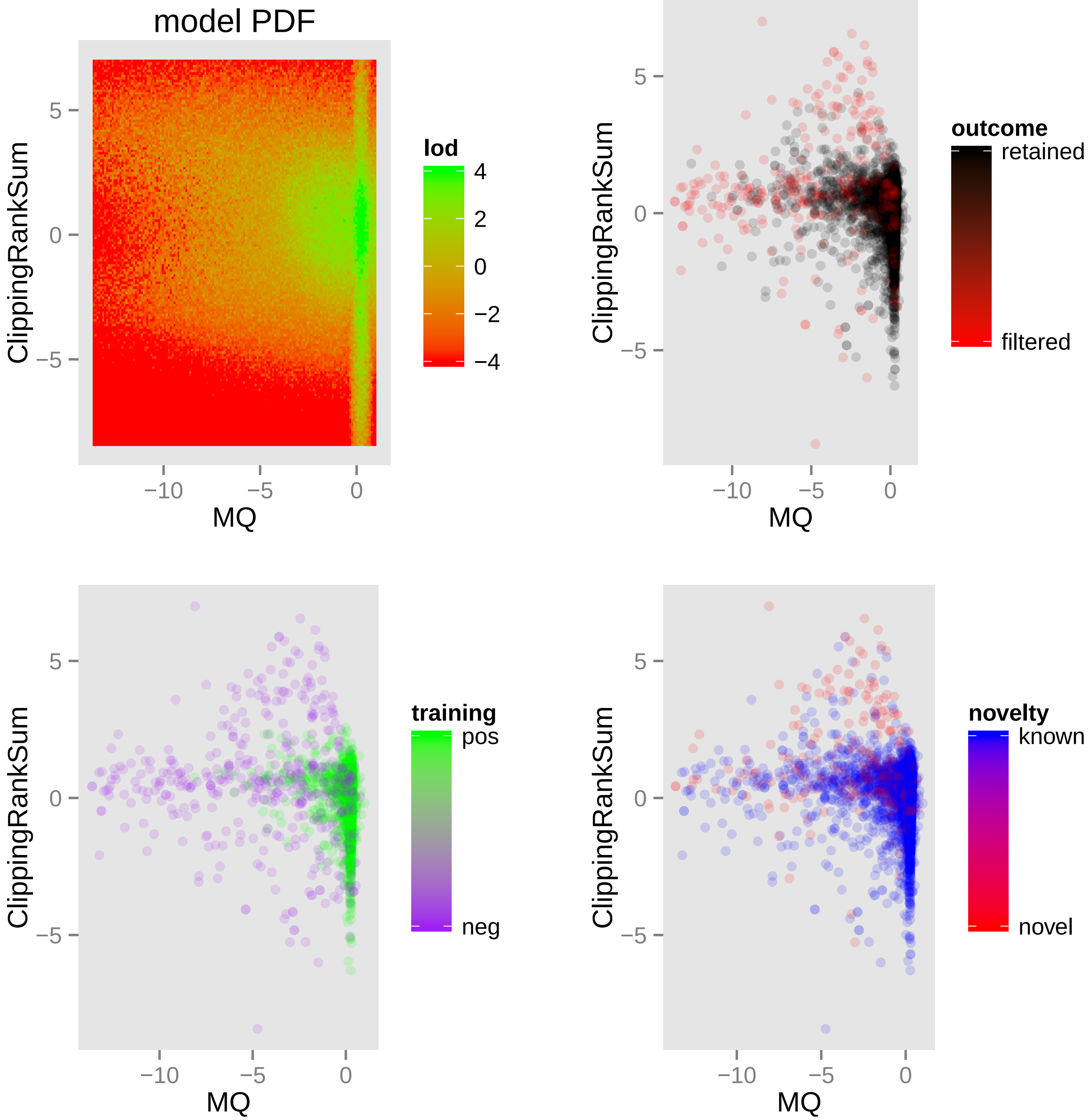

model PDF
ClippingRankSum
MQ
lod
4
2
0
−2
−4
outcome
retained
filtered
training
pos
neg
novelty
known
novel
5
0
−5
−10

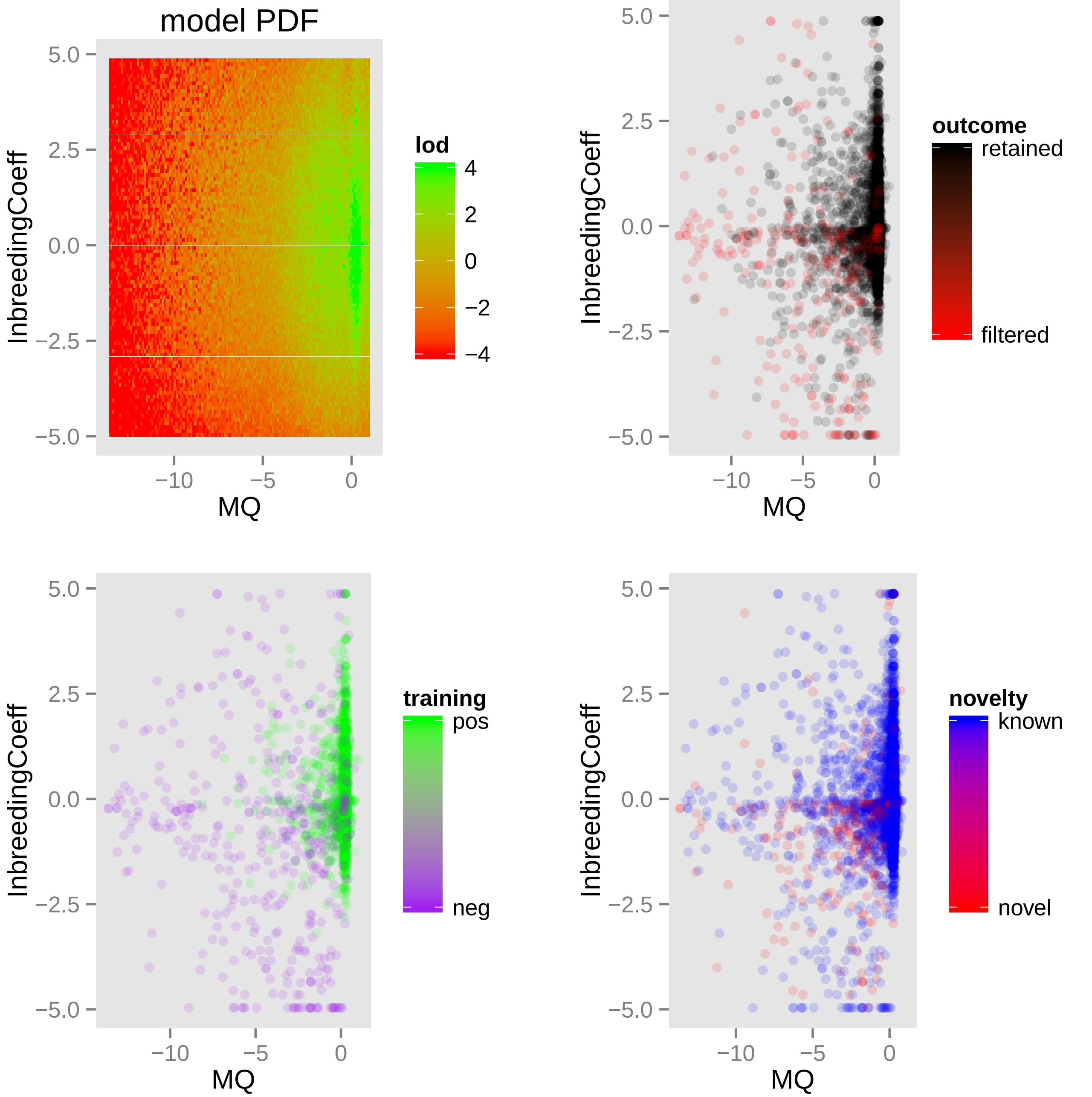

model PDF
lod
4
2
0
−2
−4
outcome
retained
filtered
training
pos
neg
novelty
known
novel
InbreedingCoeff
MQ
5.0
2.5
0.0
−2.5
−5.0
−10
−5
0

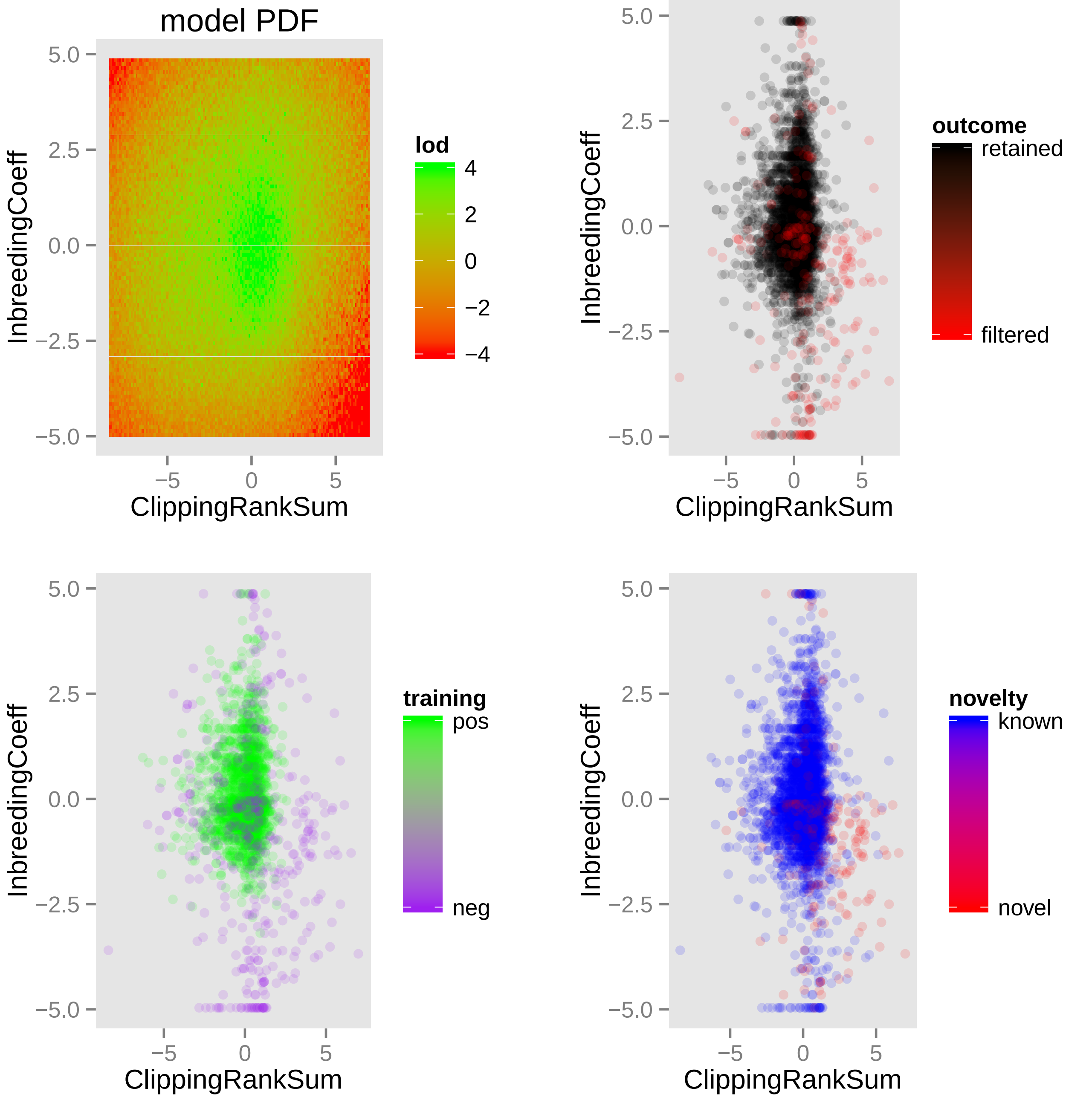

model PDF
InbreedingCoeff
ClippingRankSum
lod
4
2
0
−2
−4
5.0
2.5
0.0
−2.5
−5.0
−5
0
5
outcome
retained
filtered
training
pos
neg
novelty
known
novel
