# Supplementary material for: Identification and validation of loss of function variants in clinical contexts
Source: Mol Genet Genomic Med. 2013 Oct 11;2(1):58–63. doi: 10.1002/mgg3.42 (PMC3907911; doi:10.1002/mgg3.42)
Supplement: Supplementary file 6 — Table S1Design. Table S2Validation of variants by caller. Table S3Performance of the new HaplotypeCaller version on genotyped variants. [file mgg30002-0058-sd6.docx]

**Supplementary Table 1**

|  |  | **Intersection** | **UnifiedGenotyper only** | **HaplotypeCaller only** |
| --- | --- | --- | --- | --- |
| SNPs | Called | 170 | 53 | 18 |
|  | Total designed | 102 (60.0%) | 28 (52.8%) | 10 (55.6%) |
|  | Total assays worked | 99 (58.2%) | 28 (52.8%) | 9 (50.0%) |
| INDELs | Called | 269 | 108 | 228 |
|  | Total designed | 42 (15.6%) | 12 (11.1%) | 42 (18.4%) |
|  | Total assays worked | 38 (14.1%) | 12 (11.1%) | 30 (13.2%) |

**Supplementary Table 1: Design**

The performance of the assay design is given, comparing the rates among the calls identified by both methods or by one caller only.

**Supplementary Table 2**

|  | Outcome | **UnifiedGenotyper total** | **HaplotypeCaller total** |
| --- | --- | --- | --- |
| SNPs | Validated | 124 (97.6%) | 99 (91.7%) |
|  | Not validated | 3 (2.4%) | 9 (8.3%) |
|  | Fail | 3 | 4 |
|  | Total number assays | 130 | 112 |
|  | Total working assays | 127 | 108 |
| INDELs | Validated | 46 (92.0%) | 38 (55.9%) |
|  | Not validated | 4 (8.0%) | 30 (44.1%) |
|  | Fail | 4 | 16 |
|  | Total designed assays | 54 | 84 |
|  | Total working assays | 50 | 68 |

**Supplementary Table 2: Validation of variants by caller**

The validation results are given by caller, including the number of designed assays against those that failed on the genotyping chip.

**Supplementary Table 3**

|  | **SNPs** | | | |  |
| --- | --- | --- | --- | --- | --- |
| Genotyped loci also called by HaplotypeCaller2.4.9 | All 3 Intersection | Common HaplotypeCaller2.4.9 and HaplotypeCaller2.2.2 | | Common HaplotypeCaller2.4.9 and UnifiedGenotyper |  |
| validated | 93 (97.9%) | 2 (100%) | | 17 (100%) | 112 |
| not validated | 2 (2.1%) | 0 (0.0%) | | 0 (0.0%) | 2 |
| Nas | 4 | 1 | | 0 | 5 |
| total working assays | 95 | 2 | | 17 | 114 |
| total designed assays | 99 | 3 | | 17 | 119 |
|  |  |  | | not called | 14 (12.5%) |
|  |  |  | | total | 133 |
|  |  |  | |  |  |
|  |  |  | |  |  |
|  | **INDELs** | | | |  |
| Genotyped loci also called by HaplotypeCaller2.4.9 | All 3 Intersection | | Common HaplotypeCaller2.4.9 and HaplotypeCaller2.2.2 | Common HaplotypeCaller2.4.9 and UnifiedGenotyper |  |
| validated | 28 (93.3%) | | 3 (60.0%) | 7 (100%) | 38 |
| not validated | 2 (6.7%) | | 2 (40.0%) | 0 (0.0%) | 4 |
| Nas | 3 | | 1 | 0 | 4 |
| total withot NA | 30 | | 5 | 7 | 42 |
| total with NA | 33 | | 6 | 7 | 46 |
|  |  | |  | not called | 2 (5.3%) |
|  |  | |  | total | 48 |

**Supplementary Table 3: Performance of the new HaplotypeCaller version on genotyped variants**

Among the variants genotyped during the validation phase, results are grouped based on the overlap between the variants identified by the new version of HaplotyCaller and the other algorithms. SNPs and INDELs are reported, as well as the number of variants genotyped but not called by the new version.

**Supplementary Figure 1: Workflow**

The flow diagram indicates the different selection steps in our workflow, and the number of variants filtered in each steps, until the final design and genotyping.

**Supplementary Figure 2: Overlap with the new HaplotypeCaller version**

Overlap of Loss of Function variants identified by the UnifiedGenotyper algorithm and two versions of HaplotypeCaller for A) single nucleotide polimorphysms and B) insertions/deletions.
